# Divergent co-transcriptomes of different host cells infected with *Toxoplasma gondii* reveal cell type-specific host-parasite interactions

**DOI:** 10.1038/s41598-017-07838-w

**Published:** 2017-08-03

**Authors:** Izabela J. Swierzy, Ulrike Händel, Alexander Kaever, Michael Jarek, Maren Scharfe, Dirk Schlüter, Carsten G. K. Lüder

**Affiliations:** 1 Institute for Medical Microbiology, University Medical Center, Georg-August-University, 37075 Göttingen, Germany; 2 Institute of Medical Microbiology, Infection Control and Prevention, Otto-von-Guericke-University, 39120 Magdeburg, Germany; 3 Institute of Microbiology and Genetics, Department of Bioinformatics, Georg-August-University, 37077 Göttingen, Germany; 4 Genome Analytics, Helmholtz Centre for Infection Research, 38124 Braunschweig, Germany; 5 Organ-Specific Immune Regulation, Helmholtz Centre for Infection Research, 38124 Braunschweig, Germany

## Abstract

The apicomplexan parasite *Toxoplasma gondii* infects various cell types in avian and mammalian hosts including humans. Infection of immunocompetent hosts is mostly asymptomatic or benign, but leads to development of largely dormant bradyzoites that persist predominantly within neurons and muscle cells. Here we have analyzed the impact of the host cell type on the co-transcriptomes of host and parasite using high-throughput RNA sequencing. Murine cortical neurons and astrocytes, skeletal muscle cells (SkMCs) and fibroblasts differed by more than 16,200 differentially expressed genes (DEGs) before and after infection with *T. gondii*. However, only a few hundred of them were regulated by infection and these largely diverged in neurons, SkMCs, astrocytes and fibroblasts indicating host cell type-specific transcriptional responses after infection. The heterogeneous transcriptomes of host cells before and during infection coincided with ~5,400 DEGs in *T. gondii* residing in different cell types. Finally, we identified gene clusters in both *T. gondii* and its host, which correlated with the predominant parasite persistence in neurons or SkMCs as compared to astrocytes or fibroblasts. Thus, heterogeneous expression profiles of different host cell types and the parasites’ ability to adapting to them may govern the parasite-host cell interaction during toxoplasmosis.

## Introduction

*Toxoplasma gondii* is an intracellular parasite of the *Apicomplexa* that comprise several pathogens of utmost importance for humans and animals. *T. gondii* itself is globally distributed and one of the most common human parasites infecting up to one third of the world population. Although infections are mostly asymptomatic or benign, the high prevalence makes *T. gondii* a significant threat for human health^1^. Complications of *T. gondii* infections include retinochoroiditis in immunocompetent adults, severe to even life-threatening congenital toxoplasmosis after infection *in utero* and reactivated *Toxoplasma* encephalitis in immunocompromised patients^2^.

One of the outstanding features of *T. gondii* is its extraordinary broad host and host cell spectrum^3, 4^. After oral uptake of infectious stages via contaminated food or from the environment, they transform into fast replicating tachyzoites that are able to infect and replicate in any nucleated cell of any mammalian or avian host. Promiscuous host cell invasion is accomplished by a parasite-driven process which relies on the parasites’ actin-myosin motor complex and multi-protein complexes secreted by *T. gondii* and assembled within the host cell membrane^5, 6^. Although distinct cell types including monocytic cells may be more prone to infection than others^7^, invasion of any nucleated cell type supports parasite propagation eventually leading to acute toxoplasmosis. Immunoreactive tachyzoites are subsequently largely eradicated by the ensuing pro-inflammatory response of the host, but few of them transform into a latent parasite stage. These so-called bradyzoites are metabolically largely inactive, are mostly within the G_0_ phase of the cell cycle and form tissue cysts which can persist for the hosts’ life preferably within neuronal and muscle cells^8–10^. In the case of immunosuppression, latent bradyzoites can transform to replicative tachyzoites leading to necrotizing tissue destruction and overt disease^2^.

The impact of the host cell type on the parasite and *vice versa* has not yet been thoroughly elucidated. Furthermore, the molecular and cellular mechanisms which are responsible for preferred localization of *T. gondii* tissue cysts in neural and muscular tissues remain elusive. The fact that tissue cysts develop concomitantly with the ensuing pro-inflammatory response *in vivo* has led to the hypothesis that immunity-related stress factors, e.g. reactive oxygen and nitrogen species or nutrient depletion triggers differentiation towards the bradyzoite stage in diverse host tissues^11, 12^. An alternative hypothesis suggests that neuronal and muscular cells provide a suitable cellular microenvironment that triggers bradyzoite formation in *T. gondii* and hence favors parasite persistence^13^. Neurons and muscle cells indeed trigger bradyzoite formation and tissue cyst development in the absence of exogenous stressors^14, 15^. We recently discovered that mature syncytial myotubes but not proliferating myoblasts spontaneously sustain tissue cyst formation and this required the negative host cell cycle regulator Tspyl2^16^.

In order to determine cell type-specific responses of *T. gondii* and its mammalian host we analyzed genome-wide transcriptomes of four different host cell types, namely skeletal muscle cells (SkMCs), neurons, astrocytes and fibroblasts after infection. Analysis of non-infected host cells enabled us to identify expression profiles and/or biological pathways that may contribute to triggering stage differentiation of *T. gondii* in neurons and SkMCs but not in astrocytes and fibroblasts. Remarkably, our results for the first time indicate a highly divergent host cell response to infection with *T. gondii*. Furthermore, the transcriptional profile of *T. gondii* also differed substantially after infection of different host cells. This suggests that the parasite-host-interaction during toxoplasmosis strongly differs depending on the type of infected host cell. We also identified common host cell and parasite candidate pathways which might trigger bradyzoite formation in *T. gondii*. These results are of general importance with respect to pathogens infecting different host cells.

## Results

### Host cell responses to *T. gondii* infection are largely cell type-specific

Transcriptional responses of mammalian cells to *T. gondii* infection may govern the parasite-host interaction, but the impact of infection on the transcriptomes of different host cell types is unknown. Here, we used high-throughput Illumina sequencing in order to compare expression profiles of mouse SkMCs, neurons, astrocytes and fibroblasts infected or not with an avirulent type II *T. gondii* strain for 24 hours. Control immunofluorescence staining confirmed formation and purity of mature syncytial myosin heavy chain (MyHC)-positive myotubes, class III β-tubulin-positive neurons, glial fibrillary acidic protein (GFAP)-positive astrocytes and pan actin-positive fibroblasts (see Supplementary Fig. S1). Concomitant *T. gondii* staining revealed similar infection intensities in all cell types (Supplementary Fig. S1).

**Figure 1:**
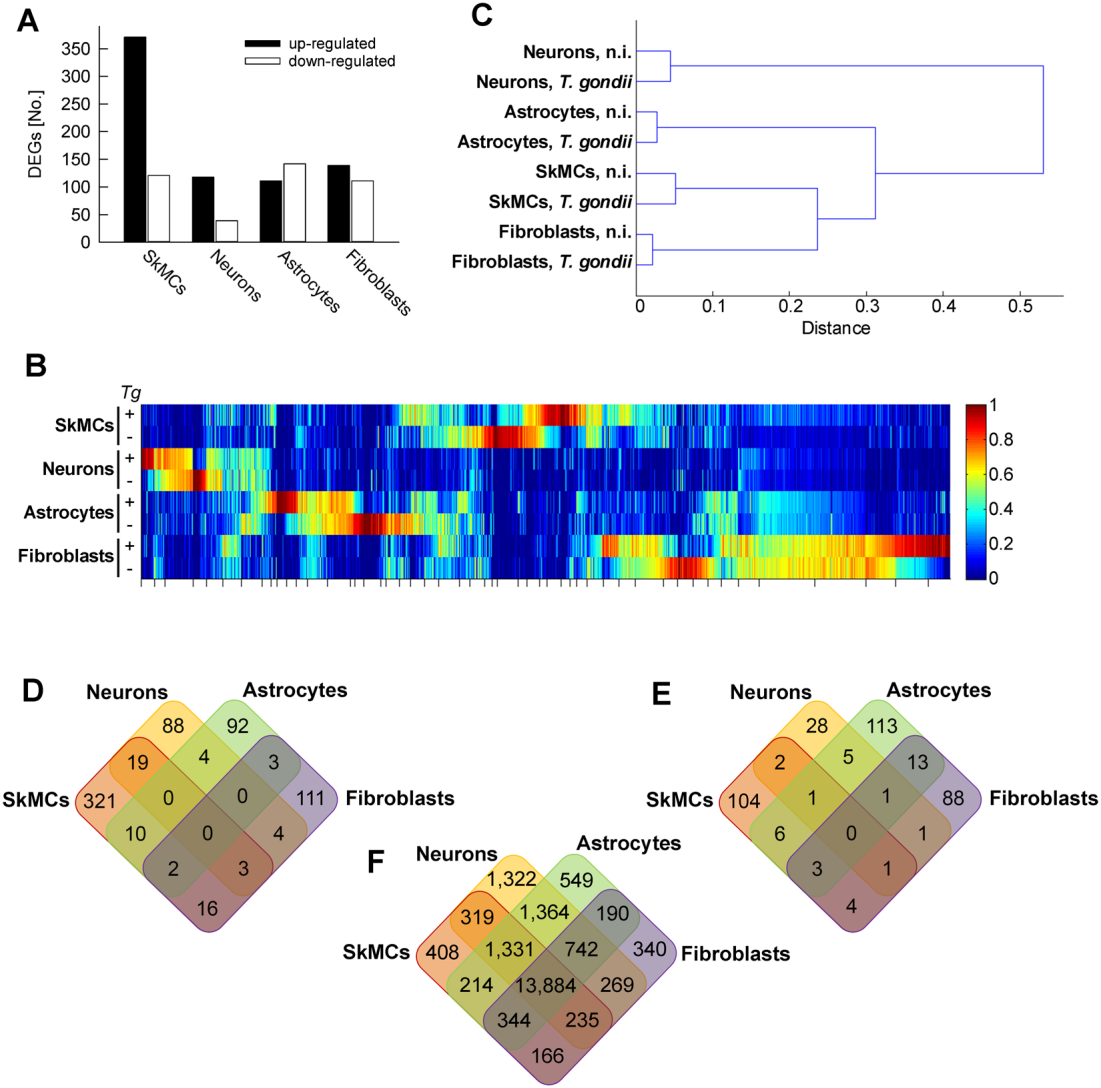
Host cell type-specific responses of mouse SkMCs, neurons, astrocytes and fibroblasts to infection with *T. gondii*. Mature C2C12 myotubes, cortical neurons and astrocytes, and NIH/3T3 fibroblasts were infected with *T. gondii* at a MOI of 5:1 for 24 hours or were left non-infected. Pools of total RNA from four different biological replicates each were used to prepare cDNA libraries which were then sequenced using Illumina technology. Reads were mapped to the *Mus musculus* reference genome. (**A**) Numbers of differentially expressed genes (DEGs) which were up- or down-regulated (>2-fold, *p* < 0.05) after *T. gondii* infection as compared to the respective non-infected control. (**B**) Heatmap of DEGs after infection of SkMCs, neurons, astrocytes and fibroblasts with *T. gondii*. Expression profiles were clustered according to a one-dimensional self-organizing map. (**C**) Hierarchical cluster analysis of genes which were differentially expressed after *T. gondii* infection in at least one host cell type. (**D**) Venn diagram of genes up-regulated (>2-fold, *p* < 0.05) by *T. gondii* in at least one of the four host cell types. (**E**) Venn diagram of genes down-regulated (>2-fold, *p* < 0.05) by *T. gondii* in at least one of the four host cell types. (**F**) Venn diagram of genes expressed in any of the four different non-infected host cell types.

Between ~50% and 85% of the sequencing reads mapped to the *Mus musculus* reference genome and this did not differ between infected and non-infected cDNA libraries (see Supplementary Table S1). Using the MarVis filtering and visualization software^17, 18^, 16,282 mouse genes were identified being differentially expressed between cell types or after infection (moderated Chi^2^ test, FWER < 0.01). RNAseq data were experimentally validated by RT-quantitative real-time PCR. Such comparison revealed a correlation coefficient of r = 0.922, indicating an overall high level of concordance between both methods (see Supplementary Fig. S2). It should be mentioned however that for a minor number of individual genes in distinct cell types, RNAseq and RT-qPCR data indicated different levels of regulation after infection (e.g., Cxcl10 and Ttpal in fibroblasts; see Supplementary Fig. S2A). Among the more than 16,000 differentially expressed genes (DEGs), only 492 (SkMCs) to 157 (neurons) were at least 2-fold regulated after infection with *T. gondii* (Chi^2^ *p* < 0.05; Fig. 1A; for a list of regulated genes after infection and their expression in the different cell types see Supplementary Tables S2, S3, S4 and S5). Strikingly, even among these *Toxoplasma*-regulated host genes, the expression profiles largely differed between SkMCs, neurons, astrocytes and fibroblasts (Fig. 1B). A hierarchical cluster analysis of the DEG profiles revealed clear differences between the four different cell types whereas infection with *T. gondii* had only minor impact on the host cell expression profiles (Fig. 1C). Thus, after infection with *T. gondii*, the transcriptomes of SkMCs, neurons, astrocytes and fibroblasts were still largely cell type-specific suggesting that the parasite may encounter fundamentally different environments within different host cell types. SkMCs and fibroblasts clustered more closely together as compared to the other two cell types, and neurons showed the largest distance to the other three cell types (Fig. 1C).

Venn analysis of the genes up- or down-regulated after infection revealed a largely heterogeneous transcriptional response of SkMCs, neurons, astrocytes and fibroblasts to *T. gondii* (Fig. 1D,E). The vast majority of DEGs were indeed regulated after *T. gondii* infection in a strictly host cell type-specific manner. Thus, only few genes were commonly up- or down-regulated in two or three cell types and none was regulated in all four cells types after infection (Fig. 1D,E). In contrast, genes being expressed in either of the different cell types (RPKM > 0) largely overlapped between SkMCs, neurons, astrocytes and fibroblasts (Fig. 1F).

**Figure 2:**
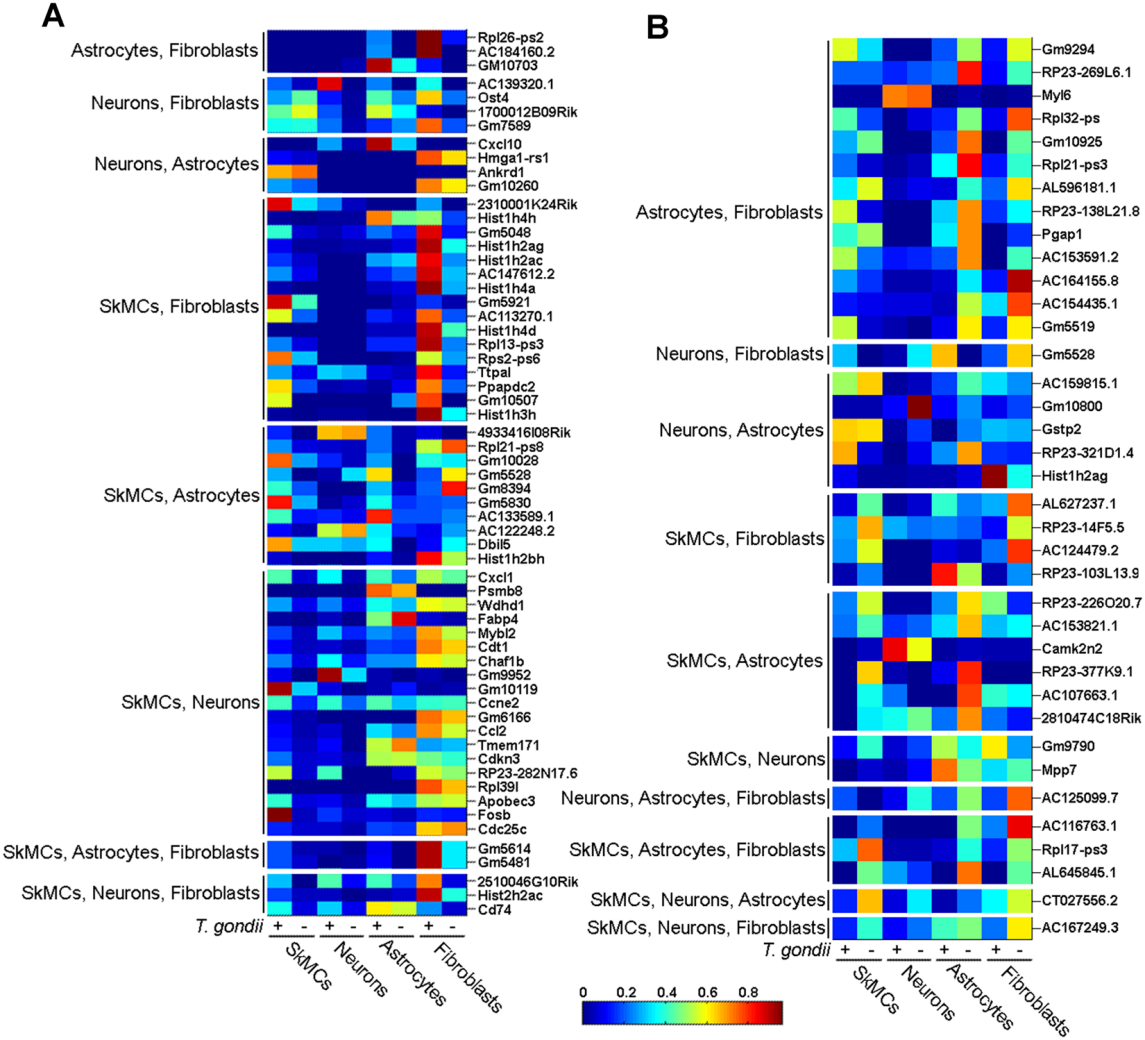
Common transcriptional responses of SkMCs, neurons, astrocytes and fibroblasts after infection with *T. gondii* as revealed by RNA high-throughput sequencing. Genes that were commonly regulated in at least two cell types after infection with *T. gondii* (>2-fold; *p* < 0.05) were clustered according to host cell groups, and expression intensities were visualized using heat maps. (**A**) Genes commonly up-regulated in multiple cell types after infection. (**B**) Genes commonly down-regulated in multiple cell types after infection.

We next inspected conserved and divergent responses of SkMCs, neurons, astrocytes and fibroblasts to *T. gondii* infection. Genes commonly up-regulated in at least two or three host cell types comprised several histone variants (Fig. 2A). Interestingly, transcript levels of genes regulating DNA replication and cell cycle progression (*Cdc25c*, *Fosb*, *Cdkn3*, *cyclin E2*, *Chaf1b*, *Cdt1*) were specifically increased in SkMCs and neurons after infection. In contrast, only a few immunity-related mRNAs encoding chemokines (Cxcl10, Cxcl1, Ccl2) and the invariant chain (CD74) were up-regulated in multiple host cell types after infection (Fig. 2A). Overall, although several mRNAs were similarly regulated after *T. gondii* infection in two or three cell types, they mostly exhibited substantial differences in their absolute expression intensities in these cells (Fig. 2A,B).

Host cell genes, the expression of which was regulated by *T. gondii* infection in a strictly cell-type specific manner accounted for between ~74% in neurons and up to ~86% in SkMCs of all DEGs in the respective cell type. Interestingly, among the transcripts which were specifically and highly increased in SkMCs following infection was the adenosine receptor A_2A_ (Adora2a; 16.5-fold up-regulated) (see Supplementary Table S2). Furthermore, Adora2a mRNA was more abundant in neurons irrespective of infection as compared to astrocytes and fibroblasts (Supplementary Table S2). Generation of extracellular adenosine by CD73 and probably its uptake by adenosine receptors has been shown to promote *Toxoplasma* cyst formation in the brain^19^. Transcript levels of the immediate early response genes *Egr1* and *Egr2*, *Fos*, *Junb* and *Myc* were also significantly up-regulated in SkMCs but not in neurons, astrocytes or fibroblasts (see Supplementary Table S2). They belong to a group of transcription factors that regulate cell survival and cell proliferation and are up-regulated in response to growth factors or *T. gondii* infection in human fibroblasts^20^.

**Figure 3:**
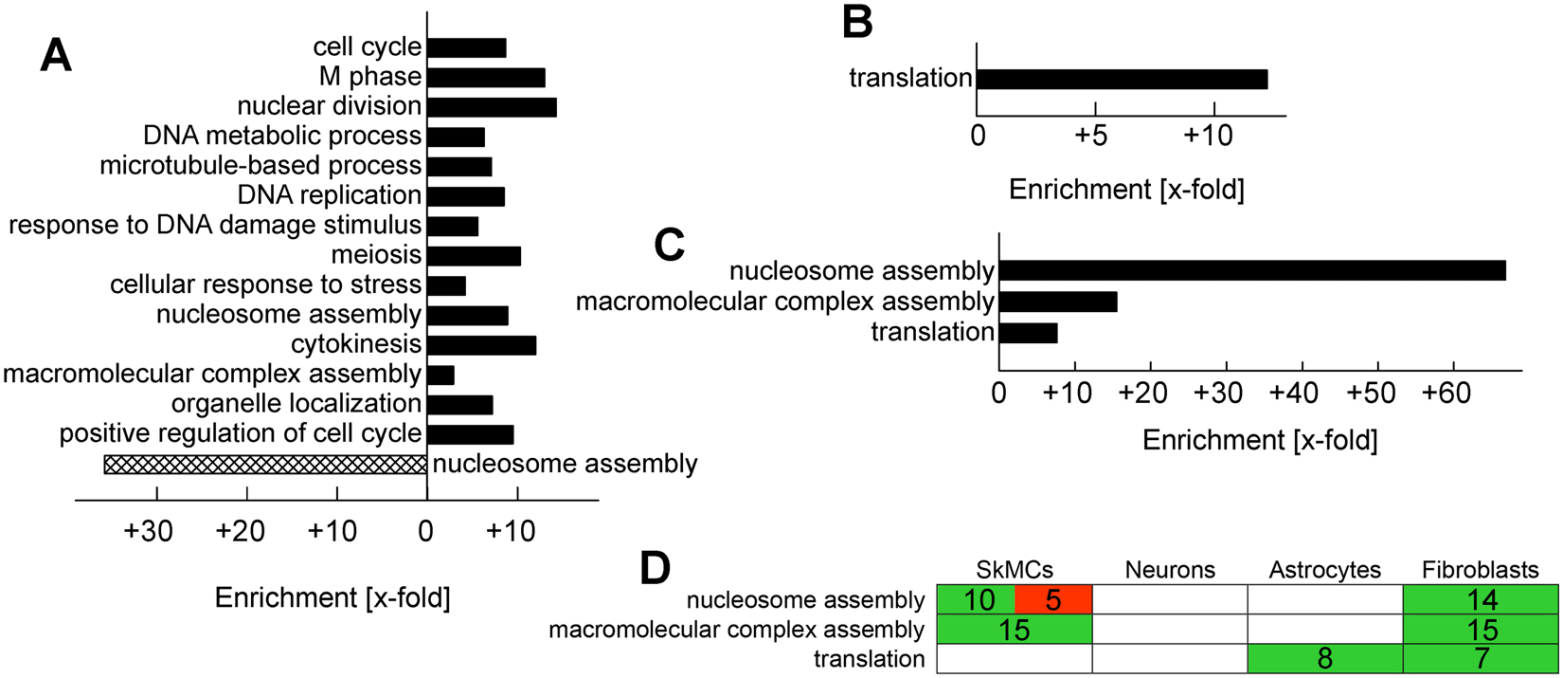
Biological processes differentially or commonly enriched in the *T. gondii*-regulated transcriptomes of mature SkMCs, neurons, astrocytes and fibroblasts. After genome-wide expression profiling, *T. gondii*-regulated host cell genes (at least 2-fold up- or downregulation; *p* < 0.05) were screened for annotation terms being overrepresented as compared to the reference genome to identify biological processes being regulated after infection (FDR < 0.05, modified Fisher exact test with Benjamini-Hochberg correction for multiple testing). Redundant annotation terms were omitted. (**A**) Annotation terms that are overrepresented among genes up- (black bars) or downregulated (cross-hatches bars) in SkMCs by *T. gondii*-infection. (**B**) Annotation term that is overrepresented among genes upregulated in astrocytes by *T. gondii*-infection. (**C**) Annotation terms that are overrepresented among genes upregulated in fibroblasts by *T. gondii*-infection. (**D**) Impact of *T. gondii* infection on biological processes being enriched in at least two cell types following infection. Up- and downregulation of genes related to the biological processes as indicated are depicted in *green* or *red*, respectively. Figures indicate the numbers of genes the expression of which is regulated in the respective cell types.

The regulation of largely distinctive gene profiles after infection of different host cell types with *T. gondii* does not necessarily exclude common signatures of the host cell responses. Enrichment of annotation terms among the DEGs of the *T. gondii*-regulated expression profiles was therefore analysed in order to identify biological processes that were modulated after infection. Most genes that were up-regulated in infected SkMCs as compared to non-infected controls were related to the cell cycle (82 genes, 8.7-fold enriched) and particularly to the M phase of the cell cycle (57 genes, 13-fold enriched; Fig. 3A). We also identified 9 cell cycle-related genes whose expression was up-regulated in neurons but not in astrocytes and fibroblasts (*Hmga2*, *Anxa1*, *Chaf1b*, *S100a6*, *Cdt1*, *Cdc25c, Gmnn*, *Cdkn3*, *Ccne2*; see Supplementary Table S3) however, this number was not significant (2.6-fold enriched; *p* > 0.05). It nevertheless suggests that the host cell cycle is modulated by infection with *T. gondii* in SkMCs and neurons but not in astrocytes and fibroblasts. Genes related to responses to DNA damage (25 genes) or cellular responses to stress (26 genes) were also specifically enriched in SkMCs (5.6- and 4.2-fold, respectively; Fig. 3A). Nucleosome assembly and macromolecular complex assembly were processes that were commonly regulated in SkMCs and fibroblasts (Fig. 3D). Finally, translation was a biological process that was enriched in the *Toxoplasma*-induced transcriptomes of both astrocytes and fibroblasts (Fig. 3B–D). Together, these data indicate that the transcriptional responses of SkMCs, neurons, astrocytes and fibroblasts to *T. gondii* are largely cell type-specific. Furthermore, only a few biological processes were identified that were commonly regulated after infection of two or more cell types after infection with *T. gondii*.

### Common transcriptome signatures in SkMCs and neurons versus astrocytes and fibroblasts prior to infection

**Figure 4:**
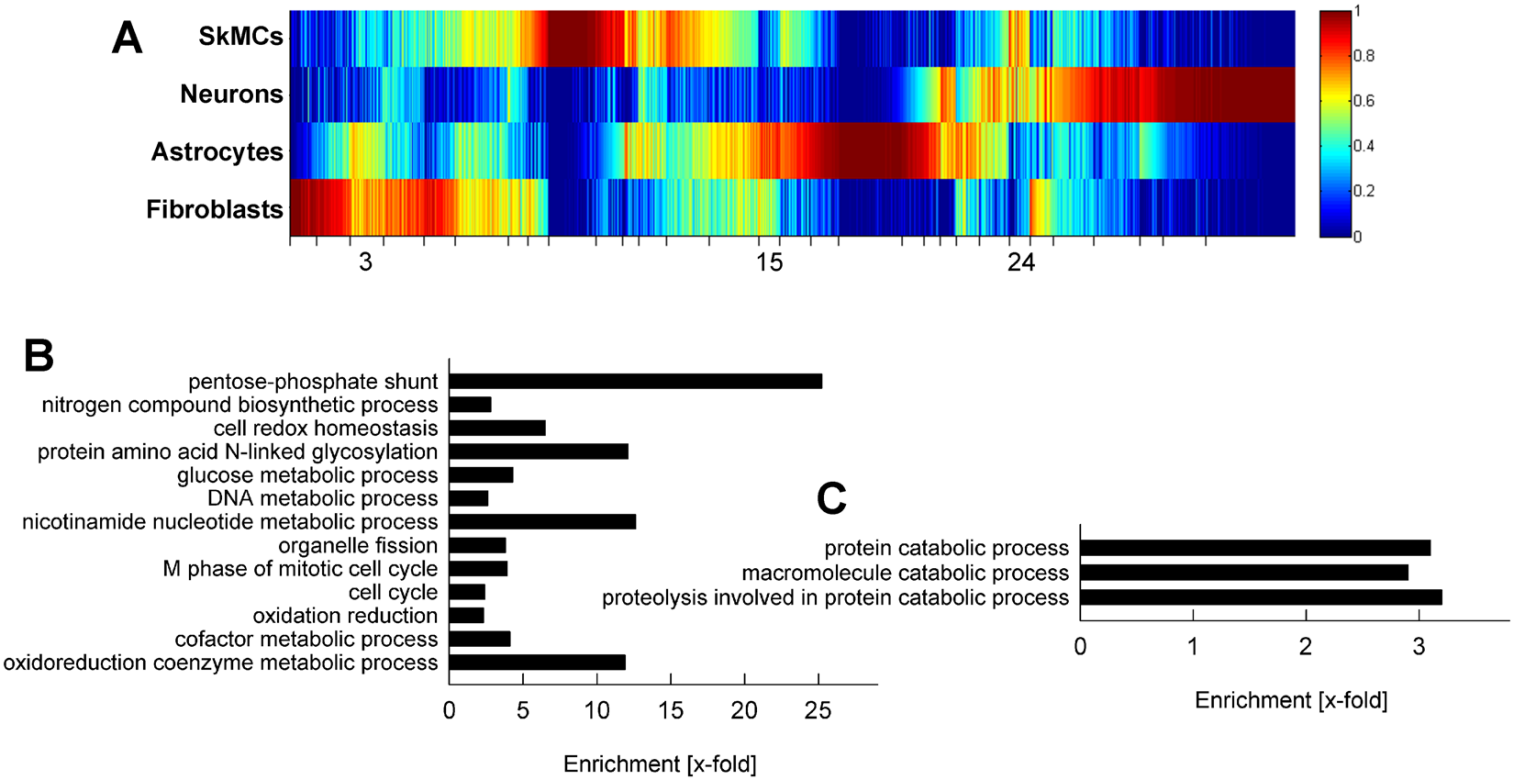
Differentially expressed genes in non-infected SkMCs, neurons, astrocytes and fibroblasts. Genome-wide expression profiles were obtained by RNA high-throughput sequencing. Genes that were differentially expressed between cell types were extracted (Chi^2^ test, FWER < 0.01). (**A**) Heat map of DEGs from SkMCs, neurons, astrocytes and fibroblasts. Expression profiles were clustered according to a one-dimensional self-organizing map. Gene clusters that were differentially expressed in SkMCs and neurons as compared to astrocytes and fibroblasts are indicated by cluster numbers. (**B**,**C**) Biological processes showing differential gene expression profiles between SkMCs and neurons as compared to astrocytes and fibroblasts. Annotation terms that were overrepresented among the genes from cluster 3 (**B)** and cluster 24 **(C**) as compared to the reference genome were identified by GO analysis (FDR < 0.05, modified Fisher exact test with Benjamini-Hochberg correction for multiple testing). Redundant annotation terms were omitted.

Mammalian cell types differ in their impact on *T. gondii* phenotypes *in vitro* and *in vivo*; whereas neurons and muscle cells spontaneously sustain bradyzoite formation and persistence of tissue cysts, astrocytes and fibroblasts do not support parasite stage differentiation unless exogenous stressors are applied. Since SkMCs, neurons, astrocytes and fibroblasts transcriptionally responded to *T. gondii* infection in a predominantly cell type-specific manner, we reasoned that signatures of non-infected cells may provide clues to host regulators of parasite stage conversion. Filtering of the sequencing data sets from non-infected SkMCs, neurons, astrocytes and fibroblasts identified 11,247 genes that were differentially expressed between cell types (Chi^2^ test, FWER < 0.01; Fig. 4A). Expression profiles of SkMCs and fibroblasts clustered again more closely together than those of the other two cell types and that of neurons was again the most distant one (see Supplementary Fig. S3). This indicates that the overall host cell expression profiles do not correlate with phenotypic differences in *T. gondii* after infection of SkMCs and neurons versus astrocytes and fibroblasts. However, out of the more than 11,200 DEGs, 3 gene clusters (No. 3, 15 and 24; Fig. 4A) comprising a total of 843 genes were identified showing similar expression profiles in SkMCs and neurons as compared to astrocytes and fibroblasts (for a complete list of genes within clusters 3, 15 and 24 see Supplementary Tables S6, S7 and S8, respectively). Expression of genes within clusters 3 and 15 was higher in astrocytes and fibroblasts than in SkMCs and neurons whereas genes within cluster 24 were more strongly expressed in SkMCs and neurons as compared to the other two cell types (Fig. 4A; also see Supplementary Tables S6–S8). Interestingly, 29 cell cycle-associated gene products were identified within cluster 3, mRNAs of which were more abundant in astrocytes and fibroblasts as compared to SkMCs and neurons (Fig. 4B, Table 1 and Supplementary Table S6). This is remarkable since we have recently shown that cell cycle-arrested myotubes but not proliferating myoblasts or fibroblasts trigger bradyzoite formation in *T. gondii* ^16^. Indeed, gene products which promote cell cycle progression including E2F1, Pttg1/securin (G1/S-transition) and Tipin (S-phase), as well as regulators of mitosis and cytokinesis (e.g. Ckap2, Dsn1, Mki67, Pttg1/securing, Smc2, Nusap1, Zw10) were up-regulated in astrocytes and fibroblasts as compared to SkMCs and neurons (for a complete list see Table 1). Transcript levels of Wee1, an inhibitor of mitosis entry were however also increased in astrocytes and fibroblasts. Another group of genes being enriched in cluster 3 encode enzymes of the pentose-phosphate shunt (Pgls, H6pd, G6pdx, Pgd, Taldo1, Pgm1) (Fig. 4B, Table 1; 25.2-fold enrichment). This suggests a higher glucose flux through the pentose-phosphate shunt in astrocytes and fibroblasts and may reflect their increased demand for nucleic acids and/or NADPH. NADPH is a major source for reducing equivalents which are required for anabolic reactions such as lipid or cholesterol biosynthesis and for protection against reactive oxygen species (ROS). Gene products that are associated with oxidation-reduction reactions, oxidoreduction coenzyme metabolism and nicotinamide nucleotide metabolism were consequently enriched in cluster 3 and thus up-regulated in astrocytes and fibroblasts as compared to SkMCs and neurons (Fig. 4B, Table 1 and Supplementary Table S6). Differences in protection against ROS between the two groups of cells were also indicated by increased mRNA levels of genes involved in cell redox homeostasis, (e.g. *Glrx*, *Prdx1*, *Gsr*, *Txnrd 1* and *2*; Fig. 4B, Table 1). Other processes, of which genes were enriched in cluster 3, were DNA metabolism, nitrogen compound biosynthetic process and N-linked protein glycosylation (Fig. 4B). Cluster 15 comprised 240 genes which showed increased mRNA levels in astrocytes and fibroblasts than in SkMCs and neurons (Fig. 4A; see also Supplementary Table S7). However, no biological process was identified with significantly enriched annotation terms (FDR > 0.05) associated with the gene list. This hampered to draw conclusions on the putative functions of these genes in regulating the interaction with *T. gondii*, and these genes are therefore not discussed in further detail. Expression of genes within cluster 24 was higher in SkMCs and neurons than in astrocytes and fibroblasts. Gene products that are associated with catabolic processes of macromolecules and particularly proteins, e.g. genes encoding components of the ubiquitin-proteasome system (*Ubac1*, *Ubr3*, *Usp13*, *Usp20*, *Ube2d1*) were significantly enriched within this gene list (Fig. 4C, Table 1 and Supplementary Table S8). Together, analysis of the transcriptomes of non-infected SkMCs, neurons, astrocytes and fibroblasts identified the host cell cycle, the pentose phosphate pathway, the cellular redox homeostasis and protein catabolism as putative regulators of *T. gondii* stage conversion.

**Table 1:** Expression of biological processes-associated host cell genes that are differentially enriched in the transcriptomes of SkMCs and neurons versus astrocytes and fibroblasts.

Gene ID	Description	No^a^	SkMCs^b^	Neurons	Astrocytes	Fibroblasts
**Cell cycle**						
Cd2ap	CD2-associated protein	3	5.30	2.28	9.71	12.20
Dclre1a	DNA cross-link repair 1 A	3	2.95	1.57	3.77	4.60
Dsn1	DSN1	3	1.82	1.02	4.50	5.11
E2f1	E2F transcription factor 1	3	8.06	4.80	11.83	14.83
Hjurp	RIKEN cDNA 6430706D22 gene	3	4.08	6.46	8.60	9.18
6720463M24Rik	RIKEN cDNA 6720463M24 gene	3	1.13	1.07	2.63	3.97
Rbm7	RNA binding motif protein 7	3	15.02	10.74	26.98	28.40
Wee1	WEE 1 homolog 1	3	4.62	1.63	7.65	7.23
Zw10	ZW10 homolog (Drosophila)	3	5.17	2.61	8.61	8.56
Mki67	Antigen identified by mAb Ki 67	3	2.65	11.52	14.51	17.44
Aspm	Abnormal spindle-like	3	0.49	1.55	3.03	3.30
AY074887	cDNA sequence AY074887	3	1.06	0.22	1.43	2.30
Csrp2bp	Cysteine and glycine-rich protein 2 binding protein	3	5.60	4.88	9.37	8.64
Ckap2	Cytoskeleton associated protein 2	3	2.21	3.99	19.48	22.86
Ncapd2	Non-SMC condensing I complex, D2	3	3.27	13.85	17.88	24.11
Ncapg2	Non-SMC condensing I complex, G2	3	1.53	1.29	7.48	7.82
Nde1	Nuclear distribution gene E homolog 1	3	15.60	4.99	29.43	28.57
Nfatc1	Nf of activated T cells, calcineurin-dependent 1	3	3.17	1.06	6.52	7.83
Nusap1	Nucleolar and spindle associated protein 1	3	1.25	2.54	6.32	8.19
Pttg1	Pituitary tumor-transforming gene 1	3	2.51	1.99	10.27	10.02
Fam33a	Predicted gene 6597	3	3.88	3.85	7.13	10.09
Ppp1cc	Protein phosphatase 1, catalytic subunit, gamma isoform	3	38.74	27.31	51.92	66.08
Pml	Promyelocytic leukemia	3	5.27	2.84	10.22	10.42
Psm3ip	Proteasome 26 S subunit, ATPase 3, interacting protein	3	2.48	1.49	5.39	6.51
Rcbtb1	RCC1 and BTB domain-containing protein 1	3	4.66	3.19	7.32	8.93
Rbl1	Retinoblastoma-like 1 (p107)	3	1.83	0.93	4.42	4.69
Gas2l3	Growth arrest-specific 2 like 3	3	0.70	0.68	2.72	3.59
Smc2	Structural maintenance of chr. 2	3	2.95	3.93	8.74	11.92
Tipin	Timeless interacting protein	3	6.50	5.64	16.65	23.84
**Pentose-phosphate shunt**						
Pgls	6-Phosphogluconolactonase	3	9.98	7.75	11.99	15.32
H6pd	Hexose-6-P dehydrogenase	3	7.85	1.08	10.27	11.26
G6pdx	Glucose-6-P-dehydrogenase X-linked	3	3.67	6.79	35.46	37.33
Pgd	Phosphogluconate dehydrogenase	3	19.22	10.86	43.79	41.67
Taldo1	Transaldolase 1	3	49.87	26.78	78.08	75.17
Pgm1	Phosphoglucomutase 1	3	7.90	3.05	13.02	12.78
**Cell redox homeostasis**						
Glrx	Glutaredoxin	3	19.04	14.91	54.32	49.24
Prdx1	Peroxiredoxin 1	3	89.64	17.77	229.96	252.73
Pdia3	PDI associated 3	3	121.25	99.01	183.22	244.69
Gsr	Glutathione reductase	3	6.81	5.30	15.96	20.74
Txndc12	Thioredoxin domain containing 12	3	11.18	19.46	31.20	35.48
Txnrd1	Thioredoxin reductase 1	3	35.78	28.50	64.64	74.06
Txnrd2	Thioredoxin reductase 2	3	3.34	2.00	5.99	6.78
Tmx1	Thioredoxin-related transmembrane protein 1	3	14.81	7.24	25.07	24.31
**Protein catabolic process**						
Fbxl2	F-box & leucine-rich repeat protein 2	24	9.04	5.64	3.19	1.94
Hectd3	Hect domain containing 3	24	15.24	15.79	10.10	7.86
Hecw2	HECT, C2 & WW domain containing E3 ubiquitin protein ligase 2	24	3.78	3.58	1.03	0.03
Otud7a	OUT domain containing 7 A	24	4.80	3.93	0.16	0.01
Wsb2	WD repeat & SOCS box-containing 2	24	41.64	46.61	20.88	14.32
Asb1	Ankyrin repeat & SOCS box-containing 1	24	7.79	4.79	3.52	2.65
Gan	Giant axonal neuropathy	24	5.91	4.27	1.18	1.10
Herc1	Hect domain & RCC1-like domain 1	24	4.69	5.81	3.38	1.87
Map1lc3a	Microtubule-associated protein 1 light chain 3 alpha	24	202.17	148.70	30.50	21.74
Kcmf1	Potassium channel modulatory factor 1	24	78.18	91.09	38.81	48.25
Rnf20	Ring finger protein 20	24	15.79	14.38	10.59	7.34
Rabgef1	RAB guanine nucleotide exchange factor 1	24	11.98	8.37	5.38	5.16
Socs2	Suppressor of cytokine signaling 2	24	6.85	3.83	0.58	0.40
Socs7	Suppressor of cytokine signaling 7	24	13.29	20.68	8.92	3.87
Ubac1	Ubiquitin associated domain containing 1	24	35.05	18.51	14.57	13.92
Ubr3	Ubiquitin protein ligase E3 component n-recognin 3	24	23.31	19.54	7.78	8.68
Usp13	Ubiquitin specific peptidase 13	24	8.31	4.73	0.84	0.61
Usp20	Ubiquitin specific peptidase 20	24	13.78	8.47	5.57	4.40
Ube2d1	Ubiquitin-conjugating enzyme E2D	24	25.80	38.85	4.95	6.80

^a^Cluster number.

^b^Data represent RPKM values.

### Expression profiles in *T. gondii* differ depending on the infected host cell type

**Figure 5:**
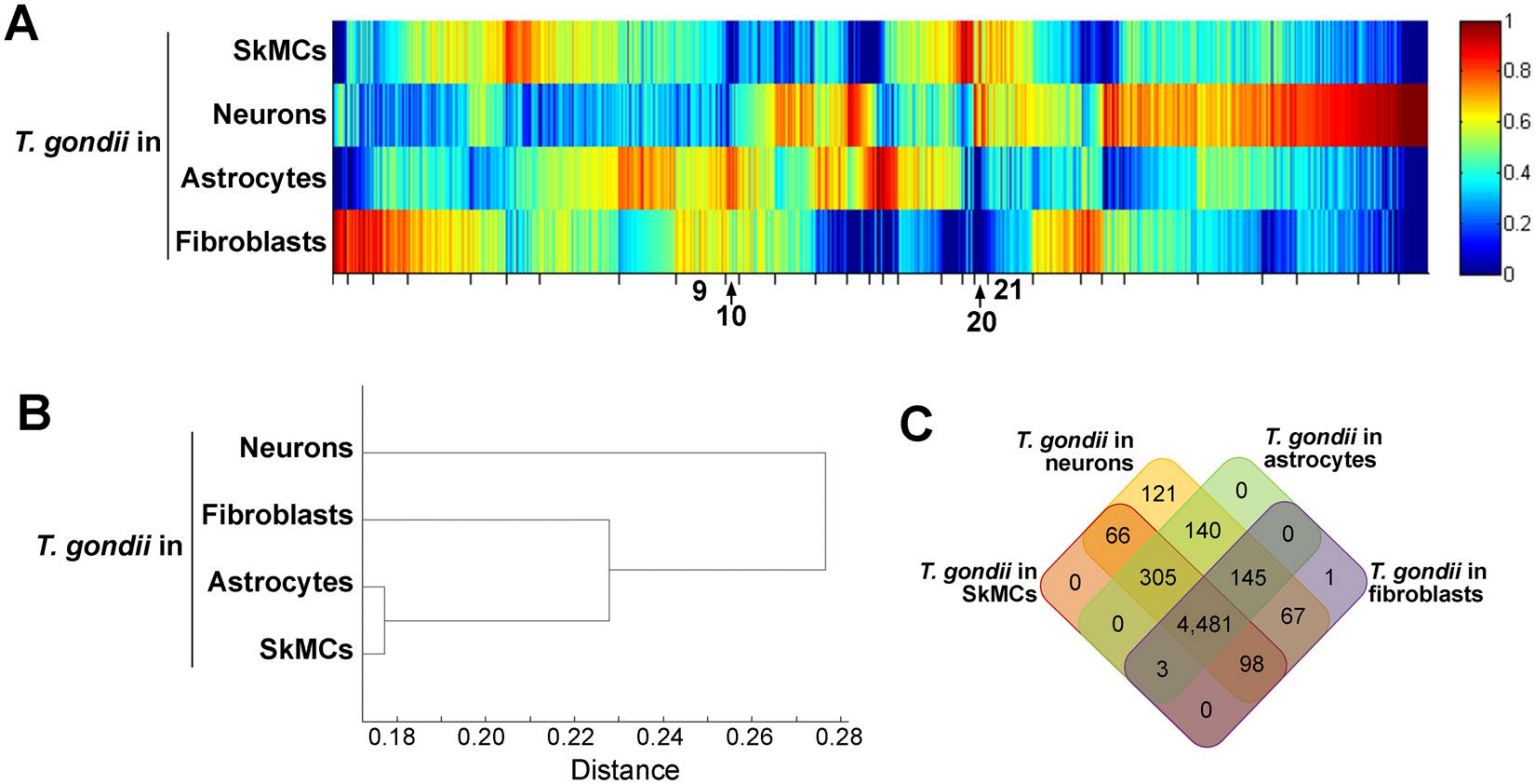
Heterogeneous gene expression in *T. gondii* after infection of mature SkMCs, neurons, astrocytes and fibroblasts at a MOI of 5:1 for 24 hours. Pools of total RNA from four different biological replicates each were used to prepare cDNA libraries which were then sequenced using Illumina technology. Reads were mapped to the *T. gondii* reference genome. Genes were identified the expression of which was differentially regulated depending on the host cell type (Chi^2^ FWER < 0.01). (**A**) Heat map of *T. gondii* DEGs after infection of SkMCs, neurons, astrocytes and fibroblasts. Expression profiles were clustered according to a one-dimensional self-organizing map. Clusters of genes that were differentially expressed after infection of SkMCs and neurons as compared to astrocytes and fibroblasts are indicated by cluster numbers. (**B**) Hierarchical cluster analysis of genes differentially expressed after infection of the different host cell types. (**C**) Venn diagram of genes differentially expressed in *T. gondii* after infection of the different host cell types.

In order to further characterize the interaction of *T. gondii* with the different types of host cells, we identified within samples from infected cells between 0.37 and 2.05% of the total reads mapping to the parasite reference genome (see Supplementary Table S1). In contrast, only background numbers of reads within samples from non-infected cells mapped to the parasite genome as expected. It is important to note that we did not isolate the parasites prior to sample preparation in order to rapidly isolate RNA from parasites within their physiological niche. This explains the relatively low proportion of reads mapping to the *T. gondii* genome. Data sets were then filtered for genes with at least 5 total reads in at least one of the samples in order to account for the relatively low read numbers that are inherent to pathogen analyses in host-pathogen co-transcriptomes. Using a FWER < 0.01 (moderated Chi^2^ test), a total of 5,427 genes were identified that were differentially expressed in *T. gondii* after infection of SkMCs, neurons, astrocytes or fibroblasts. Largely heterogeneous expression profiles of *T. gondii* within the different cell types were confirmed by heat map visualization (Fig. 5A). Hierarchical cluster analysis indicated that the DEG profiles of *T. gondii* within astrocytes and SkMCs were more similar than those within the other two cell types (Fig. 5B). Furthermore, the *T. gondii* transcriptome from infected neurons was the most divergent one as compared to the other three infected cell types. Despite the regulation of gene expression in *T. gondii* in a host cell type-dependent manner, Venn analysis of the expression pattern revealed that 4,481 of the 5,427 DEGs were commonly expressed after infection of all four cell types (Fig. 5C). This indicates quantitative differences in the expression levels of most of the parasite DEGs rather than different sets of genes being expressed or silenced after infection of different host cell types by *T. gondii*. However, we also identified a variety of genes that were expressed in *T. gondii* in a host cell type-specific manner (Fig. 5C). Remarkably, 121 genes were exclusively expressed by *T. gondii* after infection of neurons and several hundred genes were specifically expressed in neurons and one or two other but not all four cell types (Fig. 5C). In contrast, only a single gene was exclusively expressed when *T. gondii* had infected a fibroblast and no genes were identified that were exclusively expressed by *T. gondii* in either SkMCs or astrocytes. This highlights the divergence in gene expression when *T. gondii* resides within a neuron as compared to gene expression in SkMCs, astrocytes or fibroblasts.

**Figure 6:**
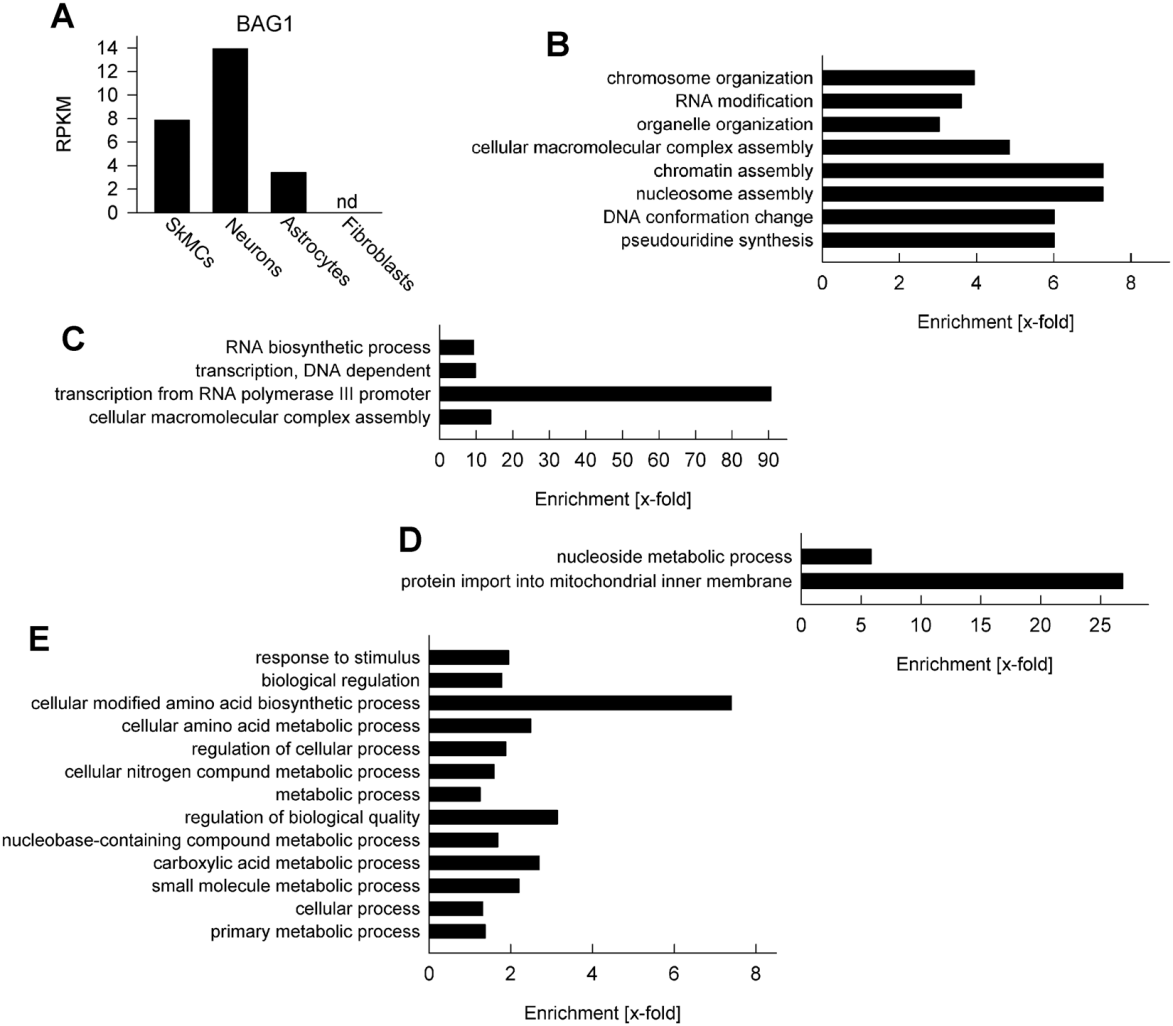
Differential gene expression in *T. gondii* following infection of either mature SkMCs and neurons or astrocytes and fibroblasts at a MOI of 5:1 for 24 hours. Expression profiles of *T. gondii* were obtained by high-throughput RNA sequencing and parasite genes were extracted that were differentially expressed depending on the host cell type (Chi^2^ FWER < 0.01). (**A**) Expression of bradyzoite antigen (BAG)-1 in *T. gondii* during infection of host cells as indicated. Data are reads per kilobase per million mapped reads (RPKM). Nd: not detected. (**B**–**E**) Biological processes showing differential gene expression profiles in *T. gondii* residing within SkMCs and neurons as compared to those in astrocytes and fibroblasts. Annotation terms that were overrepresented among the genes from cluster 9 (**B**), cluster 10 (**C)**, cluster 20 (**D**) and cluster 21 (**E**) indicated in Fig. 5A as compared to the reference genome were identified by using the GO analysis tool at www.toxodb.org (FDR < 0.05 with Benjamini-Hochberg correction for multiple testing). Redundant annotation terms were omitted.

Next, we sought identifying common signatures in parasite gene expression in SkMCs and neurons opposed to astrocytes and fibroblasts. We confirmed that *T. gondii* expressed bradyzoite antigen 1 (BAG1) mRNA more abundantly in neurons and SkMCs as compared to astrocytes and fibroblasts (Fig. 6A). Since BAG1 is an early and abundant marker for bradyzoite development^21^ this suggested that neurons and SkMCs indeed sustain stage conversion more readily than astrocytes and fibroblasts. Clusters of genes were then identified that were either more strongly expressed in astrocytes and fibroblasts as compared to SkMCs and neurons (clusters 9 and 10 in Fig. 5A; see also Supplementary Table S10) or *vice versa* (clusters 20 and 21 in Fig. 5A; see also Supplementary Table S9). Among the 249 genes present in cluster 9, expression of histone genes including *H2ax*, *H4* and *H2a1* was clearly higher in *T. gondii* residing in astrocytes and fibroblasts as compared to those in neurons and muscle cells (see Supplementary Table S10). In addition, we identified mRNAs of cell cycle regulators (cyclin-dependent kinase, putative cell cycle-associated protein kinase) and several members of the AP2 transcription factor family (AP2X-3, AP2XII-6, AP2VIII-4, AP2XI-1) being more abundant in *T. gondii* in astrocytes and fibroblasts than in neurons and SkMCs (see Supplementary Table S10). This is remarkable since the parasite cell cycle and expression of AP2IX-9 and AP2XI-4 are known to regulate differentiation of *T. gondii* ^9, 22, 23^. Consistent with these differentially expressed genes^24^, GO terms related to chromatin biology and chromosome organization were enriched among the genes of cluster 9 (Fig. 6B). Furthermore, RNA modification and pseudouridine synthesis appear to differ in *T. gondii* depending on the host cell environment. Expression of the 63 genes present in cluster 10 was also higher in *T. gondii* in astrocytes and fibroblasts than in neurons and SkMCs (see Supplementary Table S10). Among the most differently expressed ones of this cluster were again two AP2 members, namely AP2X-10 and AP2VII-A1. GO term analysis revealed enrichment of genes related to RNA synthesis and macromolecular complex biogenesis (Fig. 6C). Cluster 20 comprises 71 genes, expression of which was increased in *T. gondii* after infection of SkMCs and neurons as compared to astrocytes and fibroblasts (see Supplementary Table S9). Three of these genes encode SRS (SAG-related sequence) proteins, namely SRS49B, SRS23 and SRS16E, which are all up-regulated during chronic mouse infection *in vivo* or upregulated during bradyzoite formation *in vitro* (www.toxodb.org). ‘Nucleoside metabolic process’ and ‘protein import into mitochondrial inner membrane’ were GO process terms also associated with this gene cluster (Fig. 6D). Finally, cluster 21 comprises 217 genes that were also more strongly expressed when *T. gondii* had infected neurons or SkMCs than after infection of astrocytes or fibroblasts (see Supplementary Table S9). Among them, the DnaK-tetratricopeptide repeat (*DnaK-TPR*) gene was identified which has been shown to be up-regulated in bradyzoites^25, 26^. Furthermore, genes encoding chromatin-modifying proteins (HDAC2, Chromo domain-containing protein and MYSTA), redox homeostasis-regulating proteins (glutaredoxin domain-containing proteins, thioredoxin family Trp26 protein) and again an AP2 transcription factor (AP2XII-4) were among these genes (see Supplementary Table S9). GO term analyses identified genes involved in different metabolic processes including primary metabolism, amino acid metabolism, nitrogen/nucleobase compound metabolism and carboxylic acid metabolism as well as in ‘response to stimulus’ to be enriched within this cluster (Fig. 6E). It indicates that SKMCs and neurons affect the parasites metabolism differently than astrocytes or fibroblasts and extends previous reports on various metabolic differences between tachyzoites and bradyzoites^27^. Within the four gene clusters, ~50% of DEGs were annotated as hypothetical (see Supplementary Tables S9, S10). Together, these data indicate that depending on the host cell type environment, *T. gondii* gene expression differs largely quantitatively but may also differ qualitatively in distinct cell types as shown here for neurons. Common gene expression signatures including those related to the parasites cell cycle, its chromatin biology, its metabolism and AP2-regulated transcription were however identified that might favour or prevent stage conversion within neurons and SkMCs or within astrocytes and fibroblasts, respectively.

## Discussion

Fundamental to infection biology is the question of how a host and its invader respond to infection. Here we uncovered largely heterogeneous, host cell type-specific expression profiles in both the host cells and the pathogen during intracellular infection. SkMCs, neurons, astrocytes and fibroblasts differed significantly in the expression of more than 16,000 genes and infection with *T. gondii* had only a minor impact on these transcriptomes. Intriguingly, SkMCs, neurons, astrocytes and fibroblasts up- or down-regulated the expression of largely different sets of genes after *T. gondii* infection. Likewise, parasites residing within the different host cell types presented transcriptomes which differed in expression levels of more than 5,000 genes. These results have important implications for the host-pathogen interaction during toxoplasmosis and possibly other infectious diseases as well.

First, *T. gondii* may encounter largely heterogeneous cellular microenvironments after infection of different host cell types. We have identified more than 11,200 genes that were differentially expressed in SkMCs, neurons, fibroblasts and astrocytes before infection. SkMCs and fibroblasts originate from the mesoderm whereas neurons and astrocytes are ectoderm-derived. Comparative transcriptomics revealed that SkMCs and fibroblasts indeed clustered more closely together as compared to the other cell types whereas this was not the case for astrocytes and neurons. Due to the parasite-driven active invasion process of *T. gondii* ^5^ and its promiscuous infection of a wide range of nucleated cells^28^, these cell types may also get infected by *T. gondii in vivo*. Within these cells, the parasite resides inside a parasitophorous vacuole that is permeable to molecules of up to 1300 Da^29^. Therefore, only a subset of the differentially expressed genes within SkMCs, neurons, fibroblasts and astrocytes may translate into environmental cues to which the parasite is directly exposed to. After invasion of such different host cell types as used herein the parasite will nevertheless experience rather distinct ecological niches. The course of infection is therefore likely governed by the types of host cells *T. gondii* infects.

Second, different host cell types respond with the up- or downregulation of largely different sets of genes to infection with *T. gondii*. Until now, genome-wide transcriptional profiles of mammalian cells after infection with *T. gondii* have been mainly characterized using a single cell type, mostly fibroblasts or macrophages^20, 30–32^ or two closely related cell types, i.e. dendritic cells (DCs) and macrophages^33^. Here, we have conducted the first direct comparison of the transcriptomes of four developmentally and functionally heterogeneous cell types after infection. The fact that only a few genes were commonly regulated in different host cell types is intriguing but is reminiscent to previous findings that even two myeloid cell types, i.e. DCs and macrophages strongly differ in their responses to intracellular infection^33^. Less surprisingly, in those cells genes involved in inflammation and immunity were prominently regulated^33^. In SkMCs, neurons, fibroblasts and astrocytes only a few immune response-related genes were regulated after infection with *T. gondii*. In human fibroblasts, such genes were mainly up-regulated during the first 2 hours of infection although increased expression of several of them also persisted until 24 hours of infection^20^. Since we have analyzed the transcriptome of multiple cell types at a single time point, i.e. at 24 hours of infection, *T. gondii* may likely regulate additional genes at other time points during infection. Furthermore, differences in expression profiles between this study and those of others^20, 33^ can also be explained by using cells from different hosts, i.e. humans and mice. Due to the differences between the responses of distinct cell types as observed herein we can indeed anticipate also significantly divergent host species-dependent expression profiles after infection with *T. gondii*. Despite such differences we also recognized similarities in the parasite-regulated expression profiles described herein and those previously published. Genes involved in translation, for instance, were consistently up-regulated in fibroblasts and astrocytes (Fig. 3) and human fibroblasts^20^ at 24 hours of *T. gondii* infection. Similarly, regulation of the host cell cycle by *T*. *gondii* as recognized by Blader *et al*.^20^ was confirmed here to occur in SkMCs and to a lesser extent in neurons. Importantly, our finding that only a few genes were similarly regulated in multiple cells and none was regulated in all four cell types after infection with *T. gondii* strongly suggests that transcriptional changes of the host represent cell type-specific responses rather than parasite-driven manipulations of the host cell in order to facilitate its own intracellular survival. An alternative explanation, i.e. parasite-driven regulation of common host cell signalling cascades leading to different transcriptional programs in different host cell types is less likely and is not supported by our data. The differences in expression profiles that we uncovered here clearly exceed those previously recognized after infection of human DCs and macrophages exposed to distinct pathogens including *T. gondii* ^33^. In that study, DCs generally responded more diverse to infection than macrophages but several hundreds of genes were commonly regulated in both cell types in response to various pathogens albeit with largely different expression patterns. Here, SkMCs, neurons, astrocytes and fibroblasts transcriptionally also responded to infection with varying diversity, but remarkably, not a single gene was commonly up-regulated in all four cell types after infection. Thus, the more heterogeneous host cell types are, the more diverse they appear to regulate gene expression after infection. This finding challenges transferring data on the parasite-host interaction from one host cell type to another. It also indicates that infection of distinct host cell types may regulate the course of toxoplasmosis differently.

Third, *T. gondii* as well responds heterogeneously to its cellular environments. The majority of *T. gondii* DEGs (~83%) differed quantitatively between parasites within the different host cell types. After infection of neurons, we recognized however a substantial amount of genes that were either completely neuron-specifically expressed or whose expression was shared between neurons and one or two other cell types. Together, it indicates that *T. gondii* can sense various cellular microenvironments and translate this into a tunable transcriptional program. Furthermore, the parasite response to neurons is particularly diverse. Whether this is related to the predilection of *T. gondii* to form tissue cysts and to persist within neurons, needs future validation. Importantly, among the *T. gondii* DEGs we identified four clusters of genes expression of which correlated with the propensity of the parasite to differentiate to the bradyzoite stage within neurons and SkMCs but not fibroblasts and astrocytes. As a proof-of-concept, we identified markers of bradyzoite and/or cyst development including genes encoding distinct SRS surface proteins and the DnaK-TPR^25, 26^ to be more strongly expressed in neurons and SkMCs than in fibroblasts and astrocytes. It must be stressed that several other bradyzoite-specific transcripts (e.g. enolase 1, lactate dehydrogenase 2) were not identified among the *T. gondii* DEGs but this may be due to the isolation of RNA at 24 hours post infection, i.e. an early time point in the transition towards the bradyzoite stage. Detailed functional analyses of the gene clusters were hampered because (i) ~50% of the genes within these data sets encoded hypothetical proteins and (ii) standard GO term enrichment tools are not applicable to *T. gondii*. Despite these limitations, we nonetheless identified processes that might regulate *T. gondii* stage conversion. Genes involved in cell cycle progression, chromatin assembly, chromosome organization and RNA biosynthesis and those encoding AP2 transcription factors were more abundant in astrocytes and fibroblasts than in neurons and SkMCs. They are indicative for active replication^9^ and a high demand for RNA in the tachyzoite stage. In contrast, genes involved in metabolic processes including amino acid and nitrogen compound/nucleobase metabolism were more abundantly expressed by *T. gondii* within neurons and SkMCs. This is consistent with differences in the carbohydrate metabolism between tachyzoites and bradyzoites^27^ and suggests additional metabolic differences between both stages. Which of the identified genes indeed represent previously unknown positive and negative regulators of stage conversion in *T. gondii*, needs to be evaluated in the future.

Finally, among the more than 11,000 genes which were differentially expressed in SkMCs, neurons, astrocytes and fibroblasts before infection, signatures were identified that differed between SkMCs and neurons on the one hand and astrocytes and fibroblasts on the other. Among them, we noted genes involved in cell cycle progression, the pentose phosphate pathway, cell redox homeostasis and protein catabolism. They are of major interest because they represent putative host regulators of *T. gondii* stage differentiation. A permanent and irreversible withdrawal from the cell cycle indeed triggers bradyzoite formation in mature SkMCs in the absence of exogenous stress factors^16, 34^. In addition, expression of the negative cell cycle regulator cell division autoantigen (CDA)-1 triggers bradyzoite formation in human fibroblasts and epithelial cells^35^, and knock-down of its murine orthologue favours sustained replication of the tachyzoite stage in SkMCs^16^. We now noted that the predominant expression of cell cycle progressors in astrocytes and fibroblasts correlates with decreased levels of *T. gondii* BAG1 expression, i.e., a marker of bradyzoite differentiation^21, 36^. The negative correlation of host cell cycle progressors and *T. gondii* BAG1 expression again highlights the importance of the host cell cycle for intracellular parasite development. The pentose phosphate pathway, cell redox homeostasis and protein catabolism are other candidates that could regulate stage differentiation of *T. gondii*. Future validation is necessary to experimentally prove involvement of these host cell processes in the parasites’ intracellular development.

In conclusion, the interaction of *T. gondii* and its host cell is largely host cell type-specific and this affects the transcriptional responses of both parasite and host cell. The parasites ability to adapt to different host cells may have enabled *T. gondii* to also broaden its host range during evolution.

## Methods

### Isolation of cells, cell culture and cell differentiation

C57BL/6 mice were purchased from Harlan (Borchen, Germany) and were bred at the animal facility of the university clinics Magdeburg. Animal care and experimentation were performed according to European regulations and have been approved by the local authorities (Landesverwaltungsamt Halle, Germany). Neuronal cells were isolated from cortices of mouse embryos at day 18.5 of gestation as described^37^. After careful removal of the meninges, cortices were minced and gently dissociated by incubation in 0.25% trypsin in PBS (PAA Laboratories, Pasching, Austria) at 37 °C for 25 minutes. After centrifugation, residual trypsin within the pellet was inactivated by 0.52 g/l trypsin-inhibitor supplemented with 0.04 g/l DNase I and 3 g/l bovine serum albumin (all from Sigma, Taufkirchen, Germany). After having been washed in Hank’s balanced salt solution (PAA Laboratories) and after having been further dissociated mechanically, cells were resuspended in Neurobasal medium supplemented with 2% B27 (both from Invitrogen, Karlsruhe, Germany), 100 U/ml penicillin, 100 µg/ml streptomycin and 500 µM L-glutamine (all from PAA Laboratories). Cortical cells were seeded in poly-D-lysine-coated cell culture flasks or 24-well cell culture plates containing glass cover slips. Every 4 days, two-thirds of medium was changed. The purity of neurons was regularly ≥98% as determined by immunofluorescence microscopy.

Astrocytes were prepared from brains of C57BL/6 pups at day 0–1 after birth^38^. After careful removal of the meninges, brain tissue was mechanically dissociated using 70 µm nylon sieves (BD Biosciences, Heidelberg, Germany). Cells were propagated in 25-cm^2^ cell culture flasks in Dulbecco Modified Eagle Medium (DMEM) supplemented with 20% heat-inactivated fetal calf serum (FCS; both from PAA Laboratories), 2 mM each of L-alanin and L-glutamine (Biochrom AG, Berlin, Germany) and 20 µg/ml gentamicin (Sigma-Aldrich, Steinheim, Germany). After 5 days, medium was exchanged and FCS concentration was reduced to 10%. Four days later and at sub-confluency, contaminating microglia were detached by orbital shaking and astrocytes were isolated by trypsinization. After centrifugation, cells were again propagated in DMEM, 20% FCS medium (see above). Sub-confluent astrocytes were then isolated and seeded in DMEM, 10% FCS (as above). Control staining regularly revealed >80% of GFAP^+^ astrocytes within these cultures.

The murine skeletal muscle myoblast cell line C2C12 and NIH/3T3 fibroblasts were purchased from the European Collection of Animal Cell Cultures (ECACC) (Salisbury, UK). They were propagated in DMEM supplemented with 10% FCS, 100 U/ml penicillin and 100 µg/ml streptomycin (all reagents from Biochrom, Berlin, Germany). Myoblasts were seeded at 10 × 10^4^ per well in 6-well plates or at 2 × 10^4^ in 24-well plates containing glass cover slips. Twenty-four hours after seeding, myogenic differentiation of C2C12 myoblasts to syncytial myotubes was induced by incubation for 162 hours in DMEM supplemented with 2% heat-inactivated horse serum, 100 U/ml penicillin and 100 µg/ml streptomycin as described previously^16^.

### Parasite infection

Tachyzoites of the *T. gondii* type II strain NTE^39^ were propagated in murine L929 fibroblasts in Roswell Park Memorial Institute (RPMI) medium supplemented with 1% FCS, 100 U/ml penicillin, and 100 µg/ml streptomycin (all reagents from Biochrom). For infection of SkMCs, neurons, astrocytes and fibroblasts, parasites were isolated after onset of host cell lysis by differential centrifugation as described previously^40^. Briefly, contaminating host cells were pelleted by centrifugation at 34 × g for 5 minutes and parasites were subsequently isolated from the supernatant by centrifugation at 1300 × g for 10 minutes. Host cells were infected at a multiplicity of infection of 5:1. One day before infection, media of all host cell cultures were exchanged to DMEM, 10% FCS and penicillin/streptomycin (as above).

### RNA isolation, preparation of cDNA libraries and sequencing

Total RNA of *T. gondii*-infected and non-infected samples was isolated at 24 hours post infection using the GenElute Mammalian Total RNA Miniprep Isolation Kit (Sigma-Aldrich, Taufkirchen, Germany). The quality of each RNA preparation was assessed by running a Eukaryote Total RNA Nano assay on Agilent’s 2100 Bioanalyzer (Agilent Technology, Böblingen, Germany). For high throughput sequencing, RNAs from four biological replicates of each infected and non-infected cell type of high quality (RIN > 8.0) were pooled. cDNA libraries of each mRNA pool were prepared using the mRNA-Seq Sample Prep Kit (Illumina, San Diego, CA) according to the manufacturer’s protocol. Briefly, polyA-containing mRNA was purified using Sera-mag magnetic oligo(dT) beads and was then fragmented using microTUBEs (Convaris, Brighton and Hove, UK). First and second strand cDNAs were synthesized from the mRNA fragments using SuperScript II and DNA polymerase I, respectively, and mRNA was digested using RNaseH. cDNA overhangs were blunted using T4 and Klenow DNA polymerases, and 3′ A overhangs were added by Exo^-^ Klenow polymerase. PE adapters (Illumina) were then ligated to the cDNA fragments. After purification and agarose gel electrophoresis, DNA fragments of 200 ± 25 bp were excised and PCR amplified using adapter-specific primers. Libraries were again purified and validated on an Agilent 2100 Bioanalyzer.

High-quality libraries were immobilized on the flow cell of Illumina Genome Analyzer IIx or Illumina HiSeq. 2500. After solid-phase PCR amplification, single end 36 bp reads (SkMCs, astrocytes, fibroblasts) or single end 50 bp reads (neurons) of the library fragments were recorded. Reads were mapped to the *Mus musculus* reference genome (MGSCv37) and the *T. gondii* ME49 genome (version 7.0; www.toxodb.org) using CLC bio (Qiagen, Aarhus, Denmark).

### Data processing and differential gene expression analysis

Reads aligned to exons were transformed into relative expression values by calculating RPKM (reads per kilobase per million mapped reads) values for each gene. Differentially expressed genes (DEGs) were identified using a moderated Chi^2^ test as described^41^ comparing cell types or infected and non-infected samples. P-values were adjusted for multiple testing using Holm-Bonferroni familywise error rate (FWER) correction^42^. Log-transformed RPKM values of DEGs were used for hierarchical cluster analysis based on linear correlation (distance corresponds to 1 – Pearson coefficient) and average linkage method. Clustering and visualization of normalized gene expression profiles based on one-dimensional self-organizing maps were performed using the MarVis-Suite software^18^ and default settings. The lists of regulated genes were functionally analysed using the DAVID (database for annotation, visualization, and integrated discovery; http://david.abcc.ncifcrf.gov) bioinformatics resources as described^43^. Enrichment of GO terms of biological processes within each list of DEGs was identified by the functional annotation chart module using a threshold gene count of ≥2 and a FDR < 0.05 (modified Fisher exact test with Benjamini-Hochberg correction for multiple testing). Biological processes being overrepresented within sets of *T. gondii* genes were identified using the Gene Ontology Enrichment tool within the *Toxoplasma* Genomic Resource database, version 28 (www.toxodb.org). Sequence data has been deposited in NCBI’s Gene Expression Omnibus (GEO Series accession number GSE94595).

### Reverse transcription and qPCR

Total RNA from infected and non-infected mouse cells was isolated as indicated above and residual DNA was digested using DNase I (Sigma-Aldrich, Taufkirchen, Germany). Messanger RNA was reverse transcribed using the Omniscript RT kit (Qiagen, Hilden, Germany) and oligo(dT) primers. Serial dilutions of cDNA were then amplified in a LightCycler 1.5 using the SYBR Green I FastStart DNA Master^Plus^ Kit (Roche Diagnostics, Mannheim, Germany) and primer pairs as specified in Supplementary Table S11. The relative expression of mouse target genes was determined according to the ΔΔCt method and was normalized to β-actin as reference gene using the formula: Ratio_infected vs. non-infected_ = E_target_^ΔCP target (non-infected - infected)^ /E_reference_^ΔCP reference (non-infected - infected)^. Preliminary data indicated that β-actin was similarly expressed in the different host cell types before and after infection.

### Immunofluorescence staining and confocal microscopy

SkMCs, neurons, astrocytes and fibroblasts and infection of host cells with *T. gondii* were immunocytochemically assessed. To this end, cells grown on glass cover slips were fixed at 24 hours after infection using 4% paraformaldehyde in 0.1M sodium cacodylate (pH 7.4). After having been washed, cells were quenched for 10 min in 50 mM NH_4_Cl in PBS. They were then permeabilized and unspecific binding sites were blocked overnight in 0.1 mg/ml saponin, 1% bovine serum albumin in PBS at 4 °C. Mature syncytial SkMCs, i.e., myotubes and neurons were labelled during 1 hour at room temperature with rabbit polyclonal anti-myosin heavy chain (MyHC; H-300, Santa Cruz Biotechnology, Heidelberg, Germany) and mouse anti-neuronal class-III β-tubulin (Covance, Princeton, NJ) at dilutions of 1:100 and 1:400, respectively. Astrocytes or fibroblasts were incubated with rat anti-glial fibrillary acidic protein (GFAP; Zymed, San Francisco, CA) and mouse anti-actin (clone C4, kindly provided by J. Lessard, Cincinnati, OH) at dilutions of 1:500 and 1:250, respectively. Parasite-specific proteins were simultaneously labelled using rabbit or mouse polyclonal anti- *T. gondii* antiserum depending on the host cell type-specific primary antibody (both at a dilution of 1:100). After having been washed, host cells were stained using Cy2-conjugated donkey F(ab’)_2_ fragment anti-rabbit, anti-mouse or anti-rat IgG. Parasite-bound antibodies were simultaneously labelled using Cy5-conjugated donkey F(ab’)_2_ fragment anti-rabbit or anti-mouse IgG (all secondary antibodies from Dianova, Hamburg, Germany). The total cell population was visualized by incubation for 5 min in 5 µg/ml propidium iodide in PBS. Cells were mounted with Mowiol 4–88 (Calbiochem-Novabiochem, Bad Soden, Germany) and cells were analysed using a Leica TCS SP2 confocal microscope.

### Data Availability

Sequence data has been deposited in NCBI’s Gene Expression Omnibus (GEO Series accession number GSE94595).

## Electronic supplementary material

Supplementary Information
