# Supplementary material for: Divergent co-transcriptomes of different host cells infected with Toxoplasma gondii reveal cell type-specific host-parasite interactions
Source: Sci Rep. 2017 Aug 3;7:7229. doi: 10.1038/s41598-017-07838-w (PMC5543063; doi:10.1038/s41598-017-07838-w)
Supplement: Supplementary file 1 — Supplementary Information [file 41598_2017_7838_MOESM1_ESM.pdf]

Supplementary Information

Supplementary Figure S1

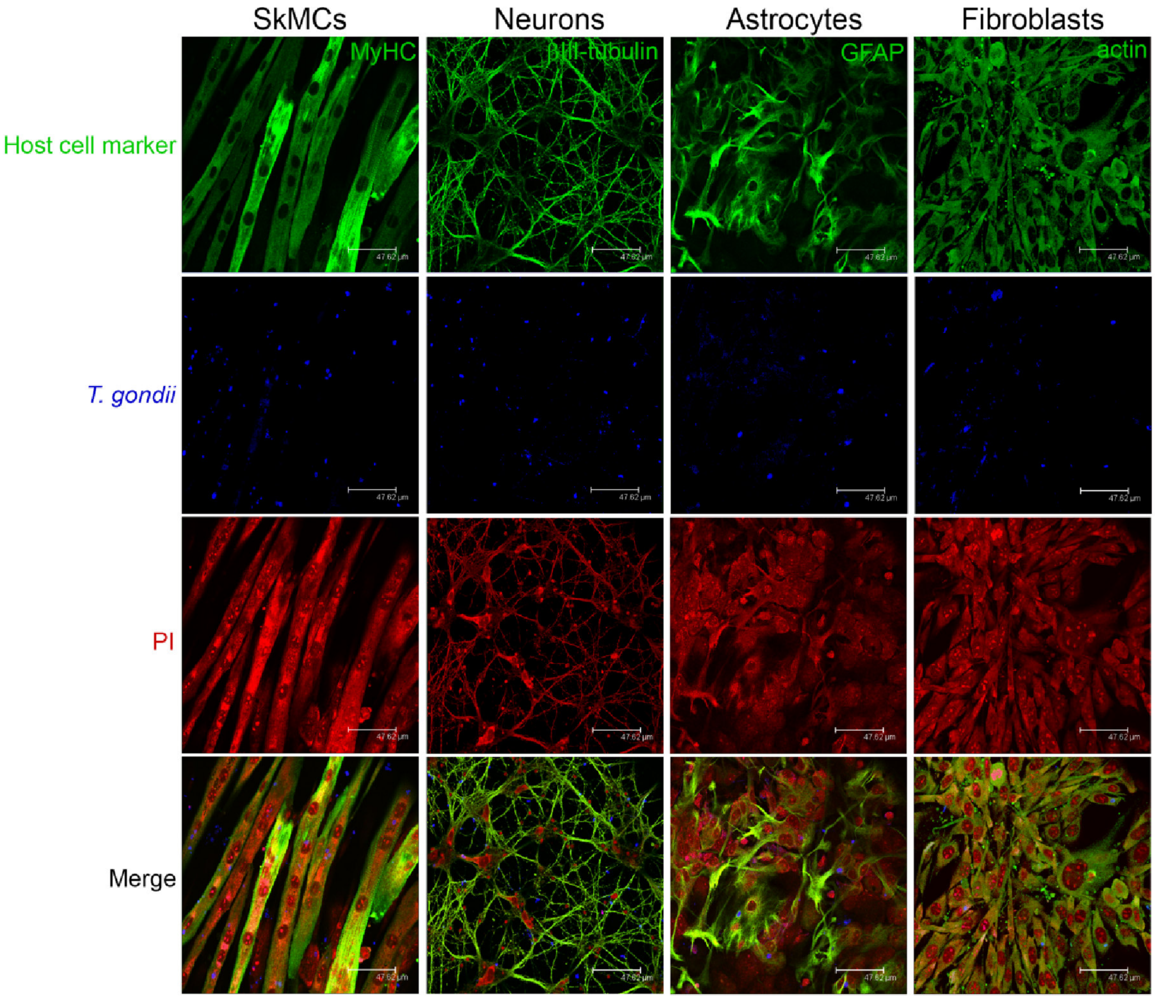

Control staining of SkMCs, neurons, astrocytes and fibroblasts after infection with *T. gondii*. Mature C2C12 myotubes, cortical neurons and astrocytes, and NIH/3T3 fibroblasts were infected with *T. gondii* at a MOI of 5:1 for 24 hours. After fixation, host cells were stained with myosin heavy chain (MyHC)-, neuronal class-III β-tubulin-, GFAP- or actin-specific primary and Cy2-conjugated secondary antibodies (green fluorescence), and parasites were stained with anti-*T. gondii* primary and Cy5-conjugated secondary antibodies (blue fluorescence). Total cell populations were visualized using propidium iodide (red fluorescence). Representative images from each labelling were acquired by confocal laser scanning microscopy and were superimposed.

Supplementary Figure S2

A

Gene ID	SkMCs		Fibroblasts		Neurons		Astrocytes	
	RT-qPCR	RNAseq	RT-qPCR	RNAseq	RT-qPCR	RNAseq	RT-qPCR	RNAseq
S100a4	1.00[a]	1.03[a]	1.12	0.98	14.25	13.29	0.94	0.89
Egr2	5.43	7.79	1.85	1.17	0.94	1.04	1.28	1.29
Cxcl10	5.22	n.a.[b]	2.44	0.28	6.62	8.29	3.24	3.00
Dpm1	0.93	0.86	0.94	1.14	0.88	1.12	1.00	0.36
Fosb	12.41	18.06	0.80	1.03	0.81	2.56	1.52	1.50
Ptgs2	6.20	7.56	1.14	0.81	1.95	1.20	1.43	1.43
Slc44a4	0.27	0.47	n.a.	n.a.	n.a.	0.54	n.a.	n.a.
Ttpal	1.45	2.76	1.03	6.50	0.85	1.12	2.90	1.56
Serinc4	0.98	2.42	0.52	0.28	0.60	1.83	1.12	0.55
Palm2	0.76	0.95	1.15	0.45	0.84	0.99	1.37	1.23
Nr4a1	4.55	6.00	0.76	1.06	0.90	1.19	1.39	1.24
Ubp1	0.78	1.00	0.91	0.47	0.74	1.07	2.39	0.86
Cyr61	2.25	2.95	1.02	1.14	1.19	1.09	1.00	0.94
Smyd1	0.78	0.85	1.32	3.39	1.11	1.36	0.77	0.90

[a]data are x-fold changes of mRNA by *T. gondii* infection

[b]not applicable

B

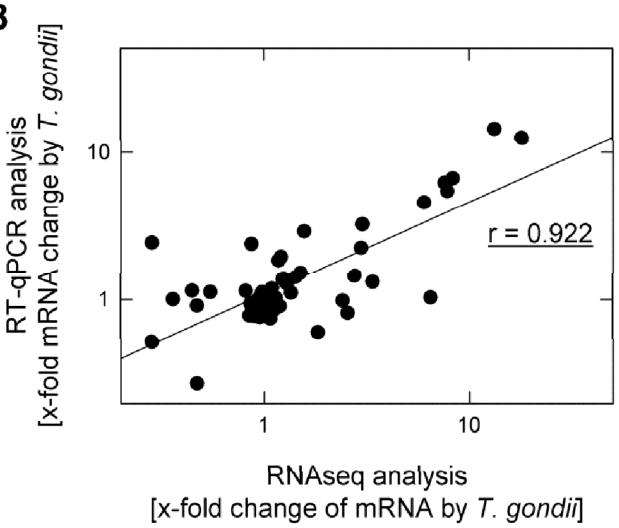

Validation of RNA sequencing data using quantitative RT-PCR. Fourteen representative genes were selected that showed different levels of regulation after infection of either cell type with *T. gondii* by Illumina sequencing. Transcript levels in infected and non-infected SkMCs, neurons, astrocytes and fibroblasts were analysed by RT-quantitative real-time PCR. **A:** The regulation of mRNA abundance following *T. gondii* infection (x-fold) as determined by RNAseq was compared for each gene and cell type with the corresponding data retrieved from real-time PCR. **B:** Scatterplot of the results indicated in A. The correlation coefficient r was obtained after a linear regression analysis.

Supplementary Figure S3

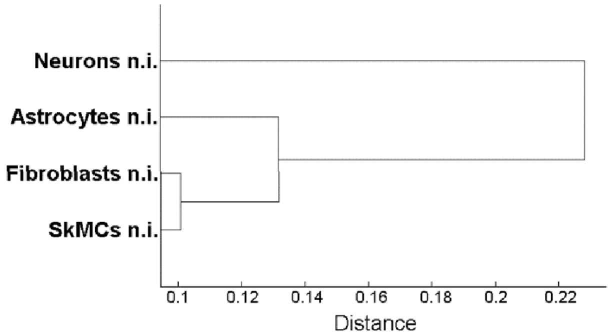

Hierarchical cluster analysis of genes that were differentially expressed in non-infected mature SkMCs, neurons, astrocytes or fibroblasts. Expression profiles were obtained by high-throughput RNA sequencing. Differentially expressed genes were identified by moderated Chi^2 test (FWER < 0.01).

Supplementary Table S1: Summary of Mapping Statistics

	SkMCs		Fibroblasts		Neurons		Astrocytes	
	ni[a]	*T. gondii*	ni	*T. gondii*	ni	*T. gondii*	ni	*T. gondii*
Illumina Sequencer	Genome Analyzer IIx	Genome Analyzer IIx	Genome Analyzer IIx	Genome Analyzer IIx	HiSeq2500	HiSeq2500	Genome Analyzer IIx	Genome Analyzer IIx
Read length	36	36	36	36	50	50	36	36
Total reads	32 928 565	29 070 147	37 403 318	35 352 668	94 193 783	107 842 244	50 126 239	47 375 351
Reference Genome I	*Mus musculus* [b]	*Mus musculus* [b]	*Mus musculus* [b]	*Mus musculus* [b]	*Mus musculus* [b]	*Mus musculus* [b]	*Mus musculus* [b]	*Mus musculus* [b]
Uniquely mapped	21 705 217	18 062 690	20 570 405	17 569 323	68 990 773	76 991 479	20 773 842	26 740 556
Non-specifically mapped	4 220 231	3 914 023	6 728 945	6 569 529	11 098 342	12 423 334	3 820 164	4 780 330
Total Mapped	25.925.448 (78.73%[c])	21.976.713 (75.6%)	27.299.350 (72.99%)	24.138.852 (68.28%)	80.089.115 (85.03%)	89.414.813 (82.91%)	24.594.006 (49.06%)	31.520.886 (66.53%)
Reference Genome II	*Toxoplasma gondii* [d]	*Toxoplasma gondii* [d]	*Toxoplasma gondii* [d]	*Toxoplasma gondii* [d]	*Toxoplasma gondii* [d]	*Toxoplasma gondii* [d]	*Toxoplasma gondii* [d]	*Toxoplasma gondii* [d]
Uniquely mapped	45	193 487	2 866	127 121	2 284	2 195 660	118	222 529
Non-specifically mapped	15	2 370	984	2 624	118	18 885	8	2 901
Total Mapped	*60 (0.00%)*	*195.857 (0.67%)*	*3.850 (0.01%)*	*129.745 (0.37%)*	*2.402 (0.00%)*	*2.214.545 (2.05%)*	*126 (0.00%)*	*225.430 (0.48%)*

[a]ni: non-infected

[b]mmMGSCv37

[c]Percentage of total reads

[d]TgondiiME49AnnotatedTranscripts_ToxoDB-7.0

Supplementary Table S2: Expression of host cell genes regulated by *T. gondii* in SkMCs (at least 2-fold; $p < 0.05$)
A: Genes up-regulated by *T. gondii*

	SKMCs				Neurons		Astrocytes		Fibroblasts	
**Gene ID**	**n.i.[a]**	***T. gondii***	**fold increase**	***p* value**	**n.i.**	***T. gondii***	**n.i.**	***T. gondii***	**n.i.**	***T. gondii***
AC165425.1	0.05	0.96	20.05	0.000264853	0.31	0.18	0.25	0.12	1.00	1.33
Fosb	0.27	4.83	18.06	1.131229619e-14	0.32	0.82	0.27	0.41	0.67	0.70
Adora2a	0.53	8.76	16.45	5.842846203e-20	2.61	2.83	0.11	0.23	0.00	0.02
Gm10063	1.10	17.06	15.44	6.199705438e-25	0.03	0.09	4.66	2.40	24.42	17.91
Pmch	0.05	0.79	15.34	0.000812406	0.18	0.31	0.38	0.55	1.13	0.89
Gpnmb	0.05	0.59	11.80	0.002371161	3.49	4.04	529.40	445.24	0.24	0.54
Rpl39l	0.09	1.04	11.21	0.00029117	0.02	0.74	0.69	1.03	132.59	154.24
Gm8225	0.04	0.40	9.44	0.026671534	0.01	0.02	0.09	0.07	0.28	0.37
RP23-232B3.2	0.04	0.37	9.44	0.038863876	0.04	0.01	0.21	0.06	0.26	0.29
RP23-321D1.4	0.73	6.50	8.85	5.583662307e-16	0.95	0.00	6.58	2.42	1.40	1.58
Gm3940	0.09	0.74	8.26	0.002052386	0.12	0.03	0.47	0.44	1.44	1.82
AC132393.1	0.06	0.53	8.26	0.004385902	0.37	0.43	0.47	0.68	0.24	0.14
1700029I15Rik	0.06	0.49	8.26	0.007120943	0.70	0.30	0.38	0.49	0.34	0.06
RP23-282N17.6	0.06	0.48	8.26	0.009716581	0.00	0.37	0.06	0.00	0.44	0.50
Gm5519	0.05	0.39	8.26	0.033492715	0.00	0.03	0.45	0.08	0.45	0.10
Egr2	2.27	17.69	7.79	4.294453809e-22	0.98	1.04	1.05	1.36	2.50	2.93
AC157924.3	0.12	0.89	7.67	0.001162098	0.28	0.19	4.24	3.35	0.11	0.25
Ptgs2	4.05	30.62	7.56	3.938170577e-23	0.10	0.12	2.65	3.78	131.28	106.99
Hist1h2af	0.29	2.05	7.08	5.398184047e-07	0.00	0.06	2.34	2.78	9.46	10.38
RP23-16C4.1	0.18	1.24	7.08	0.000177514	1.45	1.14	0.74	0.94	1.00	0.85
RP23-162P18.3	0.11	0.75	7.08	0.002491547	0.21	0.12	0.00	0.00	0.10	0.00
RP23-172B16.9	0.09	0.60	7.08	0.004199613	0.06	0.12	0.99	0.49	0.81	0.92
RP23-138L21.8	0.36	2.39	6.61	1.836691725e-07	0.00	0.00	3.06	1.31	1.51	0.70
AL732403.2	0.21	1.36	6.61	8.323876969e-05	0.33	0.07	1.85	1.19	0.00	0.04
Cldn15	0.07	0.47	6.49	0.014620115	0.04	0.24	0.15	0.46	0.02	0.12
Fos	1.61	9.91	6.16	3.602140132e-17	1.07	1.94	12.08	11.37	1.39	1.76
Nr4a1	7.93	47.59	6.00	1.838624802e-21	6.79	8.09	1.48	1.84	86.67	91.75
Cxcl1	0.20	1.19	5.90	0.000393843	0.12	0.99	0.60	1.13	1.23	1.43
Gm5048	0.07	0.39	5.90	0.044457519	0.09	0.04	0.14	0.22	0.13	0.86
Ccl9	0.13	0.73	5.78	0.00379433	0.41	0.37	23.10	20.91	3.42	3.74
Egr1	28.57	163.18	5.71	4.64922871e-21	16.42	19.64	12.16	17.42	37.65	57.70
Orc1l	0.12	0.69	5.58	0.004680472	0.16	0.32	0.38	0.45	3.80	3.61
Gm4554	0.42	2.33	5.51	6.302269193e-07	2.74	2.38	0.74	1.16	0.67	0.91
RP23-214E3.11	0.22	1.20	5.48	0.000457417	0.04	0.03	0.60	0.08	0.21	0.22
RP24-114E18.3	0.52	2.79	5.31	1.10047276e-07	0.08	0.04	0.55	0.32	1.12	0.28
Gm8290	0.15	0.80	5.31	0.003524688	0.00	0.00	2.30	0.43	4.87	3.72
Mt2	20.74	106.84	5.15	3.593874897e-20	102.87	93.25	893.15	753.82	156.70	238.23
AC153368.1	0.23	1.15	5.11	0.000757353	0.19	0.20	0.40	0.25	1.14	0.89
Sgol2	0.28	1.43	5.08	0.000114843	0.50	0.90	1.90	2.03	5.31	3.77
Arc	0.45	2.31	5.08	1.119946828e-06	1.23	1.26	0.51	0.64	1.76	2.09
Rpl36-ps3	1.41	6.99	4.97	1.197810163e-13	1.01	1.08	2.01	2.31	14.88	13.70
RP23-38E20.5	5.60	27.28	4.87	1.173624851e-19	1.50	1.09	19.06	27.51	46.51	47.36
Apobec3	0.64	3.13	4.87	3.656281383e-08	0.25	0.79	2.22	2.91	4.31	4.08
RP23-442K11.3	0.10	0.48	4.72	0.020419496	0.00	0.06	2.46	2.50	0.00	0.11
Rpl7a-ps10	0.10	0.45	4.72	0.028941021	0.03	0.08	0.20	0.08	0.82	0.47
Hist1h3i	0.09	0.44	4.72	0.03350419	0.00	0.19	0.49	0.00	2.14	1.92
Cxcl9	0.09	0.44	4.72	0.035409492	0.00	0.00	0.03	0.18	0.00	0.03
Dnm3os	0.58	2.76	4.72	2.875069818e-07	0.00	0.00	0.00	0.00	2.78	5.02
Gm4963	0.41	1.93	4.72	6.326764485e-06	0.07	0.09	1.72	1.68	0.48	0.33
Gm5614	0.12	0.57	4.72	0.009410865	0.04	0.11	0.00	0.30	1.04	2.74
3110021A11Rik	0.09	0.44	4.72	0.033285026	0.44	0.31	0.15	0.27	0.18	0.40
Gm7461	0.09	0.43	4.72	0.040129302	0.82	0.47	0.10	0.07	0.26	0.39
Lst1	0.09	0.41	4.72	0.049427518	1.82	1.10	36.39	31.94	2.87	3.15
Ttk	0.40	1.90	4.72	6.757366114e-06	0.44	0.53	0.87	0.95	1.98	1.85
Cd74	0.25	1.13	4.59	0.001215866	0.29	0.93	1.70	1.89	0.21	0.76
Il6	0.21	0.95	4.52	0.002925824	0.01	0.00	0.18	0.37	0.66	0.94
Psmb8	0.18	0.82	4.48	0.004807768	0.10	0.48	49.53	55.29	0.03	0.12
Asphd2	0.10	0.43	4.42	0.042149302	15.55	15.10	13.35	14.06	0.00	0.00
Pelo	1.72	7.50	4.36	4.015619394e-13	3.80	4.78	0.91	0.83	4.66	9.23
Speer4a	0.18	0.76	4.25	0.006873579	0.00	0.00	0.08	0.12	0.03	0.00
Troap	0.68	2.86	4.19	4.621793945e-07	1.14	1.37	2.34	2.77	11.84	12.84
Irf5	0.11	0.46	4.13	0.035448312	0.08	0.18	23.58	23.85	1.45	1.64
Espl1	0.55	2.24	4.05	6.268234873e-06	0.86	1.06	2.27	2.37	9.14	9.77
Ier2	31.85	128.38	4.03	9.25042647e-18	7.03	9.89	10.50	12.19	20.02	33.34
RP23-382C18.5	0.62	2.45	3.93	3.847922932e-06	0.00	0.02	0.15	0.23	5.91	2.90
RP23-396N8.2	0.76	2.96	3.88	5.90493315e-07	0.21	0.06	0.46	0.45	0.83	1.29
Kif15	0.37	1.42	3.87	0.000384123	0.99	1.27	2.57	2.65	4.94	3.72
Mybl2	0.42	1.59	3.75	0.000114396	1.28	2.62	1.66	2.20	4.72	5.99
Mcm10	0.20	0.75	3.75	0.009738859	0.57	0.74	1.28	1.35	3.43	3.44
Hist1h2ag	0.48	1.78	3.74	3.948189116e-05	0.75	0.32	1.59	0.72	6.65	16.66
Gtse1	1.10	4.08	3.69	1.240328564e-08	1.91	2.83	13.98	14.48	14.77	16.15
Adamts8	0.35	1.28	3.65	0.001215642	1.86	2.06	1.64	1.99	0.00	0.00
Nr4a3	0.14	0.49	3.60	0.030458314	39.28	40.19	2.52	2.96	1.00	0.91
Gm10043	0.29	1.02	3.54	0.004747079	0.15	0.36	0.20	0.16	0.64	0.82
Cdkn3	0.73	2.59	3.54	5.44626901e-06	0.53	1.08	6.47	6.34	4.81	5.44
RP23-49E16.2	1.59	5.62	3.54	6.706612291e-10	0.00	0.00	1.68	0.36	0.27	0.16
CH25-423E5.1	0.20	0.71	3.54	0.01275141	0.00	0.00	0.00	0.17	0.00	0.00
Gm5356	0.19	0.66	3.54	0.014486033	0.00	0.00	0.33	0.21	12.27	13.21
BC003266	0.14	0.49	3.54	0.030259619	0.09	0.12	0.44	0.34	0.39	0.30
Id3	3.40	11.97	3.52	4.169521361e-14	1.07	0.84	74.62	76.31	41.42	47.12
Shmt1	1.04	3.57	3.44	1.017680725e-07	1.51	2.27	2.34	2.78	9.88	10.98
Serpine1	19.77	67.32	3.41	7.405100246e-16	0.08	0.39	16.28	17.61	61.53	56.37
Casc5	0.22	0.74	3.39	0.013472085	0.39	0.56	1.40	1.31	2.34	1.59
Hist2h2ac	0.52	1.75	3.37	8.506675957e-05	0.07	0.54	0.70	0.43	4.02	9.16
RP23-282N17.2	0.15	0.49	3.34	0.033446675	0.00	0.21	0.00	0.06	0.33	0.18
Nuf2	1.58	5.27	3.34	3.852941888e-09	2.44	3.30	12.68	13.24	19.90	17.01
Enpp2	0.44	1.42	3.27	0.000888745	7.81	7.39	2.69	2.66	0.64	1.01

Nusap1	1.25	4.08	3.25	6.29188223e-08	2.54	3.37	6.32	5.97	8.19	6.66
Shc3	0.20	0.65	3.24	0.018870464	47.08	46.48	9.06	9.07	0.00	0.00
Errfi1	18.37	59.58	3.24	2.892903886e-15	5.57	6.72	5.82	6.09	61.06	52.28
Cenpa	1.76	5.65	3.22	2.812743262e-09	1.80	2.48	7.76	7.62	31.90	35.92
Ppapdc2	0.15	0.47	3.22	0.044788264	0.04	0.03	0.07	0.02	0.15	0.55
Chek1	0.50	1.61	3.19	0.000262533	0.75	1.24	1.53	1.93	4.61	4.22
Ung	0.66	2.11	3.19	5.320966355e-05	2.01	3.63	2.44	2.76	3.18	3.71
Kif20a	3.82	12.17	3.19	3.990213477e-13	2.96	4.04	14.03	13.83	40.21	35.19
Ang2	0.26	0.83	3.15	0.013016982	0.00	0.00	0.19	0.00	0.00	0.00
Depdc1b	0.23	0.72	3.15	0.017512798	0.27	0.38	1.37	1.02	2.92	2.90
AC124601.1	0.35	1.10	3.15	0.005254936	0.76	0.61	0.00	0.00	3.00	1.51
Chac2	0.18	0.55	3.15	0.025596241	1.03	0.72	0.40	0.46	0.78	0.79
Aurkb	1.62	5.10	3.14	1.451687701e-08	1.25	1.40	9.03	8.99	21.83	24.20
BC030867	0.29	0.92	3.13	0.010327563	0.97	1.48	1.34	1.55	4.84	4.48
Ccdc99	0.68	2.12	3.11	6.342189582e-05	0.63	0.81	2.64	2.50	6.49	5.97
AC133589.1	0.42	1.31	3.11	0.002158471	0.30	0.44	0.57	2.51	0.71	0.58
AC126554.3	0.17	0.52	3.10	0.028817096	0.03	0.01	0.00	0.02	0.00	0.00
Fabp5	0.80	2.47	3.09	2.549001151e-05	23.03	27.21	47.17	37.24	14.54	13.33
Pbk	2.39	7.37	3.08	2.373860395e-10	2.34	2.93	20.55	20.37	34.03	32.61
AC134539.2	0.64	1.96	3.07	0.00010459	3.76	6.37	0.13	0.21	2.55	3.02
Birc5	1.83	5.59	3.06	7.371313056e-09	1.91	2.96	6.48	5.94	18.86	19.57
CT030732.2	2.01	6.13	3.05	2.178171231e-09	1.54	1.75	11.43	11.55	21.40	26.02
Rab26	0.30	0.91	3.05	0.011611436	2.41	2.52	1.86	1.65	0.52	0.53
Olr1	0.23	0.69	3.03	0.021060056	0.00	0.01	0.09	0.13	0.01	0.03
Gm10252	0.42	1.26	3.01	0.00326257	11.71	10.24	4.54	3.32	2.42	1.54
Dlgap5	1.21	3.60	2.98	5.230321343e-07	2.22	3.11	5.44	5.67	10.30	9.61
Bub1	0.98	2.93	2.98	8.741665138e-06	0.74	0.86	4.33	4.31	9.67	7.92
Bub1b	1.81	5.40	2.98	1.819229605e-08	1.90	2.74	10.74	10.11	20.67	19.31
Gm10119	3.30	9.79	2.97	3.043641106e-11	0.13	0.91	1.33	0.44	0.32	0.26
Cyr61	88.36	261.01	2.95	4.317727298e-14	3.13	3.42	265.62	249.58	103.95	118.40
Ccl2	2.17	6.40	2.95	2.082065121e-09	0.08	3.93	12.86	17.84	30.19	38.37
AC122248.2 (7 128090547..12	0.20	0.58	2.95	0.029033756	3.59	2.85	0.72	1.78	1.40	0.53
RP23-22P24.2	0.18	0.52	2.95	0.032802495	0.03	0.03	0.09	0.29	0.42	0.56
Plk1	3.30	9.64	2.92	5.143253161e-11	5.12	6.77	15.22	16.27	31.68	34.69
C79407	0.29	0.85	2.90	0.016032841	0.37	0.49	1.21	1.31	1.99	1.61
Iqgap3	1.35	3.90	2.90	4.233036803e-07	0.56	0.88	7.39	7.95	11.04	10.59
Itpripl1	0.41	1.19	2.89	0.005282031	1.50	2.21	3.73	4.33	3.81	3.52
Tk1	0.22	0.62	2.89	0.028254151	0.10	0.17	1.64	1.57	9.04	11.82
Ccne2	0.72	2.07	2.89	0.000131611	0.99	1.99	1.42	2.10	2.02	1.93
Gm5830	18.68	53.80	2.88	9.190902785e-14	4.52	1.27	10.61	23.84	12.90	12.77
Fam83d	0.60	1.72	2.87	0.000285526	0.75	0.88	2.52	2.42	5.50	5.40
AC154517.3	0.48	1.39	2.86	0.002228856	0.00	0.00	0.66	0.68	1.71	2.52
Rad51	0.37	1.05	2.86	0.009399538	0.78	1.08	3.10	3.01	5.35	4.90
Fam54a	0.39	1.12	2.85	0.007344963	0.46	0.49	1.52	1.54	3.71	3.70
Trip13	0.83	2.37	2.85	7.050879052e-05	2.65	3.80	2.85	3.55	10.21	10.05
AC122242.2	0.75	2.12	2.85	0.000131288	0.00	0.00	0.00	0.00	0.33	0.33
Fignl1	1.08	3.06	2.84	8.908669162e-06	0.69	1.14	3.55	4.14	5.96	5.14
Mt1	43.32	122.96	2.84	1.431849309e-13	155.15	153.99	846.15	736.11	226.67	313.15
Lum	0.54	1.54	2.83	0.000954103	0.15	0.19	2.20	1.95	33.16	30.40
Rps12-ps2	0.48	1.37	2.83	0.002609052	0.03	0.11	0.41	0.64	3.21	2.18
Rnase1	0.40	1.13	2.83	0.00749161	0.05	0.03	0.92	0.95	0.00	0.00
2610002D18Rik	0.30	0.86	2.83	0.017348784	0.54	0.75	2.87	1.49	4.05	4.48
Ube2c	4.87	13.76	2.83	4.317001958e-12	5.61	6.44	8.87	8.88	31.15	33.83
Mcm5	4.12	11.63	2.82	1.074049382e-11	5.97	10.26	21.04	21.47	62.69	65.91
Id1	5.72	16.12	2.82	1.702782465e-12	1.31	1.15	36.31	37.14	16.17	18.57
Cdca7	1.00	2.81	2.81	2.270235235e-05	4.06	5.14	3.40	4.62	13.98	13.51
Btg2	8.02	22.51	2.81	1.971328371e-13	2.36	2.77	17.06	17.87	10.64	9.95
Gm5879	0.25	0.71	2.80	0.025448722	0.06	0.05	1.11	1.02	0.70	0.68
Gm12666	0.40	1.13	2.80	0.00767887	0.07	0.26	0.05	0.08	0.00	0.05
5530401N12Rik	0.39	1.10	2.80	0.008517818	0.21	0.17	0.57	0.89	0.61	0.95
Ucma	0.35	0.98	2.80	0.012539578	0.31	0.17	1.11	1.01	0.00	0.05
E2f7	0.99	2.75	2.79	3.022254613e-05	0.70	1.22	1.85	1.82	5.24	5.02
Rpl13-ps3	0.68	1.90	2.79	0.000253515	0.10	0.09	1.24	1.17	2.00	7.57
Aph1c	0.35	0.97	2.79	0.013394991	0.92	0.71	7.73	7.28	1.08	1.09
Cenpm	1.19	3.30	2.77	4.934051631e-06	5.65	5.91	5.25	5.63	27.27	35.46
Ncapg	0.65	1.80	2.77	0.000329549	0.36	0.46	2.16	2.22	6.18	5.40
Chaf1b	0.74	2.05	2.76	0.000197432	1.13	3.13	2.27	2.21	4.94	5.40
Wdhd1	0.62	1.71	2.76	0.000397894	0.66	1.48	1.95	2.35	3.53	3.73
Ttpal	0.38	1.06	2.76	0.01062941	1.11	1.24	0.21	0.33	0.51	3.33
Ccnb1	3.15	8.68	2.75	6.062995154e-10	2.06	3.02	12.67	13.49	31.91	29.78
2610021K21Rik	0.22	0.62	2.75	0.033011724	0.20	0.32	2.81	2.61	1.10	1.20
Snhg9	0.63	1.74	2.75	0.000381255	7.30	8.62	2.44	1.91	1.40	1.36
Gm10028 (5 113489511..1134	0.43	1.19	2.75	0.006658118	0.09	0.23	0.00	0.42	0.55	0.62
RP24-406J12.2	0.40	1.09	2.75	0.009456756	0.13	0.12	0.84	0.22	2.39	4.41
RP23-451D4.2	0.28	0.76	2.75	0.024041684	0.21	0.05	0.29	0.38	0.35	0.30
Gm5481	0.27	0.75	2.75	0.025048834	0.12	0.05	0.00	0.45	1.37	3.69
AC113270.1	1.35	3.72	2.75	1.12838094e-06	0.02	0.00	0.54	0.95	1.28	4.90
Cdc20	5.58	15.35	2.75	4.609955924e-12	3.79	5.27	19.61	21.99	92.54	101.35
RP24-118H2.2	0.88	2.43	2.75	8.415380513e-05	0.00	0.02	0.00	0.00	0.00	0.00
Rpl21-ps8	0.21	0.59	2.75	0.034873408	0.14	0.17	0.08	0.53	1.82	1.30
Kif11	1.49	4.08	2.74	7.044730892e-07	2.37	2.97	7.61	7.93	15.73	13.40
Ccnb2	4.56	12.48	2.74	1.697863138e-11	2.04	2.80	19.55	18.71	11.72	10.96
Zwilch	0.75	2.03	2.72	0.000237403	1.08	1.38	3.28	3.13	5.16	4.28
Esco2	0.34	0.91	2.70	0.018084007	0.29	0.44	1.99	2.11	3.23	2.09
RP23-288N2.2	0.30	0.80	2.70	0.023798321	0.00	0.00	0.00	0.00	0.08	0.00
2310046A06Rik	0.23	0.61	2.70	0.035495128	5.45	6.52	0.34	0.13	0.00	0.00
Myc	8.57	23.09	2.69	7.12638636e-13	24.39	29.41	6.23	8.63	34.85	35.02
Exo1	0.50	1.35	2.68	0.003873842	0.91	1.27	1.74	2.02	7.97	7.61
Ckap2	2.21	5.90	2.66	4.507884096e-08	3.99	5.41	19.48	20.15	22.86	19.88
Fanci	0.29	0.77	2.66	0.026852449	0.56	0.67	1.83	1.88	3.60	3.08
2310001K24Rik	0.35	0.93	2.65	0.017952562	0.06	0.25	0.00	0.00	0.00	0.28

Tmem171	0.19	0.52	2.65	0.044108833	0.00	0.49	2.93	2.22	1.23	1.07
Ndc80	1.65	4.37	2.65	7.278301865e-07	1.97	3.09	5.39	5.12	17.96	16.18
Fancd2	0.26	0.68	2.65	0.032584967	1.25	1.12	1.16	1.42	2.39	1.95
Ckap2l	1.66	4.40	2.65	7.259659629e-07	7.77	10.42	9.92	10.74	10.43	8.81
Cdca2	0.40	1.06	2.64	0.0127665	1.00	1.14	1.79	1.90	3.20	2.92
Prr11	1.31	3.46	2.64	4.766266732e-06	1.65	2.30	3.75	4.05	9.32	7.18
Melk	1.12	2.94	2.63	3.136814242e-05	1.43	2.19	5.31	5.57	7.68	6.86
Top2a	3.94	10.34	2.63	2.874113603e-10	6.05	8.29	17.69	19.51	69.36	55.75
AC147612.2	0.44	1.15	2.62	0.009738962	0.05	0.01	0.98	0.64	1.39	4.13
Ercc6l	0.49	1.30	2.62	0.005549712	0.33	0.57	2.12	2.34	5.62	4.60
Foxm1	1.87	4.88	2.62	4.001120148e-07	4.56	5.83	7.71	7.93	16.76	15.46
Cdc2a	4.93	12.84	2.61	5.595649328e-11	1.40	2.24	14.53	15.86	43.21	43.42
Shcbp1	1.81	4.71	2.60	5.756383562e-07	1.90	2.40	5.30	5.74	13.90	11.45
Aspm	0.49	1.27	2.60	0.006458779	1.55	1.93	3.03	3.08	3.30	2.57
Ctxn3	3.18	8.25	2.59	3.625165005e-09	0.00	0.00	0.38	0.29	0.00	0.03
Dctd	2.23	5.74	2.58	1.08669115e-07	7.12	7.92	3.81	4.54	11.36	12.12
Kif14	0.23	0.59	2.57	0.042324398	0.29	0.36	0.71	0.80	1.47	1.19
Cep55	1.22	3.13	2.56	2.299317394e-05	1.35	2.15	5.65	5.51	9.80	9.09
Rps2-ps6	0.81	2.07	2.56	0.000369528	0.03	0.01	0.05	0.04	0.73	1.55
Fam64a	2.79	7.11	2.55	1.553718808e-08	2.42	2.97	8.68	9.52	25.20	27.19
Gnai1	0.23	0.58	2.55	0.044490704	40.16	41.08	3.73	3.48	0.00	0.01
AC130841.2	9.34	23.74	2.54	4.397003312e-12	28.21	42.72	53.66	54.92	138.08	144.28
Depdc1a	0.79	2.00	2.53	0.000457731	0.36	0.49	1.39	1.57	4.21	3.09
Hmmr	1.32	3.34	2.53	1.323075619e-05	0.93	1.10	4.19	4.57	13.43	10.47
Gm4950	0.44	1.10	2.53	0.013340606	0.02	0.02	1.32	0.67	0.89	0.47
Kif23	1.51	3.80	2.52	3.650617753e-06	1.69	2.10	7.13	7.14	12.51	10.71
Ctgf	46.19	116.04	2.51	6.369516613e-12	1.51	1.49	201.77	196.80	55.96	57.58
Clspn	0.59	1.47	2.51	0.003130812	1.07	1.71	2.59	2.68	6.36	5.84
RP23-16H24.2	0.34	0.84	2.51	0.027848088	0.18	0.27	1.55	1.42	0.60	0.72
E130306D19Rik	0.23	0.58	2.51	0.047147076	0.41	0.28	2.49	2.17	2.33	2.34
Rps2	4.51	11.29	2.50	3.242190062e-10	53.45	60.17	64.37	71.29	14.41	14.60
Ier3	17.42	43.59	2.50	7.252313248e-12	1.46	1.83	15.09	16.86	32.73	35.83
Dusp5	3.03	7.56	2.50	1.561351018e-08	1.35	1.86	4.92	4.80	20.57	20.42
Sigirr	0.39	0.96	2.48	0.021960844	0.00	0.03	0.22	0.14	2.80	3.21
Chaf1a	2.80	6.92	2.47	3.413566964e-08	5.25	6.62	8.09	8.16	25.54	25.81
Cenpe	1.03	2.54	2.47	0.000184573	1.61	2.19	3.85	4.14	9.06	6.99
Kif22	2.69	6.60	2.46	5.017074098e-08	3.63	4.42	9.65	9.20	20.30	22.68
Rps12	88.00	215.91	2.45	1.353567991e-11	95.21	92.44	44.20	73.08	975.09	1264.70
Tpx2	3.89	9.52	2.44	4.165129299e-09	9.11	12.86	14.33	15.84	29.06	27.30
Ncaph	1.51	3.68	2.44	6.713403995e-06	1.07	1.50	6.17	6.59	9.83	9.16
Gm6404	8.66	21.09	2.44	1.698842798e-11	0.61	0.46	6.25	6.34	153.49	200.67
Cdca8	1.30	3.17	2.43	3.967790884e-05	3.00	3.92	5.41	5.16	14.79	15.15
Nr4a2	1.36	3.30	2.42	2.666967997e-05	2.13	2.69	0.48	0.66	9.68	10.46
AC102377.2	30.22	73.14	2.42	2.094885734e-11	59.75	56.07	28.48	23.51	65.58	81.24
Mcm2	7.13	17.25	2.42	9.209969985e-11	3.93	6.68	17.98	21.12	51.63	54.72
Nfkbie	4.46	10.78	2.42	1.462339173e-09	1.03	1.39	5.30	6.41	2.50	3.92
Serinc4	0.34	0.83	2.42	0.032909069	1.66	3.04	4.13	2.27	2.04	0.58
Stmn1	13.53	32.67	2.42	2.243730154e-11	775.22	752.72	77.45	75.17	35.72	35.69
Nek2	1.44	3.49	2.42	1.467714694e-05	0.99	1.35	5.12	5.66	10.89	10.76
RP23-264D16.1	0.29	0.70	2.40	0.043100352	0.00	0.00	0.00	0.01	0.00	0.00
Ccna2	4.09	9.80	2.40	5.079579667e-09	8.63	11.65	15.02	16.11	40.43	37.77
Gm6166	13.47	32.25	2.39	2.970553931e-11	0.22	1.21	0.54	0.47	237.86	264.75
2010317E24Rik	0.39	0.92	2.39	0.02769628	0.84	0.98	1.06	0.97	3.57	4.95
Rpl36	41.90	100.03	2.39	3.256422325e-11	233.52	250.54	33.65	35.70	501.74	949.68
Dna2	0.30	0.72	2.39	0.043074398	0.20	0.33	1.21	1.44	1.54	1.56
E330016A19Rik	0.45	1.07	2.38	0.01929645	0.45	0.45	1.16	1.68	2.95	2.82
Aurka	2.32	5.52	2.38	7.093019708e-07	2.32	3.46	6.27	7.12	21.00	22.05
Uhrf1	3.00	7.14	2.38	6.464732859e-08	3.75	6.11	10.67	11.43	38.11	41.98
Rgs16	2.57	6.11	2.37	2.68450854e-07	3.40	3.28	15.14	15.86	14.41	17.48
Fbxo5	1.81	4.29	2.37	4.73049708e-06	1.65	2.61	4.45	5.69	16.00	17.15
Ppan	6.42	15.19	2.37	3.800254225e-10	6.37	6.92	6.11	7.40	17.60	23.49
Fancb	0.35	0.82	2.36	0.036502441	0.53	0.60	1.05	1.17	2.97	2.49
Gm1673	0.29	0.68	2.36	0.047735241	49.17	52.69	17.73	19.75	9.53	11.52
Rpl41 (9 24905845..24905927)	0.99	2.33	2.36	0.000452603	0.16	0.29	1.56	2.44	6.57	7.97
AC160134.2	0.92	2.18	2.36	0.000609958	0.00	0.02	0.00	0.00	2.00	2.26
Rnf125	0.49	1.15	2.36	0.015791917	0.29	0.28	1.14	0.95	0.61	0.39
Eme1	0.35	0.82	2.36	0.036501609	1.13	1.47	1.55	1.61	3.63	4.05
Hist1h4h	0.33	0.77	2.36	0.04031393	0.93	0.76	14.79	22.68	5.61	15.42
Gsta1	0.32	0.76	2.36	0.041346298	0.05	0.02	0.00	0.00	0.04	0.04
AC122296.1	0.28	0.67	2.36	0.049027447	0.21	0.05	0.40	0.16	0.00	0.00
Csrnp1	3.20	7.54	2.35	5.967207237e-08	0.40	0.38	3.26	3.56	2.95	3.00
F630043A04Rik	0.90	2.11	2.34	0.000753469	1.82	1.92	2.55	3.05	5.84	5.30
Ect2	1.48	3.45	2.33	2.702323249e-05	1.22	1.64	7.18	7.58	12.41	9.81
Rad51ap1	0.30	0.70	2.32	0.049252924	0.61	0.84	1.64	1.83	3.50	3.45
Prickle4	0.33	0.75	2.32	0.044567018	12.62	9.73	3.16	2.31	1.32	1.16
Mcm7	6.97	16.11	2.31	5.541155424e-10	6.09	7.89	14.36	16.95	42.35	48.31
Cdc25c	0.96	2.21	2.31	0.000702767	0.48	1.09	2.29	2.51	10.37	9.46
Mki67	2.65	6.12	2.31	4.571481758e-07	11.52	13.50	14.51	15.90	17.44	12.37
Kif4	0.89	2.05	2.30	0.000968019	1.99	2.27	4.36	4.33	9.81	8.02
Pop7	1.21	2.78	2.29	0.000233003	1.10	0.81	1.50	0.70	1.05	0.96
Dtl	1.10	2.51	2.28	0.00043432	1.23	1.97	3.60	3.89	11.41	9.89
Cdca7l	2.07	4.72	2.28	5.06166084e-06	0.97	1.66	4.56	5.10	14.63	15.20
Brca1	0.45	1.02	2.28	0.026787493	0.60	0.81	2.00	2.29	3.74	3.13
Recql4	0.51	1.17	2.27	0.01814606	0.53	0.83	1.08	0.93	5.76	6.21
D2Ertd750e	1.92	4.35	2.27	8.815719178e-06	1.40	1.51	5.65	5.91	12.55	12.62
Gm10193	0.74	1.67	2.27	0.002032736	0.07	0.11	1.31	0.98	2.69	0.91
Mnd1	1.72	3.90	2.27	1.53986622e-05	0.39	0.46	0.56	0.58	6.08	7.25
Apln	1.54	3.50	2.27	3.478097892e-05	0.40	0.61	5.18	5.15	7.28	7.85
Nup62	4.05	9.16	2.26	3.812741456e-08	3.74	4.97	4.38	3.82	14.44	17.23
Lrdd	0.43	0.97	2.26	0.031605994	1.32	1.54	3.59	4.15	1.84	2.25
Dctpp1	12.61	28.41	2.25	2.185354453e-10	15.26	21.41	11.01	13.00	23.84	38.61

Cldn2	0.79	1.78	2.25	0.001814204	0.03	0.04	3.23	2.47	0.00	0.00
Cdc6	0.53	1.19	2.24	0.01819626	0.33	0.56	1.58	1.56	5.86	5.69
Arhgap11a	1.80	4.03	2.24	1.657872976e-05	2.75	3.53	7.27	8.22	10.94	9.11
Kifc1	1.13	2.53	2.24	0.000520441	0.64	1.03	1.93	2.62	11.56	12.28
Gins1	1.40	3.12	2.23	0.00013321	1.12	1.54	3.59	4.08	6.52	8.17
Diap3	2.14	4.77	2.23	7.072603144e-06	0.88	0.96	5.22	5.80	11.69	9.59
Nsl1	0.61	1.37	2.23	0.010228975	0.48	0.63	2.50	2.35	5.01	5.35
Ska1	0.64	1.42	2.23	0.008278379	1.72	2.17	2.21	2.16	11.63	10.10
4632434I11Rik	0.41	0.92	2.23	0.037049789	0.76	0.74	3.36	3.60	2.40	1.78
Cdt1	2.89	6.42	2.22	5.869778998e-07	1.85	4.41	9.23	10.00	37.74	42.11
Edn1	1.28	2.83	2.21	0.00030862	0.04	0.04	2.30	2.03	0.21	0.21
Incenp	4.12	9.05	2.20	8.789325571e-08	11.95	14.09	18.22	18.08	29.47	29.29
BC048355	0.72	1.57	2.19	0.004620374	0.06	0.14	0.79	0.87	2.21	2.50
RP23-317F9.4	0.39	0.85	2.19	0.046038067	0.70	0.89	0.94	0.27	0.21	0.24
Elovl4	0.38	0.83	2.19	0.048209738	24.84	26.41	1.52	1.27	0.27	0.25
Traip	0.82	1.79	2.19	0.002308583	5.99	5.81	2.14	2.28	6.73	7.27
Cenpi	0.71	1.55	2.18	0.005327141	0.36	0.64	1.57	1.51	3.31	2.98
AA467197	0.80	1.74	2.17	0.002621442	0.03	0.06	2.70	2.82	0.18	0.28
Pttg1	2.51	5.44	2.17	4.633527962e-06	1.99	2.42	10.27	10.95	10.02	8.49
Hist1h2ac	0.97	2.10	2.16	0.00157208	0.16	0.05	4.27	3.47	3.39	11.58
Gzme	0.49	1.07	2.16	0.030433472	0.00	0.00	0.22	0.37	0.00	0.00
Gm16415	0.43	0.93	2.16	0.041716401	0.07	0.06	0.83	1.00	1.49	0.84
Dbil5	0.40	0.86	2.16	0.047514922	0.34	0.38	0.00	0.44	0.44	0.14
Gm10762	0.67	1.44	2.16	0.009426846	4.41	4.82	4.21	2.46	1.26	1.43
Anln	7.06	15.25	2.16	5.415998857e-09	0.97	1.54	12.07	12.58	46.82	37.75
Hey1	1.94	4.19	2.16	2.452615737e-05	6.09	6.24	24.60	24.90	0.02	0.02
Ccnf	2.07	4.46	2.16	1.827167427e-05	2.73	3.56	7.23	7.54	16.58	17.14
Kif2c	1.46	3.14	2.15	0.000200452	1.24	1.65	5.00	4.95	19.87	18.71
Cdca3	1.56	3.35	2.15	0.000117536	3.17	4.16	10.29	9.56	14.25	14.54
Gm8394	0.44	0.94	2.14	0.041789545	0.01	0.01	0.17	0.69	1.94	0.47
Cdc7	0.56	1.20	2.14	0.022323194	3.13	3.23	1.04	1.31	4.93	3.85
AC154586.1	7.08	15.11	2.14	8.132250776e-09	0.81	1.45	63.09	62.99	75.90	98.75
Tacc3	3.88	8.28	2.13	3.24998728e-07	2.90	3.81	12.17	11.78	26.60	25.79
Lmnb1	4.25	9.05	2.13	1.878822492e-07	19.64	22.63	19.27	21.12	49.72	48.80
Cenph	0.67	1.43	2.13	0.010794358	0.69	0.88	2.28	2.22	9.96	9.68
Gm10355	0.74	1.58	2.12	0.005736466	0.36	0.34	0.55	0.67	1.76	3.51
2410022M11Rik	0.58	1.22	2.12	0.022067881	0.55	0.58	1.54	1.93	0.98	1.28
Mcm3	5.66	12.02	2.12	2.296265302e-08	4.84	7.44	20.44	23.00	56.43	57.01
Ch25h	2.11	4.46	2.12	2.431541319e-05	0.00	0.02	16.77	17.75	0.00	0.00
Psrc1	1.29	2.73	2.11	0.000630228	2.23	2.13	17.06	18.02	11.72	13.03
Asf1b	3.56	7.53	2.11	6.959804097e-07	0.98	1.43	7.17	6.65	14.82	13.86
Gpr19	0.58	1.22	2.11	0.022991418	2.77	3.32	8.88	9.86	0.84	0.64
Hells	0.77	1.62	2.11	0.00487835	0.57	0.83	1.93	2.36	9.81	8.35
Kntc1	0.72	1.52	2.11	0.008132238	0.56	0.70	2.51	2.78	9.09	7.25
Kif18b	1.14	2.41	2.10	0.001237448	2.30	3.00	4.52	4.68	8.62	8.25
Gm5921	3.76	7.89	2.10	6.389958472e-07	0.08	0.11	0.41	0.11	0.29	1.46
Fam60a	3.65	7.63	2.09	8.085966353e-07	0.16	0.20	1.34	1.75	8.06	8.64
Blm	0.53	1.11	2.09	0.031723534	1.99	2.03	1.49	1.82	5.26	4.42
Mcm6	12.64	26.40	2.09	2.673679574e-09	8.35	11.53	32.06	40.43	50.23	50.00
Sgol1	0.62	1.29	2.09	0.019759243	0.61	1.05	2.16	2.16	7.20	5.86
Cks1b	10.24	21.38	2.09	2.748850576e-09	2.47	3.80	21.59	22.23	86.60	95.84
AC150277.2	1.64	3.40	2.08	0.000153611	6.17	7.17	5.17	3.72	22.56	30.68
Tmem194b	0.48	0.99	2.08	0.043721944	0.72	0.89	1.79	1.71	2.15	2.19
Lrp8	0.66	1.36	2.08	0.016151671	20.60	20.73	7.32	8.24	12.18	11.96
Hspa2	18.47	38.33	2.08	3.305366527e-09	7.01	6.09	23.55	23.18	5.43	6.04
Gm5577	0.66	1.37	2.06	0.016595389	4.16	3.72	3.16	3.05	0.75	0.45
Chek2	1.15	2.37	2.06	0.001634017	0.60	0.86	6.08	6.68	5.62	3.96
H2afx	5.57	11.47	2.06	6.434697033e-08	38.44	42.69	31.67	36.84	38.17	50.80
Fbl	16.88	34.57	2.05	5.159490084e-09	30.26	41.84	31.55	40.00	105.66	142.26
Nop56	12.13	24.83	2.05	5.236310958e-09	20.57	24.73	12.20	16.08	59.73	62.37
Junb	85.68	175.33	2.05	5.316354745e-09	4.49	5.49	22.67	24.52	24.67	37.66
Gen1	0.56	1.14	2.05	0.033717789	0.50	0.64	1.19	1.43	2.64	2.53
Cks2	14.38	29.26	2.03	6.43424171e-09	2.38	3.56	23.56	24.89	68.50	79.20
Gas2l3	0.70	1.43	2.03	0.014586323	0.68	0.90	2.72	2.71	3.59	3.12
Cenpf	1.20	2.44	2.03	0.00174104	1.69	2.15	4.60	4.94	7.77	5.46
AC076974.1	2.51	5.08	2.02	2.664035307e-05	0.00	0.02	0.38	0.18	9.82	12.81
Atad2	2.61	5.28	2.02	2.157543402e-05	2.37	3.16	5.41	6.21	18.58	14.56
C330027C09Rik	1.21	2.45	2.02	0.00178565	1.00	1.35	3.64	3.70	8.35	6.52
Mastl	0.99	1.98	2.00	0.003890868	0.46	0.60	1.87	1.92	3.84	2.70
AC122193.1	0.00	0.95	n.d.[b]	0.000132585	0.00	0.00	0.00	0.00	1.53	0.00
Gm7696	0.00	0.60	n.d.	0.000856791	0.36	0.22	0.45	0.42	0.41	0.55
4930524J08Rik	0.00	0.60	n.d.	0.000858064	0.52	0.41	1.42	1.32	0.85	0.96
Hist1h2bh	0.00	0.55	n.d.	0.001078178	0.00	0.00	0.20	0.92	3.81	6.22
AC105966.2	0.00	0.53	n.d.	0.00118038	0.00	0.13	0.00	0.00	0.00	0.00
AC188461.2	0.00	0.52	n.d.	0.001239827	0.00	0.00	0.46	0.18	0.00	0.00
Hist1h4a	0.00	0.51	n.d.	0.001576082	0.23	0.08	1.36	0.77	4.49	15.06
AL606521.1	0.00	0.45	n.d.	0.006567081	0.00	0.00	0.00	0.00	0.00	0.00
AL928792.1	0.00	0.40	n.d.	0.014229957	0.00	0.00	0.00	0.00	0.00	0.00
2510046G10Rik	0.00	0.39	n.d.	0.015663513	0.14	0.58	0.23	0.55	0.11	0.95
4933416I08Rik	0.00	0.39	n.d.	0.017229912	1.93	1.85	0.10	0.61	0.00	0.25
Gm5528	0.00	0.38	n.d.	0.019168827	0.44	0.05	0.00	0.85	0.83	0.26
1700001G17Rik	0.00	0.37	n.d.	0.021838588	0.37	0.37	0.28	0.29	0.88	0.57
Gm10725	0.00	0.35	n.d.	0.025107177	0.51	0.51	3.61	4.23	0.37	0.09
AC116115.2	0.00	0.34	n.d.	0.027726783	0.00	0.00	0.00	0.00	0.00	0.00
Gm4925	0.00	0.34	n.d.	0.028489351	0.00	0.00	0.51	0.32	0.18	0.10
AL606479.1	0.00	0.34	n.d.	0.028870201	0.00	0.00	0.00	0.00	0.00	0.00
AC131340.2	0.00	0.33	n.d.	0.031146998	0.00	0.20	0.07	0.00	0.20	0.08
Gm10507	0.00	0.33	n.d.	0.031524793	0.00	0.00	0.15	0.00	0.00	0.45
Hist1h4d	0.00	0.32	n.d.	0.034525019	0.38	0.29	1.25	1.35	10.74	23.01
AL807745.1	0.00	0.32	n.d.	0.036744587	0.00	0.00	0.00	0.22	0.76	0.29
Plac8	0.00	0.31	n.d.	0.037278163	0.03	0.18	0.13	0.20	1.13	1.14

Gm2046	0.00	0.31	n.d.	0.037477781	0.00	0.00	0.09	0.00	0.08	0.00
6530403G13Rik	0.00	0.30	n.d.	0.041088227	0.04	0.00	0.00	0.21	0.00	0.14
RP23-360J20.2	0.00	0.30	n.d.	0.042034376	0.00	0.00	0.00	0.00	0.08	0.00
Gm9952	0.00	0.29	n.d.	0.044251635	1.01	2.90	0.00	0.10	0.00	0.13
Rpl23a-ps1	0.00	0.29	n.d.	0.045286854	0.45	0.29	0.52	0.07	0.39	0.53
AC140327.2	0.00	0.29	n.d.	0.046312149	0.20	0.21	0.00	0.10	0.00	0.00
Fabp4	0.00	0.29	n.d.	0.047495628	0.00	0.60	11.36	6.23	0.58	0.85
Gm10766	0.00	0.28	n.d.	0.049327405	0.66	0.45	0.00	0.10	0.23	0.26
Hist1h3h	0.00	0.28	n.d.	0.049766274	0.28	0.18	0.08	0.13	3.45	9.00
AC161876.1	0.00	1.23	n.d.	1.410262009e-05	0.00	0.10	0.37	0.00	0.66	0.37
Gm10197	0.00	1.58	n.d.	1.593648369e-07	0.00	0.00	0.61	0.95	0.18	0.41

B: Genes down-regulated by *T. gondii*

	SKMCs				Neurons		Astrocytes		Fibroblasts	
**Gene ID**	**n.i.**	***T. gondii***	**fold decrease**	***p* value**	**n.i.**	***T. gondii***	**n.i.**	***T. gondii***	**n.i.**	***T. gondii***
Camk2n2	0.60	0.04	16.95	0.001750335	7.51	11.01	0.63	0.20	0.48	0.48
Gm16374	0.55	0.04	14.41	0.002377423	0.33	0.32	0.31	0.11	0.28	0.14
Slc22a15 (3 101686939..10172	0.50	0.05	11.02	0.004974472	0.17	0.26	0.16	0.16	0.11	0.08
RP24-103E20.2	3.41	0.32	10.60	4.093807186e-11	1.32	0.99	0.43	1.23	1.29	1.76
Tpm3-rs7	0.43	0.04	9.75	0.019240811	0.12	0.15	1.04	0.80	0.58	0.54
RP23-298F22.4	1.78	0.23	7.63	1.084135176e-06	0.19	0.03	0.21	0.08	0.56	0.96
Hist1h2bj	1.37	0.19	7.21	5.788826195e-05	0.13	0.14	2.30	1.60	3.46	8.16
Gm4832	0.82	0.12	6.78	0.001978567	0.00	0.00	0.54	0.25	0.29	0.11
CT030013.1	0.65	0.11	5.93	0.004766634	0.30	0.22	0.39	0.15	0.27	0.30
AC162689.1	0.57	0.11	5.09	0.008236093	0.21	0.03	0.40	0.31	0.27	0.51
Gm10572	0.76	0.15	5.09	0.004528935	0.41	0.33	0.27	0.41	0.24	0.00
AL627237.1	0.66	0.13	5.09	0.006285183	0.09	0.03	0.35	0.59	1.09	0.41
RP23-17O4.3	0.50	0.10	5.09	0.012248097	0.22	0.30	1.50	1.92	0.48	0.54
RP23-103L13.9	0.42	0.08	5.09	0.03980017	0.00	0.00	0.96	1.49	0.46	0.08
Gm6139	3.76	0.77	4.86	1.141703614e-09	0.01	0.01	0.14	0.00	0.54	1.64
CT027556.2	2.62	0.59	4.43	8.144257418e-07	1.34	0.36	0.88	0.14	2.23	1.44
Ccr9	0.42	0.10	4.41	0.045492459	0.00	0.00	0.00	0.05	0.00	0.00
RP23-57N22.5	0.57	0.14	4.24	0.011790678	0.00	0.03	0.06	0.05	0.05	0.00
Pnma1	0.54	0.13	4.24	0.012687021	1.13	0.95	0.27	0.21	0.38	0.31
1500002C15Rik (4 155107970.	0.50	0.12	4.24	0.018144784	1.16	1.99	0.42	0.49	0.76	0.64
Hist1h2bm	0.47	0.11	4.24	0.02732996	0.00	0.03	0.30	0.23	0.99	2.03
AC157903.1	0.45	0.11	4.24	0.037677022	0.20	0.03	0.09	0.37	0.59	0.67
Gm5321	0.43	0.10	4.24	0.044579558	0.06	0.00	0.18	0.07	0.08	0.09
Gm12353	1.04	0.25	4.24	0.002328121	0.54	0.63	1.83	1.55	0.07	0.07
Gm10479	0.72	0.17	4.24	0.007877457	0.00	0.00	0.00	0.06	0.20	0.15
Gm5805	1.80	0.44	4.07	2.21010464e-05	0.29	0.35	0.87	1.79	3.27	1.69
AC164158.1	4.29	1.14	3.77	6.154958569e-09	0.89	1.52	2.35	2.32	1.30	2.62
AC153851.1	1.47	0.40	3.67	0.000337426	0.00	0.00	0.00	0.00	0.00	0.00
AC154200.2	0.73	0.20	3.67	0.010825591	0.07	0.08	0.18	0.56	0.43	0.30
Tslp	1.27	0.36	3.48	0.001612296	0.00	0.00	0.00	0.03	0.03	0.00
Gm9790	1.30	0.38	3.39	0.001534762	0.55	0.21	1.20	1.67	0.85	2.01
Hist2h4 (3 96066913..9606723:	0.49	0.14	3.39	0.03298561	1.19	0.67	0.26	0.30	3.60	8.29
AC127419.2	0.49	0.14	3.39	0.03298561	0.16	0.07	0.52	0.70	0.93	0.79
AC163616.2	0.48	0.14	3.39	0.037241189	0.31	0.05	0.51	0.30	0.17	0.13
Ysk4	1.58	0.47	3.39	0.000223536	0.00	0.23	4.59	4.88	0.19	0.00
G630090E17Rik	0.59	0.18	3.27	0.021561311	0.05	0.03	0.87	1.11	0.60	0.40
Ucp3	0.54	0.17	3.11	0.027118658	0.00	0.00	0.01	0.00	0.02	0.04
Wdr16	0.48	0.16	3.07	0.045442092	0.16	0.20	13.44	12.70	0.00	0.00
RP23-173F3.1	3.11	1.03	3.02	3.861210926e-06	0.00	0.02	0.11	0.13	165.67	226.03
AL928605.2	1.17	0.39	2.97	0.005169046	0.00	0.00	0.00	0.14	0.16	0.00
Igh-5	0.85	0.29	2.88	0.016660715	0.02	0.00	0.00	0.00	0.05	0.00
9430025M13Rik	0.65	0.23	2.88	0.026813296	2.05	2.26	0.28	0.91	0.33	0.25
Gt(ROSA)26Sor	1.40	0.50	2.81	0.002277074	0.37	0.26	0.14	0.35	3.07	2.08
RP24-103K4.2	0.61	0.22	2.79	0.032135996	0.40	0.33	1.06	0.59	0.53	0.40
Xcr1	0.71	0.26	2.78	0.026256103	0.00	0.00	0.04	0.10	0.01	0.02
Hyal1	0.62	0.23	2.72	0.033987039	0.16	0.17	2.61	2.98	0.83	0.39
AC167249.3	58.36	21.83	2.67	9.000173617e-13	33.13	16.31	70.50	62.02	89.71	31.29
Phox2a	1.84	0.70	2.62	0.000469487	0.03	0.00	0.17	0.44	0.05	0.15
Krt31	2.78	1.06	2.61	5.485964917e-05	0.00	0.01	0.00	0.00	0.00	0.00
AW146154	0.56	0.21	2.60	0.044111456	0.22	0.14	0.23	0.19	0.37	0.36
Rpl17-ps3	17.57	6.81	2.58	1.166739003e-11	1.05	0.73	9.02	1.55	11.51	3.27
Igh-Ia	0.88	0.34	2.54	0.024413996	0.00	0.00	0.00	0.00	0.00	0.00
CR974428.1	0.72	0.28	2.54	0.034303955	0.00	0.00	0.00	0.00	0.00	0.00
AC155933.1	0.60	0.23	2.54	0.043675142	1.12	0.88	2.86	3.05	0.53	0.21
AC134590.1	0.58	0.23	2.54	0.044963803	0.00	0.00	0.00	0.08	0.28	0.00
Hist2h3b	0.56	0.22	2.54	0.046307506	0.15	0.08	0.20	0.00	1.96	13.41
Gm9853	3.09	1.21	2.54	2.970426396e-05	1.33	2.54	16.81	13.96	11.23	9.39
AC132287.4	2.03	0.80	2.54	0.000416416	0.00	0.00	0.00	0.00	0.64	0.73
AC158956.1	0.88	0.35	2.54	0.024302102	1.60	1.69	1.41	2.13	0.00	0.00
Cd28	2.06	0.82	2.52	0.000424985	0.00	0.01	0.43	0.35	0.00	0.00
RP23-14F5.5	3.06	1.22	2.52	3.583459449e-05	1.02	1.26	0.90	1.04	2.54	0.54
B4galnt3	0.83	0.34	2.45	0.031380824	0.16	0.18	0.03	0.03	0.00	0.00
Npas2	0.59	0.24	2.44	0.049948454	4.82	5.02	0.59	0.66	0.17	0.23
Lrrc26	3.73	1.57	2.38	9.300536209e-06	0.93	0.85	0.00	0.00	0.00	0.00
Clcnka	1.64	0.70	2.35	0.001846237	0.01	0.00	0.00	0.00	0.06	0.01
RP23-226O20.7	0.88	0.38	2.33	0.03363932	0.03	0.05	1.02	0.40	0.23	0.78
AC124479.2	0.81	0.35	2.33	0.038934391	0.00	0.00	0.08	0.12	1.12	0.32
Slpi	1.32	0.57	2.31	0.009948644	0.00	0.01	0.19	0.22	59.55	59.35
Ckmt2	0.78	0.34	2.31	0.043083114	0.01	0.00	0.03	0.00	0.00	0.00
Rps8-ps1	3.01	1.31	2.31	0.000117201	0.20	0.62	0.39	0.35	5.14	6.94
RP23-475N16.2	2.54	1.10	2.30	0.000382208	0.09	0.04	2.95	2.20	0.89	1.72
Serpinb9e	1.57	0.69	2.28	0.003569895	0.00	0.00	0.00	0.00	0.00	0.00
AC153851.12	0.83	0.37	2.26	0.042483	0.00	0.00	0.00	0.00	0.00	0.00
Lgals4	1.36	0.60	2.25	0.010144749	0.48	0.28	0.69	0.58	0.59	0.83
Neu2	3.39	1.51	2.25	5.399674145e-05	0.10	0.19	0.23	0.32	0.07	0.05

RP23-196B19.3	1.02	0.46	2.23	0.029664235	0.68	0.40	0.72	1.16	0.37	0.31
RP23-176J12.2	1.77	0.80	2.20	0.002201234	0.01	0.00	0.03	0.02	0.72	0.32
RP23-265A5.1	1.43	0.65	2.20	0.008610836	0.00	0.00	0.00	0.00	0.00	0.00
Gm3776	1.16	0.53	2.20	0.021460138	0.03	0.05	0.05	0.00	0.04	0.00
Hist1h1e	2.27	1.03	2.19	0.001036481	0.18	0.16	2.94	2.67	4.31	7.48
Nctc1	0.99	0.45	2.19	0.034415499	0.01	0.00	0.00	0.00	0.00	0.00
D830039M14Rik	1.21	0.56	2.18	0.01981735	0.15	0.20	0.07	0.06	0.13	0.14
Lctl	1.94	0.89	2.18	0.001905864	0.07	0.01	0.67	0.46	0.03	0.02
RP23-8O24.1	1.13	0.53	2.16	0.025932281	0.00	0.01	0.02	0.03	0.00	0.00
Slc44a4	15.80	7.41	2.13	7.022927094e-09	0.03	0.01	0.02	0.00	0.00	0.04
Gm10726	1.24	0.58	2.12	0.021420978	0.48	0.29	1.56	2.03	1.17	0.80
RP23-294E23.8	1.05	0.49	2.12	0.035143362	0.01	0.01	0.00	0.00	0.00	0.00
Cd24a	3.45	1.63	2.11	0.000107979	220.19	228.67	114.77	118.15	17.60	12.07
AC151989.1	0.93	0.44	2.08	0.049001015	0.33	0.27	0.12	0.06	0.27	0.22
AC101915.1	2.44	1.17	2.08	0.001296792	0.73	0.95	3.25	3.95	0.95	0.97
Ppil6	1.01	0.49	2.08	0.041293039	4.20	3.50	3.28	3.45	0.00	0.08
Pigc	7.32	3.56	2.06	1.517745859e-06	0.23	0.54	4.39	3.38	1.93	2.68
AC153821.1	1.65	0.82	2.03	0.005752238	0.76	0.63	2.53	1.25	1.35	1.13
AC159539.1	0.93	0.00	n.d.	0.000149605	0.65	0.31	0.98	0.64	0.74	0.33
AC107663.1	0.80	0.00	n.d.	0.000317683	0.00	0.47	1.69	0.00	0.76	0.86
AL807745.2	0.80	0.00	n.d.	0.000317683	0.00	0.00	0.00	0.00	0.00	0.00
Hist1h4m	0.72	0.00	n.d.	0.00050046	0.12	0.08	2.36	3.62	6.22	14.55
CT030194.3	0.45	0.00	n.d.	0.005652393	0.24	0.18	0.48	0.25	0.29	0.16
RP23-377K9.1	0.44	0.00	n.d.	0.00772481	0.00	0.03	0.55	0.07	0.00	0.00
Npff	0.44	0.00	n.d.	0.007862985	0.54	0.68	1.10	1.65	0.83	0.47
Rpl37-ps1	0.43	0.00	n.d.	0.009001285	0.00	0.00	0.15	0.12	1.09	1.38
AL807396.1	0.40	0.00	n.d.	0.013700036	0.00	0.06	0.21	0.17	0.38	0.22
Gm9983	0.36	0.00	n.d.	0.022416633	0.00	0.04	0.13	0.00	0.00	0.00
AC102121.1	0.35	0.00	n.d.	0.026920719	0.04	0.00	0.37	0.29	0.55	0.25
4930547M16Rik	0.34	0.00	n.d.	0.027821358	0.28	0.45	0.36	0.42	0.16	0.00
RP23-133J9.5	0.34	0.00	n.d.	0.028421079	0.52	0.30	0.12	0.09	0.33	0.49
Gm11127	0.34	0.00	n.d.	0.029618155	0.00	0.00	0.00	0.00	0.00	0.00
AC116763.1	0.34	0.00	n.d.	0.029618155	0.00	0.00	0.71	0.00	1.29	0.36
RP23-428I4.3	0.33	0.00	n.d.	0.031851145	0.08	0.00	0.26	0.14	0.47	0.98
AC159308.2	0.33	0.00	n.d.	0.033623357	0.00	0.05	0.00	0.13	0.00	0.00
2810474C18Rik	0.32	0.00	n.d.	0.034767176	0.44	0.35	0.68	0.21	0.12	0.21
RP23-391E6.1	0.32	0.00	n.d.	0.036165094	0.23	0.15	0.07	0.05	0.06	0.14
Gm9998	0.31	0.00	n.d.	0.038411122	0.44	0.30	0.33	0.17	0.00	0.22
Gm10494	0.31	0.00	n.d.	0.040115346	0.00	0.00	0.00	0.00	0.00	0.00
Mpp7 (18 7348765..7348895)	0.31	0.00	n.d.	0.040115346	0.50	0.18	1.94	3.02	1.74	1.32
AC113015.1	0.30	0.00	n.d.	0.042630289	0.00	0.00	0.00	0.25	1.14	0.64
AC174776.2	0.29	0.00	n.d.	0.044277873	0.10	0.17	0.00	0.00	0.00	0.00
AC154265.2	0.29	0.00	n.d.	0.045092653	0.00	0.00	0.15	0.24	0.14	0.00
Rps24-ps2	2.13	0.00	n.d.	3.626642438e-09	0.16	0.08	0.92	0.48	0.18	0.83
AC187103.1	2.80	0.00	n.d.	5.69582249e-11	0.18	0.00	0.00	0.00	2.92	1.80
AL645845.1	1.29	0.00	n.d.	8.084561321e-06	0.42	0.75	2.03	0.00	1.22	0.00

[a]Data represent RPKM values if not specified otherwise
[b]not determined

Supplementary Table S3: Expression of host cell genes regulated by *T. gondii* in neurons (at least 2-fold; $p < 0.05$)
A: Genes up-regulated by *T. gondii*

	Neurons				SkMCs		Astrocytes		Fibroblasts	
**Gene ID**	**n.i.**[a]	***T. gondii***	**fold increase**	***p* value**	**n.i.**	***T. gondii***	**n.i.**	***T. gondii***	**n.i.**	***T. gondii***
Gm10260	0.03	9.21	338.58	8.18686199e-23	440.43	568.04	0.44	2.28	1340.18	1540.92
Car6	0.01	2.03	147.34	7.744853882e-09	0.23	0.40	0.02	0.07	0.99	1.12
Gbp5	0.01	0.40	57.77	0.015137962	0.00	0.01	1.34	2.17	0.00	0.01
Pla2g2e	0.01	0.73	53.74	0.000593492	1.72	1.49	0.04	0.21	0.04	0.14
Ccl2	0.08	3.93	51.18	3.167777302e-14	2.17	6.40	12.86	17.84	30.19	38.37
Rpl39l	0.02	0.74	49.26	0.000581797	0.09	1.04	0.69	1.03	132.59	154.24
Pnma5	0.02	0.93	46.58	0.000202264	0.00	0.15	0.00	0.01	0.11	0.23
Hmga1-rs1	0.06	2.40	39.08	2.056123302e-09	26.36	47.01	0.30	0.88	252.34	305.12
Hoxc10	0.01	0.51	38.96	0.001716138	29.54	28.10	0.00	0.03	0.00	0.09
Ankrd1	0.01	0.48	34.04	0.003948548	708.58	643.91	0.14	0.50	26.38	22.42
Ptgir	0.01	0.38	25.53	0.022857447	0.99	1.20	0.60	0.43	1.48	1.74
Plaur	0.02	0.33	18.81	0.041106057	5.48	6.82	6.30	5.82	49.96	56.03
Mlkl	0.02	0.37	17.02	0.029933901	5.24	5.10	1.80	2.70	25.69	22.95
S100a4	0.66	8.76	13.29	2.120529922e-19	1494.26	1538.80	115.89	102.69	3652.98	3581.42
1700012B09Rik	0.06	0.63	11.35	0.002178048	1.93	1.54	1.20	1.87	0.00	0.31
Hmga2	0.05	0.56	10.50	0.002983335	1.61	1.78	0.59	0.83	36.01	28.01
Timp1	0.10	0.97	9.85	0.000528512	309.92	350.60	26.28	24.62	169.25	184.00
AC167245.5	0.07	0.60	9.18	0.002978989	0.00	0.00	1.93	1.42	0.24	0.22
Cxcl1	0.12	0.99	8.36	0.00062745	0.20	1.19	0.60	1.13	1.23	1.43
Cxcl10	0.33	2.72	8.29	1.25245828e-08	0.00	0.09	3.21	9.62	0.10	0.03
Hist2h2ac	0.07	0.54	7.46	0.004835995	0.52	1.75	0.70	0.43	4.02	9.16
Gm10119	0.13	0.91	7.28	0.001153781	3.30	9.79	1.33	0.44	0.32	0.26
Aqp1	0.08	0.53	6.94	0.005518207	15.96	17.74	9.16	8.26	92.50	111.29
Anxa3	0.06	0.39	6.91	0.039192924	162.18	132.03	252.86	235.08	100.21	83.04
RP23-84O5.1	0.07	0.44	6.72	0.021734895	0.00	0.00	0.00	0.00	0.00	0.00
Gm7589	1.05	6.69	6.39	7.923285876e-15	11.64	11.19	7.57	4.73	5.93	22.83
Capg	0.10	0.64	6.18	0.004599035	60.67	65.26	90.77	83.21	64.15	70.54
AC112998.1	0.11	0.64	5.73	0.005314975	1.31	1.30	0.65	0.68	2.15	3.03
RP23-452C10.2	0.15	0.82	5.55	0.002921587	3.57	4.32	0.19	0.15	1.65	2.66
Gm6166	0.22	1.21	5.45	0.000436524	13.47	32.25	0.54	0.47	237.86	264.75
Angptl4	0.19	1.05	5.41	0.001140191	15.49	27.03	2.22	2.53	47.34	50.01
Ost4	0.11	0.59	5.37	0.006975265	2.03	1.20	1.07	1.95	0.96	2.91
Igfbp6	0.30	1.63	5.37	1.185852592e-05	95.70	99.11	6.55	7.96	651.06	751.72
Tnnt2	0.13	0.69	5.08	0.005779399	1397.94	1183.85	0.33	0.49	7.66	7.28
Psmb8	0.10	0.48	5.04	0.018271985	0.18	0.82	49.53	55.29	0.03	0.12
AC102562.1	0.09	0.44	4.86	0.032732586	11.34	15.31	0.46	0.26	23.28	20.69
Ostn	0.25	1.19	4.83	0.000743198	0.00	0.00	0.06	0.02	0.00	0.03
Zc3hav1	0.11	0.54	4.67	0.010565459	8.23	8.89	10.20	12.26	12.09	10.63
Akr1b8	0.09	0.42	4.56	0.046432016	66.21	63.72	13.86	12.25	53.64	49.50
Adm2	0.10	0.43	4.48	0.041384557	1.04	1.18	0.04	0.03	0.04	0.08
Aebp1	0.37	1.64	4.40	2.807487508e-05	110.82	96.10	77.44	83.34	224.17	222.01
Pcolce	0.23	1.02	4.38	0.002402807	87.82	96.93	18.50	17.56	119.00	127.54
Has2	0.14	0.59	4.28	0.011136275	11.20	18.39	0.29	0.31	24.23	18.51
CR974466.4	0.25	1.07	4.25	0.002010208	3.96	7.10	28.15	32.78	2.47	3.33
Pla2g5	0.28	1.14	4.04	0.001775618	0.00	0.16	3.81	3.44	0.00	0.22
2510046G10Rik	0.14	0.58	4.03	0.013023613	0.00	0.39	0.23	0.55	0.11	0.95
Gm1943	0.19	0.76	4.03	0.007736674	6.72	8.96	5.39	3.61	11.24	13.34
AC150274.1	0.74	2.97	4.02	3.951898191e-07	311.43	440.08	1.18	1.59	570.81	621.64
Serpinf1	0.52	2.03	3.92	1.465523173e-05	122.77	139.61	43.62	43.77	307.29	318.01
Crabp2	0.13	0.52	3.90	0.016343646	52.34	47.74	0.98	1.73	0.42	0.53
Col1a2	0.21	0.78	3.80	0.008509362	77.76	77.36	26.39	29.60	431.07	401.12
Anxa1	0.16	0.60	3.73	0.014847411	297.25	312.90	131.68	123.55	358.01	342.60
AC160404.2	0.14	0.53	3.69	0.01801085	1.44	0.85	1.40	0.77	1.21	1.48
RP23-252K7.2	0.25	0.92	3.68	0.005842574	0.17	0.20	0.09	0.14	0.08	0.00
Tuba1c	3.58	13.10	3.66	9.245950575e-15	207.17	304.49	98.39	97.02	357.68	425.70
AC131919.1	0.44	1.55	3.53	0.000228658	0.56	0.94	0.84	0.20	0.68	0.77
Gm10595	0.70	2.36	3.36	1.75125528e-05	0.00	0.00	0.00	0.00	0.00	0.00
AC139673.1	0.18	0.57	3.25	0.022611444	0.71	0.32	1.04	1.43	1.32	1.02
AC102115.2	0.92	2.98	3.23	3.111478964e-06	10.38	5.98	9.77	3.66	5.75	4.38
Cd74	0.29	0.93	3.20	0.008979515	0.25	1.13	1.70	1.89	0.21	0.76
Cav1	0.15	0.49	3.20	0.038273531	37.90	41.09	20.15	22.52	115.91	97.75
Gbp3	0.20	0.65	3.16	0.020630766	0.10	0.35	11.20	14.36	0.23	0.20
Lgals1	8.79	27.59	3.14	7.345377214e-15	5750.50	6012.51	1017.07	1071.99	8188.80	9416.11
Rps8-ps1	0.20	0.62	3.13	0.022162824	3.01	1.31	0.39	0.35	5.14	6.94
Arpc1b	0.59	1.85	3.11	0.000119916	56.18	69.25	75.80	79.71	95.56	111.42
Flnc	0.18	0.55	3.10	0.026673657	166.49	133.79	19.34	20.85	34.47	32.99
Apobec3	0.25	0.79	3.10	0.015432	0.64	3.13	2.22	2.91	4.31	4.08
Fkbp11	0.70	2.13	3.06	7.038794748e-05	11.51	17.42	4.32	3.50	32.44	41.54
Avil	0.23	0.70	3.04	0.02009341	0.09	0.19	0.11	0.15	0.06	0.12
Rgs13	0.18	0.55	3.02	0.028635443	0.00	0.00	0.00	0.00	0.00	0.00
Glycam1	0.80	2.42	3.02	3.694085965e-05	0.00	0.00	7.03	6.55	0.00	0.00
S100a11	35.06	105.71	3.01	2.367231241e-14	651.35	621.28	799.76	827.58	750.18	794.41
Gm9952	1.01	2.90	2.87	1.439912502e-05	0.00	0.29	0.00	0.10	0.00	0.13
Atf3	0.27	0.77	2.85	0.020899568	3.83	7.13	21.03	15.78	1.37	1.75
Chaf1b	1.13	3.13	2.77	9.470793994e-06	0.74	2.05	2.27	2.21	4.94	5.40
Emp1	0.42	1.13	2.69	0.009153286	102.47	103.81	50.50	50.77	186.58	152.49
S100a6	1.48	3.97	2.69	1.108222837e-06	541.94	619.76	337.44	253.84	1912.45	1972.31
Ifitm3	0.99	2.66	2.69	5.659198947e-05	435.30	456.28	749.28	737.08	508.02	539.76
AC171204.1	0.69	1.78	2.60	0.000543102	24.91	30.64	0.52	0.55	19.13	19.89
Fam150b	0.31	0.81	2.57	0.027605913	0.05	0.03	0.08	0.02	0.07	0.05
Fosb	0.32	0.82	2.56	0.027532375	0.27	4.83	0.27	0.41	0.67	0.70
Csf1	2.09	5.26	2.51	4.194122232e-07	31.32	33.01	66.55	69.91	84.09	75.19
Neurod1	0.82	2.07	2.51	0.000427641	0.00	0.00	0.00	0.00	0.00	0.00
Baz1a	0.38	0.95	2.47	0.022656496	8.89	11.96	4.40	4.87	6.61	6.22
Peg12	0.37	0.91	2.47	0.025380475	0.27	0.37	1.00	1.34	2.45	4.77
Fosl1	1.64	4.00	2.44	4.351375869e-06	5.45	10.70	2.18	2.84	50.06	57.30
Slc1a5	0.37	0.89	2.39	0.029978052	2.99	3.13	2.67	3.53	30.68	36.36

Ptrf	0.81	1.92	2.38	0.00088848	78.47	80.85	63.24	62.20	101.51	95.29
Cdt1	1.85	4.41	2.38	3.87794295e-06	2.89	6.42	9.23	10.00	37.74	42.11
Fhl3	0.43	1.01	2.35	0.02425393	32.04	36.93	14.30	14.46	65.43	71.90
Syngr2	0.35	0.81	2.31	0.040731299	35.64	40.88	24.67	22.14	17.62	21.59
AC138299.1	0.48	1.10	2.30	0.020857551	4.03	3.05	2.16	1.41	2.68	3.36
Nfkb2	0.67	1.52	2.27	0.004684082	39.31	60.89	16.89	20.04	31.40	41.17
Wdhd1	0.66	1.48	2.25	0.005997702	0.62	1.71	1.95	2.35	3.53	3.73
Cdc25c	0.48	1.09	2.25	0.024300862	0.96	2.21	2.29	2.51	10.37	9.46
Cnn2	0.49	1.05	2.16	0.031243896	94.85	83.00	182.23	166.59	194.16	192.38
Trim47	0.55	1.19	2.15	0.022460231	19.17	24.39	7.05	7.90	12.75	20.15
Gmnn	1.19	2.54	2.14	0.000810908	6.07	7.65	13.82	13.93	24.31	25.78
Efemp1	0.97	2.05	2.12	0.00204378	0.49	0.60	34.83	37.89	239.35	192.94
Plp2	1.27	2.69	2.11	0.000687951	138.10	156.12	73.40	78.77	252.27	247.72
Sod3	1.40	2.93	2.09	0.000490131	48.34	62.31	34.26	32.89	42.73	45.14
Fstl3	0.86	1.79	2.07	0.003679167	31.86	33.95	3.31	3.23	22.49	25.08
Magmas	3.96	8.17	2.06	8.064012511e-07	5.49	7.43	3.13	2.81	4.15	6.33
RP23-33N15.2	0.49	1.00	2.04	0.046714282	0.13	0.15	0.26	0.41	0.36	0.18
Mybl2	1.28	2.62	2.04	0.001176945	0.42	1.59	1.66	2.20	4.72	5.99
Fadd	0.53	1.08	2.04	0.038756335	2.44	2.20	3.82	4.10	3.05	3.31
Cdkn3	0.53	1.08	2.04	0.03935142	0.73	2.59	6.47	6.34	4.81	5.44
Gadd45b	1.23	2.47	2.01	0.001826435	38.91	58.42	20.25	23.23	63.77	79.86
Ccne2	0.99	1.99	2.00	0.003835481	0.72	2.07	1.42	2.10	2.02	1.93
AC139320.1	0.00	0.75	n.d.[b]	0.000433765	0.05	0.18	0.00	0.20	0.00	0.32
Fabp4	0.00	0.60	n.d.	0.000884094	0.00	0.29	11.36	6.23	0.58	0.85
Tmem171	0.00	0.49	n.d.	0.002201945	0.19	0.52	2.93	2.22	1.23	1.07
AC107663.1	0.00	0.47	n.d.	0.00438173	0.80	0.00	1.69	0.00	0.76	0.86
Tpsab1	0.00	0.42	n.d.	0.010088634	0.07	0.07	0.00	0.02	0.00	0.04
RP23-282N17.6	0.00	0.37	n.d.	0.02085095	0.06	0.48	0.06	0.00	0.44	0.50
AC125254.2	0.00	0.34	n.d.	0.029451729	0.00	0.00	0.00	0.00	0.00	0.00
Rpl27a-ps2	0.00	0.30	n.d.	0.042907523	0.00	0.00	0.09	0.14	0.00	0.00
AL591712.1	0.00	0.29	n.d.	0.047108989	0.00	0.00	0.00	0.00	0.00	0.00

B: *Genes down-regulated by T. gondii*

	Neurons				SkMCs		Astrocytes		Fibroblasts	
**Gene ID**	**n.i.**	***T. gondii***	**fold decrease**	***p* value**	**n.i.**	***T. gondii***	**n.i.**	***T. gondii***	**n.i.**	***T. gondii***
Gm10719	0.31	0.02	18.98	0.049584771	0.06	0.13	0.12	0.05	0.05	0.06
AL837506.1	9.08	0.92	9.86	1.047845432e-18	0.61	1.16	3.75	1.93	2.08	1.30
Gm5528	0.44	0.05	9.49	0.016629053	0.00	0.38	0.00	0.85	0.83	0.26
Gm10800	40.14	4.78	8.40	8.595789037e-24	1.58	1.31	10.23	4.42	6.94	4.27
Gm10789	0.48	0.06	7.82	0.010563375	0.00	0.00	0.00	0.00	0.00	0.00
Gstp2	0.58	0.08	7.15	0.00442445	2.42	2.46	0.89	0.05	1.07	1.15
AC154013.1	0.42	0.07	6.33	0.031729319	0.30	0.27	0.24	0.12	12.19	9.87
AC122451.1	2.86	0.47	6.14	2.934767096e-08	0.00	0.00	0.00	0.00	0.00	0.00
Pcdha6	1.78	0.31	5.81	3.399186546e-06	0.01	0.03	0.35	0.33	0.26	0.34
Nap1l5	0.41	0.08	4.84	0.048114218	0.00	0.00	0.05	0.00	0.00	0.00
Gm10710	0.50	0.11	4.69	0.014695244	0.00	0.00	0.00	0.06	0.00	0.00
RP23-11O22.5	0.56	0.13	4.28	0.011918414	0.08	0.09	0.32	0.06	0.14	0.16
AC125099.7	0.48	0.12	4.19	0.023791783	0.00	0.23	0.63	0.25	0.94	0.21
Rpl21-ps11	0.48	0.12	4.02	0.026621098	0.00	0.00	0.09	0.27	0.63	0.80
CT027556.2	1.34	0.36	3.76	0.000778486	2.62	0.59	0.88	0.14	2.23	1.44
AC121926.1	5.56	1.55	3.58	6.618049725e-10	1.26	1.05	1.10	0.67	0.71	0.56
Gm5830	4.52	1.27	3.57	7.878161274e-09	18.68	53.80	10.61	23.84	12.90	12.77
Gm10362	0.81	0.24	3.35	0.011345304	5.98	3.69	1.54	0.86	0.79	0.37
Gm6354	0.58	0.17	3.35	0.020635738	0.00	0.00	0.21	0.16	0.19	0.42
AC159815.1	2.81	0.93	3.04	1.039180729e-05	32.63	25.94	22.25	8.90	12.46	16.48
Gm16382	0.59	0.21	2.87	0.030392965	5.82	4.82	3.44	2.01	1.07	2.96
Mpp7 (18 7348765..7348895)	0.50	0.18	2.79	0.048901841	0.31	0.00	1.94	3.02	1.74	1.32
RP23-317F9.1	2.50	0.92	2.73	7.612317975e-05	0.00	0.00	2.22	2.31	0.00	0.00
RP23-251B16.1	1.75	0.66	2.64	0.00051595	0.00	0.00	0.00	0.51	0.20	0.00
Gm9790	0.55	0.21	2.61	0.043890811	1.30	0.38	1.20	1.67	0.85	2.01
C330021F23Rik	1.18	0.47	2.49	0.011343607	0.47	0.67	2.70	1.57	6.74	8.75
C1qc	0.78	0.32	2.41	0.036603169	0.02	0.00	307.97	314.70	0.00	0.00
1700029I15Rik	0.70	0.30	2.36	0.046240957	0.06	0.49	0.38	0.49	0.34	0.06
Hist1h2ag	0.75	0.32	2.31	0.045723675	0.48	1.78	1.59	0.72	6.65	16.66
Ccl3	1.47	0.70	2.09	0.010416161	0.05	0.00	25.69	16.98	0.00	0.05
RP23-384C18.7	1.53	0.75	2.04	0.009464885	11.81	12.18	2.24	2.49	3.54	2.82
Gm10288	5.14	2.52	2.04	2.14713895e-05	3.95	5.51	4.77	2.42	5.18	6.94
AC167249.3	33.13	16.31	2.03	6.834684023e-09	58.36	21.83	70.50	62.02	89.71	31.29
RP23-321D1.4	0.95	0.00	n.d.	0.000129684	0.73	6.50	6.58	2.42	1.40	1.58
AL732624.2	0.79	0.00	n.d.	0.000337218	0.00	0.00	0.00	0.00	0.00	0.00
BX294391.1	0.76	0.00	n.d.	0.000409216	0.00	0.00	0.00	0.00	0.00	0.00
AC149283.1	0.59	0.00	n.d.	0.000893355	0.00	0.00	0.00	0.00	0.00	0.00
AL589661.1	0.31	0.00	n.d.	0.038040547	0.00	0.00	0.00	0.00	0.00	0.00
AC102363.1	1.44	0.00	n.d.	1.405852068e-06	0.00	0.00	0.00	0.28	0.00	0.00

[a]Data represent RPKM values if not specified otherwise
[b]not determined

Supplementary Table S4: Expression of host cell genes regulated by *T. gondii* in astrocytes (at least 2-fold; $p < 0.05$)
A: Genes up-regulated by *T. gondii*

	Astrocytes				SkMCs		Neurons		Fibroblasts	
**Gene ID**	**n.i.[a]**	***T. gondii***	**fold increase**	***p* value**	**n.i.**	***T. gondii***	**n.i.**	***T. gondii***	**n.i.**	***T. gondii***
E230019M04Rik	0.03	0.34	12.48	0.046191293	0.00	0.00	0.01	0.01	0.00	0.00
BC049762	0.04	0.36	9.36	0.044805114	0.00	0.04	0.05	0.05	0.03	0.00
Rpl21-ps8	0.08	0.53	7.02	0.005446529	0.21	0.59	0.14	0.17	1.82	1.30
AW557046	0.07	0.44	6.24	0.02296312	0.07	0.08	1.25	1.02	0.26	0.29
Prl2c3	0.24	1.49	6.24	2.940033789e-05	7.08	10.22	0.00	0.00	3.58	2.49
4933416I08Rik	0.10	0.61	6.24	0.004956308	0.00	0.39	1.93	1.85	0.00	0.25
Rtn4r	0.06	0.39	6.24	0.04137225	0.28	0.54	30.33	30.54	0.09	0.09
AC158913.1	0.16	0.86	5.46	0.002610742	0.67	0.53	0.19	0.11	0.99	1.61
Gm5745	0.13	0.70	5.46	0.004663288	0.24	0.29	0.12	0.04	0.70	1.32
Gm10260	0.44	2.28	5.15	1.138191192e-06	440.43	568.04	0.03	9.21	1340.18	1540.92
Gm10817	0.10	0.48	4.94	0.019460391	0.27	0.22	0.67	0.61	0.09	0.07
Glyctk	0.10	0.47	4.92	0.021071028	0.16	0.16	0.11	0.26	0.21	0.23
Hist1h2bh	0.20	0.92	4.68	0.002956987	0.00	0.55	0.00	0.00	3.81	6.22
Vnn3	0.13	0.63	4.68	0.008101451	0.00	0.00	0.00	0.00	0.00	0.00
Gm4392	0.12	0.56	4.68	0.009853673	0.00	0.00	2.76	2.74	0.00	0.00
RP24-114K21.6	0.11	0.54	4.68	0.010549532	0.00	0.00	0.00	0.00	0.00	0.00
AC144914.1	0.17	0.74	4.47	0.006448048	2.62	1.95	0.00	0.00	0.09	0.05
AC133589.1	0.57	2.51	4.43	1.281807475e-06	0.42	1.31	0.30	0.44	0.71	0.58
Gm8394	0.17	0.69	4.10	0.009231367	0.44	0.94	0.01	0.01	1.94	0.47
Trex1	1.24	4.99	4.02	5.390368852e-10	0.94	1.24	1.40	2.27	1.28	1.31
Gm11627	0.14	0.55	3.90	0.01518949	0.30	0.16	0.70	0.46	0.28	0.21
Rpl41 (10 43176227..43176309)	0.52	2.03	3.90	1.504732597e-05	1.48	1.75	0.16	0.14	4.23	3.19
Gm9905	0.12	0.48	3.90	0.029735341	0.06	0.14	0.04	0.02	0.00	0.00
Ankrd1	0.14	0.50	3.64	0.022637456	708.58	643.91	0.01	0.48	26.38	22.42
Gm10353	0.21	0.72	3.38	0.014161779	0.00	0.16	0.57	0.39	0.26	0.58
RP23-186O3.2	0.16	0.55	3.38	0.021361891	0.05	0.00	0.73	0.61	0.10	0.39
Gm10269	2.40	8.02	3.33	2.388294448e-11	17.21	27.52	6.38	4.33	16.25	18.71
9430025M13Rik	0.28	0.91	3.23	0.009366599	0.65	0.23	2.05	2.26	0.33	0.25
RP23-77H16.5	0.23	0.71	3.12	0.018225124	0.76	0.64	0.32	0.19	0.31	0.12
AC102874.2	0.62	1.92	3.12	9.953363358e-05	0.00	0.00	0.00	0.00	0.00	0.00
AC122408.1	0.38	1.19	3.12	0.003855736	0.84	1.56	0.62	0.49	1.48	2.84
Gm5121	0.35	1.08	3.12	0.005860325	3.18	5.95	0.36	0.14	27.67	16.32
AC154200.2	0.18	0.56	3.12	0.025988287	0.73	0.20	0.07	0.08	0.43	0.30
Zfp322a	0.99	3.06	3.08	3.598388022e-06	4.05	2.28	0.93	1.50	1.13	0.64
Hist1h2ad	0.64	1.94	3.03	0.000118324	0.88	0.64	0.11	0.18	3.27	1.45
Cxcl10	3.21	9.62	3.00	2.971736744e-11	0.00	0.09	0.33	2.72	0.10	0.03
RP24-258K6.2	0.35	1.04	2.99	0.008124302	0.27	0.65	0.62	0.43	0.05	0.24
Hmga1-rs1	0.30	0.88	2.93	0.014575332	26.36	47.01	0.06	2.40	252.34	305.12
RP24-103E20.2	0.43	1.23	2.86	0.004669256	3.41	0.32	1.32	0.99	1.29	1.76
RP23-138F20.17	0.85	2.31	2.73	0.000121243	0.08	0.09	0.03	0.09	0.08	0.00
Gm16378	0.37	1.00	2.73	0.01335508	1.67	2.18	0.41	0.15	0.60	1.18
Cxcl2	0.30	0.82	2.73	0.021479874	0.00	0.00	0.01	0.02	0.00	0.00
RP23-109A3.7	0.21	0.56	2.73	0.037361399	0.10	0.23	0.03	0.00	0.37	0.32
AC113988.2	0.20	0.54	2.73	0.038717798	1.13	0.78	0.03	0.03	1.26	1.42
Gm6377	0.19	0.51	2.70	0.04408821	0.00	0.02	0.00	0.00	0.01	0.03
C330006D17Rik	0.54	1.43	2.65	0.00278841	1.02	1.69	0.99	0.95	0.58	1.10
Tnnt3	0.24	0.64	2.63	0.036323452	1337.06	1098.76	0.26	0.27	0.46	0.46
Ccdc24	0.32	0.83	2.60	0.025222044	0.27	0.40	0.98	1.01	0.86	1.18
Gm5457	0.25	0.66	2.60	0.036255117	0.32	0.19	0.08	0.19	0.08	0.00
RP23-56A7.3	0.93	2.41	2.57	0.000166173	3.81	3.35	0.11	0.10	7.49	3.43
AC153534.1	0.52	1.34	2.56	0.005232229	0.35	0.67	0.00	0.04	0.00	0.08
Fam26f	0.41	1.05	2.55	0.015176851	0.00	0.00	0.00	0.11	0.10	0.11
AC127300.1	0.52	1.30	2.50	0.007004532	1.78	1.63	0.46	0.92	1.88	2.44
Gm10703	0.92	2.28	2.47	0.000323173	0.00	0.00	0.09	0.00	0.00	0.31
AC116387.1	0.64	1.57	2.47	0.001980996	0.00	0.00	4.16	3.64	0.00	0.00
AC122248.2 (7 128090547..128090944)	0.72	1.78	2.45	0.000897105	0.20	0.58	3.59	2.85	1.40	0.53
Adnp	8.94	21.55	2.41	2.372334914e-11	15.31	14.20	19.10	16.45	11.37	6.68
Gm9766	0.42	1.01	2.40	0.021931971	1.03	1.52	2.96	2.75	1.09	0.99
Gm8325	1.95	4.64	2.38	2.80791359e-06	19.00	26.89	0.55	0.54	2.55	3.08
CAAA01141682.1.67857.1	0.78	1.84	2.34	0.001194561	2.23	1.23	0.24	0.56	0.85	1.28
AC161053.2	0.48	1.13	2.34	0.017500138	0.92	1.63	2.82	2.00	0.44	0.00
Gm7367	0.46	1.09	2.34	0.020064304	0.56	0.31	0.28	0.17	0.42	0.66
AC149589.1	0.39	0.91	2.34	0.031235638	1.03	0.61	0.36	0.30	0.28	0.55
AC182749.3	0.39	0.91	2.34	0.031575426	0.59	0.57	0.23	0.20	0.46	0.64
F13a1	0.63	1.45	2.29	0.006275761	0.24	0.23	0.01	0.03	0.01	0.01
Gm5830	10.61	23.84	2.25	2.381470575e-10	18.68	53.80	4.52	1.27	12.90	12.77
AC172027.3	0.89	1.99	2.24	0.001343343	1.79	0.93	1.01	1.50	1.50	0.91
Stra6	0.57	1.24	2.18	0.018382468	0.11	0.13	0.05	0.09	0.86	1.47
AC155074.1	0.71	1.54	2.16	0.006091043	0.31	0.12	0.07	0.02	0.00	0.06
1700048O20Rik	0.62	1.31	2.12	0.016692033	0.00	0.03	2.44	2.60	4.04	3.51
Usp31 (7 128785943..128786166)	1.30	2.75	2.12	0.000595244	3.35	3.12	6.78	6.38	3.35	2.84
AC124505.11	0.51	1.07	2.11	0.034053592	0.48	0.57	0.58	0.78	1.79	3.01
Dynlt1-ps1	51.76	109.01	2.11	2.035084843e-09	103.41	116.34	8.37	7.06	106.91	156.60
B3gnt7	0.68	1.42	2.10	0.01223067	0.00	0.02	6.47	5.54	0.00	0.00
Gjb2	0.51	1.05	2.08	0.037577273	0.37	0.42	0.25	0.22	0.00	0.00
RP24-188M1.3	6.43	13.33	2.07	3.225034501e-08	13.89	9.99	23.45	18.81	52.88	70.72
Gm5805	0.87	1.79	2.06	0.003903057	1.80	0.44	0.29	0.35	3.27	1.69
CT030166.3	4.10	8.44	2.06	7.034198149e-07	10.73	13.78	0.11	0.17	19.38	15.46
Srgn	10.48	21.55	2.06	4.486276766e-09	0.25	0.41	0.04	0.08	0.07	0.02
D130020L05Rik	0.66	1.34	2.03	0.019936454	0.40	0.45	0.89	0.83	0.13	0.12
Lass1	3.12	6.31	2.02	5.309419152e-06	1.42	1.10	25.99	25.66	0.37	0.30
Gm6020	0.00	0.96	n.d.[b]	0.00012129	0.00	0.00	0.00	0.00	0.00	0.00
Gm5528	0.00	0.85	n.d.	0.000238302	0.00	0.38	0.44	0.05	0.83	0.26
AL929042.1	0.00	0.66	n.d.	0.000659924	0.80	1.18	0.07	0.06	2.48	2.80
AC132287.1	0.00	0.62	n.d.	0.000790111	1.51	0.89	0.00	0.00	0.00	0.00
AL845323.1	0.00	0.59	n.d.	0.000921521	0.00	0.00	0.00	0.00	0.00	0.00
AC184160.2	0.00	0.53	n.d.	0.001182086	0.00	0.00	0.00	0.00	0.00	2.07
RP23-251B16.1	0.00	0.51	n.d.	0.001253054	0.00	0.00	1.75	0.66	0.20	0.00
CT030259.1	0.00	0.51	n.d.	0.001588722	0.82	1.21	0.07	0.36	0.78	0.66
AC158605.1	0.00	0.50	n.d.	0.001716383	0.00	0.00	0.00	0.00	0.00	0.00
Defb11	0.00	0.50	n.d.	0.001877362	0.00	0.00	0.00	0.00	0.00	0.00
Gm5481	0.00	0.45	n.d.	0.006607208	0.27	0.75	0.12	0.05	1.37	3.69
Dbil5	0.00	0.44	n.d.	0.00779344	0.40	0.86	0.34	0.38	0.44	0.14
Crygn	0.00	0.44	n.d.	0.008033479	0.00	0.00	0.22	0.14	0.18	0.21
Gm10028 (5 113489511..113490050)	0.00	0.42	n.d.	0.011216402	0.43	1.19	0.09	0.23	0.55	0.62

4930518I15Rik	0.00	0.36	n.d.	0.022216505	0.00	0.00	0.07	0.00	0.00	0.00
RP23-445E3.1	0.00	0.36	n.d.	0.022372399	0.00	0.00	0.00	0.00	0.00	0.00
AC159548.1	0.00	0.34	n.d.	0.028785355	0.00	0.24	0.00	0.00	0.20	0.00
Gm10028 (8 81393297..81393769)	0.00	0.34	n.d.	0.029440178	0.00	0.00	0.08	0.10	0.23	0.35
Rpl31-ps3	0.00	0.33	n.d.	0.033661502	0.00	0.00	0.00	0.00	0.00	0.00
RP23-279B13.1	0.00	0.32	n.d.	0.034655049	0.00	0.13	0.18	0.06	0.05	0.12
Gm5614	0.00	0.30	n.d.	0.042528836	0.12	0.57	0.04	0.11	1.04	2.74
Ubd	0.00	0.30	n.d.	0.042696987	0.00	0.00	0.00	0.00	0.06	0.07
RP23-103L13.1	0.00	0.30	n.d.	0.043700837	0.18	0.14	0.02	0.00	0.00	0.00
RP24-528G7.4	0.00	0.29	n.d.	0.045189999	0.47	0.28	0.00	0.00	0.11	0.00
E130304I02Rik	0.00	0.29	n.d.	0.04527213	0.00	0.10	0.09	0.03	0.17	0.00
Rpl26-ps2	0.00	0.29	n.d.	0.046008537	0.00	0.00	0.00	0.00	0.17	1.23
AC175246.1	0.00	0.29	n.d.	0.046008537	0.00	0.21	0.00	0.00	0.00	0.00
AC162033.2	0.00	0.29	n.d.	0.046496614	0.82	0.41	41.36	40.80	0.11	0.25
Gm7075	0.00	0.28	n.d.	0.049376411	0.46	0.13	0.04	0.00	0.00	0.00
AC102363.1	0.00	0.28	n.d.	0.049376411	0.00	0.00	1.44	0.00	0.00	0.00

B: Genes down-regulated by *T. gondii*

	Astrocytes				SkMCs		Neurons		Fibroblasts	
**Gene ID**	**n.i.**	***T. gondii***	**fold decrease**	***p* value**	**n.i.**	***T. gondii***	**n.i.**	***T. gondii***	**n.i.**	***T. gondii***
Gstp2	0.89	0.05	19.22	0.000403054	2.42	2.46	0.58	0.08	1.07	1.15
Rpl21-ps13	1.90	0.12	16.02	8.58674955e-08	0.58	0.26	0.00	0.06	0.07	0.00
RP23-16G8.1	0.48	0.04	12.18	0.006680168	0.14	0.28	0.00	0.01	0.27	0.15
Tcea1-ps1	0.36	0.04	10.25	0.039360499	10.79	14.81	0.07	0.01	0.49	0.65
RP23-312H15.4	0.67	0.07	10.25	0.002035862	0.00	0.00	0.00	0.00	0.00	0.00
AC097273.4	0.54	0.05	10.25	0.003351813	0.00	0.00	0.00	0.00	0.00	0.00
RP23-331L12.5	0.35	0.03	10.25	0.043077349	0.00	0.00	0.01	0.00	0.00	0.00
RP23-348N10.2	0.34	0.03	10.25	0.04824417	0.04	0.05	0.00	0.01	0.35	0.48
AC163446.1	3.04	0.32	9.61	9.633230499e-10	0.58	0.45	0.33	0.32	1.73	1.65
AC154435.1	1.05	0.11	9.61	0.000337352	0.20	0.24	0.15	0.15	1.52	0.64
RP23-471B6.3	0.41	0.05	8.97	0.027299884	0.05	0.00	0.12	0.05	0.26	0.18
Gm2058	0.35	0.04	8.97	0.046539112	0.00	0.06	0.30	0.21	0.05	0.00
Gm7263	1.45	0.16	8.97	1.601297694e-05	0.39	0.12	0.06	0.03	0.56	0.74
Rpl21-ps4	0.65	0.07	8.97	0.002626473	0.09	0.00	0.00	0.00	0.08	0.00
RP23-51G7.2	0.51	0.06	8.97	0.005099784	0.34	0.16	0.09	0.00	0.20	0.07
RP23-214E3.11	0.60	0.08	7.69	0.003838796	0.22	1.20	0.04	0.03	0.21	0.22
BX088539.2	1.40	0.18	7.69	3.776193686e-05	0.00	0.00	1.51	1.22	0.42	0.48
Gm10154	1.38	0.18	7.69	4.706087337e-05	1.42	2.31	0.04	0.06	10.76	16.62
AC157587.1	0.56	0.07	7.69	0.004347056	0.00	0.00	0.11	0.15	0.00	0.00
RP23-377K9.1	0.55	0.07	7.69	0.004419529	0.44	0.00	0.00	0.03	0.00	0.00
Rpl23a-ps1	0.52	0.07	7.69	0.004895347	0.00	0.29	0.45	0.29	0.39	0.53
Gm5278	3.31	0.46	7.21	6.72813059e-10	0.21	0.08	0.02	0.06	0.60	0.75
Smt3h2-ps2	1.15	0.17	6.73	0.000376911	0.36	0.18	0.15	0.18	0.20	0.11
Rpl31-ps1	0.54	0.08	6.41	0.006031167	0.41	0.24	0.07	0.27	0.39	0.55
Gm10003	0.51	0.08	6.41	0.007169002	0.29	0.34	0.00	0.00	0.00	0.00
RP23-183L1.2	0.44	0.07	6.41	0.023688413	0.04	0.10	0.35	0.45	0.00	0.04
CT027556.2	0.88	0.14	6.41	0.001700893	2.62	0.59	1.34	0.36	2.23	1.44
Myl6	2.59	0.42	6.19	1.093678871e-07	1.52	0.90	43.18	41.55	1.69	0.82
Rpl17-ps3	9.02	1.55	5.83	4.375477081e-16	17.57	6.81	1.05	0.73	11.51	3.27
Gm5519	0.45	0.08	5.77	0.022982738	0.05	0.39	0.00	0.03	0.45	0.10
Gm6807	0.41	0.07	5.77	0.037277672	0.48	0.36	0.06	0.03	0.87	0.93
Gm8290	2.30	0.43	5.31	8.741822579e-07	0.15	0.80	0.00	0.00	4.87	3.72
AC164155.8	1.90	0.37	5.13	4.30742771e-06	0.90	1.51	0.22	0.17	5.21	0.73
Gm9827	0.58	0.11	5.13	0.00775279	0.00	0.00	0.00	0.04	0.13	0.00
Gm10825	0.49	0.10	5.13	0.014146901	0.00	0.00	0.00	0.03	0.00	0.00
AL596181.1	0.44	0.09	5.13	0.032058947	0.62	0.37	0.10	0.06	0.69	0.22
RP23-344D2.12	0.44	0.09	5.13	0.032517742	0.00	0.00	0.20	0.12	0.00	0.00
Ugt1a10	5.00	1.02	4.90	4.721314771e-11	0.36	0.16	0.09	0.04	0.73	0.48
RP23-49E16.2	1.68	0.36	4.70	1.346081087e-05	1.59	5.62	0.00	0.00	0.27	0.16
Gm10134	0.75	0.17	4.49	0.006222972	0.10	0.00	0.00	0.03	0.00	0.00
AL645854.1	0.60	0.13	4.49	0.009676784	0.24	0.19	0.00	0.07	0.23	0.26
AC131919.1	0.84	0.20	4.27	0.005100783	0.56	0.94	0.44	1.55	0.68	0.77
RP23-255E18.1	0.48	0.11	4.27	0.022562952	0.00	0.00	0.01	0.01	0.00	0.00
1700041G16Rik	0.80	0.21	3.84	0.007833957	0.17	0.10	0.05	0.02	0.00	0.00
AC153591.2	0.56	0.15	3.84	0.015046413	0.18	0.42	0.12	0.10	0.34	0.00
1110059M19Rik	0.44	0.11	3.84	0.047575429	3.54	3.98	0.07	0.05	0.00	0.00
RP23-269L6.1	1.95	0.51	3.84	2.104659863e-05	0.49	0.50	0.46	0.34	0.99	0.28
Gm10925	0.85	0.22	3.84	0.006701842	0.54	0.32	0.00	0.00	0.51	0.00
RP24-406J12.2	0.84	0.22	3.84	0.006898952	0.40	1.09	0.13	0.12	2.39	4.41
Rpl21-ps7	0.91	0.24	3.84	0.005318925	0.14	0.17	0.02	0.13	0.14	0.31
Gm4883	0.46	0.12	3.84	0.036775923	0.00	0.26	0.00	0.02	0.07	0.16
AC160526.2 (8 26564338..26565629)	7.80	2.09	3.72	3.950702286e-12	6.44	5.10	5.94	4.61	16.79	32.40
AC125070.1	1.05	0.28	3.70	0.003563262	0.50	0.63	0.12	0.01	0.47	0.99
Atp5l-ps1	2.24	0.63	3.56	1.595692655e-05	1.27	0.90	0.28	0.44	0.81	2.37
AC118620.3	7.66	2.22	3.45	1.906217383e-11	7.20	4.25	0.72	0.34	4.14	3.35
AC102547.1	3.35	0.98	3.42	3.30291805e-07	0.80	0.78	0.00	0.12	0.13	0.00
AC102575.2	0.98	0.29	3.42	0.006199618	0.46	0.55	0.07	0.07	0.33	0.50
RP23-317F9.4	0.94	0.27	3.42	0.007149294	0.39	0.85	0.70	0.89	0.21	0.24
AL646051.1	0.48	0.14	3.42	0.037036948	0.06	0.07	0.02	0.03	0.00	0.00
Gm4799	1.11	0.34	3.30	0.004179027	0.59	0.76	0.19	0.19	0.61	0.88
RP23-169M24.1	2.04	0.62	3.30	4.973249252e-05	7.20	3.74	0.30	0.45	23.62	21.29
Mrpl48-ps	0.95	0.30	3.20	0.00842615	8.32	8.11	1.95	1.50	10.65	11.85
Gm8973	0.65	0.20	3.20	0.019855058	0.24	0.43	0.00	0.04	0.47	0.79
Camk2n2	0.63	0.20	3.20	0.020582875	0.60	0.04	7.51	11.01	0.48	0.48
AL670276.5	0.63	0.20	3.20	0.02069831	0.12	0.21	0.10	0.07	0.34	0.13
Dapl1	0.60	0.19	3.20	0.022085554	0.00	0.00	0.07	0.05	0.00	0.00
Olfr111	0.58	0.18	3.20	0.022980274	0.00	0.00	0.00	0.00	0.00	0.00
Rpl32-ps	0.50	0.16	3.20	0.03343633	0.19	0.45	0.09	0.00	0.81	0.10
Gprin2 (14 35003058..35003368)	1.33	0.41	3.20	0.001706105	0.13	0.30	0.98	1.02	0.00	0.00
2810474C18Rik	0.68	0.21	3.20	0.018335511	0.32	0.00	0.44	0.35	0.12	0.21
Gm10119	1.33	0.44	3.03	0.002238831	3.30	9.79	0.13	0.91	0.32	0.26
RP23-272P17.7	1.29	0.43	2.99	0.002859766	0.00	0.00	1.53	2.03	0.58	0.66
AC116136.2	0.73	0.24	2.99	0.019930974	0.69	0.82	0.00	0.03	0.47	0.42
Acbd7	0.53	0.18	2.99	0.030976016	0.07	0.25	0.23	0.35	0.07	0.00
Hist1h2ab	1.85	0.63	2.95	0.000181364	0.00	0.18	0.15	0.15	5.72	7.29
Ttbk1 (17 46579427..46579788)	2.05	0.71	2.88	0.00013851	0.11	0.00	22.94	20.74	0.00	0.00
E130208F15Rik	2.44	0.87	2.81	6.678376661e-05	1.83	1.77	1.21	1.38	1.81	2.10
Gm9294	1.23	0.44	2.80	0.005229347	0.78	1.38	0.03	0.03	1.39	0.31

AC124730.5	0.92	0.33	2.80	0.015311074	0.00	0.00	1.76	2.16	0.03	0.00
Dpm1	9.29	3.34	2.78	2.478276936e-10	4.75	4.11	6.15	6.87	3.20	3.65
RP23-321D1.4	6.58	2.42	2.72	6.201952283e-09	0.73	6.50	0.95	0.00	1.40	1.58
RP23-345D6.7	0.84	0.31	2.70	0.021404124	0.18	0.28	0.31	0.33	0.44	0.58
AC102115.2	9.77	3.66	2.67	3.681576278e-10	10.38	5.98	0.92	2.98	5.75	4.38
AC110508.2	20.89	7.97	2.62	1.724680786e-12	23.25	32.10	2.57	2.25	26.93	52.87
AC159210.2	0.55	0.21	2.56	0.046426052	0.58	0.61	0.00	0.02	0.06	0.07
Hist1h2bf	0.53	0.21	2.56	0.047899611	0.00	0.00	0.03	0.02	1.75	6.83
AC134581.2	3.17	1.24	2.56	2.062075919e-05	0.50	0.35	1.59	1.54	0.76	2.04
RP23-226O20.7	1.02	0.40	2.56	0.016363201	0.88	0.38	0.03	0.05	0.23	0.78
AC125099.7	0.63	0.25	2.56	0.040166042	0.00	0.23	0.48	0.12	0.94	0.21
AC099640.1	0.60	0.23	2.56	0.042497354	0.00	0.08	0.18	0.04	0.00	0.15
AC122037.2	1.57	0.62	2.52	0.001656586	0.32	0.30	0.25	0.35	0.17	0.17
AC159815.1	22.25	8.90	2.50	7.40052441e-12	32.63	25.94	2.81	0.93	12.46	16.48
Gm9774	1.01	0.41	2.46	0.020188937	1.16	0.83	0.19	0.19	0.51	0.40
AC165356.1	1.22	0.50	2.43	0.010909074	0.29	0.19	0.00	0.00	0.00	0.00
Gm10712	0.86	0.36	2.38	0.032409296	0.69	0.74	0.22	0.16	5.57	4.81
Gm6843	1.24	0.52	2.38	0.011360712	1.27	0.96	0.12	0.03	0.69	0.97
Rpl21-ps3	1.09	0.46	2.38	0.01811661	0.08	0.28	0.08	0.02	0.53	0.17
RP23-138L21.8	3.06	1.31	2.33	9.259879921e-05	0.36	2.39	0.00	0.00	1.51	0.70
Gm5239	49.03	21.15	2.32	8.492277298e-11	39.51	67.82	0.61	0.85	87.85	96.10
Gm10800	10.23	4.42	2.31	8.365852847e-09	1.58	1.31	40.14	4.78	6.94	4.27
CT025556.3	1.24	0.54	2.31	0.013216918	1.18	0.62	0.04	0.08	0.87	1.83
Rpl27a-ps1	0.74	0.32	2.31	0.047011843	0.70	0.37	0.05	0.05	2.80	2.50
Gstm3	0.88	0.39	2.26	0.038611402	0.00	0.00	0.02	0.02	0.08	0.00
RP23-330D1.5	2.76	1.23	2.24	0.000308536	2.49	2.25	0.00	0.01	1.26	7.76
RP23-356N23.1	1.43	0.64	2.24	0.007711731	0.00	0.00	0.06	0.00	0.00	0.21
Tmsb15b2	2.98	1.34	2.23	0.00019677	0.09	0.10	7.30	8.95	0.00	0.00
RP23-148P12.3	3.32	1.50	2.21	8.321948966e-05	1.66	1.96	0.16	0.31	2.60	4.10
Hist1h2ag	1.59	0.72	2.21	0.003882207	0.48	1.78	0.75	0.32	6.65	16.66
RP23-22M2.1	1.52	0.71	2.14	0.007070704	0.19	0.11	0.56	0.31	0.37	0.41
Pop7	1.50	0.70	2.14	0.007741514	1.21	2.78	1.10	0.81	1.05	0.96
Oaz3	1.34	0.63	2.14	0.014763955	0.38	0.15	2.41	1.79	0.16	0.05
Rnaset2a	2.66	1.25	2.12	0.000688628	9.02	17.26	0.17	0.27	1.87	2.20
Pgap1 (1 54532541..54532743)	1.64	0.80	2.05	0.005439809	1.17	0.92	0.00	0.00	0.37	0.00
AC153821.1	2.53	1.25	2.03	0.001461733	1.65	0.82	0.76	0.63	1.35	1.13
AC135640.1	1.07	0.53	2.02	0.041950355	0.13	0.30	0.00	0.00	0.00	0.00
RP23-27I6.8	4.27	2.12	2.01	7.104237838e-05	1.75	1.41	0.71	0.90	0.79	1.11
AL645845.1	2.03	0.00	n.d.	5.911859751e-09	1.29	0.00	0.42	0.75	1.22	0.00
AC107663.1	1.69	0.00	n.d.	2.961035197e-08	0.80	0.00	0.00	0.47	0.76	0.86
BX682545.2	0.75	0.00	n.d.	0.000417382	0.00	0.00	0.00	0.00	0.00	0.00
AC116763.1	0.71	0.00	n.d.	0.00051134	0.34	0.00	0.00	0.00	1.29	0.36
AL732587.1	0.62	0.00	n.d.	0.000811807	0.00	0.00	0.00	0.00	0.00	0.00
Gm10328	0.57	0.00	n.d.	0.000982688	0.27	0.00	0.18	0.08	0.26	0.88
0610010O12Rik	0.51	0.00	n.d.	0.001569328	0.12	0.43	0.00	0.10	0.11	0.00
AC084390.1	0.50	0.00	n.d.	0.001795741	0.12	0.00	0.08	0.07	0.23	0.38
Hist1h3i	0.49	0.00	n.d.	0.002195846	0.09	0.44	0.00	0.19	2.14	1.92
AC121264.1	0.48	0.00	n.d.	0.003533663	0.00	0.00	0.00	0.00	0.21	0.24
AC131084.1	0.44	0.00	n.d.	0.00774123	0.00	0.00	0.00	0.00	0.00	0.00
AL731674.1	0.44	0.00	n.d.	0.00774123	0.00	0.00	0.27	0.00	0.00	0.00
RP23-263F7.1	0.44	0.00	n.d.	0.007905317	0.36	0.55	0.05	0.07	0.49	1.11
Gm10199	0.43	0.00	n.d.	0.009089733	0.07	0.16	0.00	0.00	0.13	0.07
AC135017.1	0.40	0.00	n.d.	0.013545285	28.01	16.52	0.37	0.63	0.00	0.00
Gm5483	0.39	0.00	n.d.	0.015537782	0.00	0.00	0.00	0.00	0.00	0.00
AC154237.2	0.37	0.00	n.d.	0.02178823	0.00	0.00	0.00	0.00	0.00	0.00
AC161876.1	0.37	0.00	n.d.	0.02178823	0.00	1.23	0.00	0.10	0.66	0.37
Gm5244	0.36	0.00	n.d.	0.024347023	0.00	0.00	0.00	0.00	0.00	0.00
RP23-27B23.4	0.34	0.00	n.d.	0.029302018	0.06	0.30	0.00	0.06	0.00	0.07
AC125091.1	0.32	0.00	n.d.	0.034112297	0.15	0.09	0.00	0.00	0.22	0.17
AC159614.2	0.32	0.00	n.d.	0.034529372	0.00	0.18	0.00	0.00	0.15	0.16
RP24-535O17.2	0.32	0.00	n.d.	0.035636912	0.40	0.24	0.07	0.03	0.10	0.00
Ghrl	0.31	0.00	n.d.	0.039252067	0.44	0.60	0.92	0.81	0.07	0.16
RP23-309E16.7	0.31	0.00	n.d.	0.039454025	0.00	0.00	0.09	0.04	0.00	0.00
RP23-71K8.5	0.30	0.00	n.d.	0.040659417	0.00	0.00	0.28	0.54	0.14	0.16

[a]Data represent RPKM values if not specified otherwise
[b]not determined

Supplementary Table S5: Expression of host cell genes regulated by *T. gondii* in fibroblasts (at least 2-fold; p < 0.05)

A: Genes up-regulated by *T. gondii*

	Fibroblasts				SkMCs		Neurons		Astrocytes	
**Gene ID**	**n.i.**	***T. gondii***	**fold increase**	***p* value**	**n.i.**	***T. gondii***	**n.i.**	***T. gondii***	**n.i.**	***T. gondii***
2510046G10Rik	0.11	0.95	9.05	0.000660323	0.00	0.39	0.14	0.58	0.23	0.55
Fxyd3	0.07	0.66	9.05	0.002458326	0.00	0.09	0.07	0.00	0.16	0.13
Gm5292	0.06	0.57	9.05	0.003414574	0.07	0.08	0.00	0.08	0.21	0.00
Hist1h2bk	0.36	2.98	8.37	2.640951446e-09	0.38	0.71	0.00	0.00	0.24	0.06
RP23-100C7.4	0.05	0.41	7.92	0.028559772	0.05	0.00	0.02	0.03	0.06	0.27
Rpl26-ps2	0.17	1.23	7.35	0.000176038	0.00	0.00	0.00	0.00	0.00	0.29
AC153915.2	1.16	8.55	7.35	6.020363878e-17	2.45	2.17	12.29	18.11	0.00	0.00
Hist2h3b	1.96	13.41	6.84	7.301679931e-20	0.56	0.22	0.15	0.08	0.20	0.00
Gm5048	0.13	0.86	6.79	0.00169501	0.07	0.39	0.09	0.04	0.14	0.22
AC172027.2	0.08	0.53	6.79	0.005647397	0.08	0.10	0.00	0.00	0.00	0.20
AC034255.1	0.07	0.45	6.79	0.020669232	0.00	0.00	0.00	0.02	0.07	0.06
Aqp2	0.10	0.67	6.50	0.003790258	0.03	0.03	0.01	0.00	0.03	0.02
Ttpal	0.51	3.33	6.50	1.238896261e-09	0.38	1.06	1.11	1.24	0.21	0.33
1700069B07Rik	0.09	0.55	6.22	0.006023287	0.47	0.28	0.18	0.15	0.05	0.08
RP23-330D1.5	1.26	7.76	6.15	2.099026446e-15	2.49	2.25	0.00	0.01	2.76	1.23
AC102103.1	0.13	0.77	6.03	0.003042351	0.22	0.37	0.45	0.53	0.33	0.48
AC154377.2	0.10	0.57	5.65	0.006716078	0.43	0.25	3.85	3.67	1.34	1.49
RP23-419P16.11	0.08	0.46	5.65	0.019463838	0.26	0.20	0.14	0.05	0.18	0.43
Fmod	0.23	1.28	5.49	0.000276097	1.03	1.36	0.12	0.14	96.10	96.12
Gm5921	0.29	1.46	5.01	9.539058425e-05	3.76	7.89	0.08	0.11	0.41	0.11
RP23-480B19.17	0.27	1.32	4.95	0.000293042	0.00	0.04	0.01	0.02	0.00	0.00
3110079O15Rik	0.23	1.13	4.90	0.00098186	0.00	0.00	0.03	0.00	0.00	0.00
RP24-66I20.1	0.15	0.73	4.81	0.005615134	0.24	0.19	0.05	0.02	0.93	1.12
Gm5619	0.32	1.50	4.69	8.55534458e-05	0.34	0.40	0.00	0.01	0.10	0.00
1810011O10Rik	0.11	0.52	4.52	0.011907647	0.48	0.43	0.08	0.24	0.00	0.10
Gm10247	*0.11*	*0.50*	*4.52*	*0.016214313*	*0.00*	*0.00*	*0.00*	*0.00*	*0.00*	*0.19*
AC111140.2	0.10	0.45	4.52	0.034515701	0.00	0.00	0.00	0.00	0.00	0.00
Hist1h2ah	0.99	4.50	4.52	4.339771854e-10	0.19	0.22	0.00	0.06	0.70	0.63
Rps24-ps2	0.18	0.83	4.52	0.004578937	2.13	0.00	0.16	0.08	0.92	0.48
Hist1h2ae	2.55	11.44	4.49	3.094825497e-16	0.34	0.10	0.16	0.12	0.71	0.69
RP23-29H5.3	0.15	0.64	4.24	0.009764551	1.39	1.83	0.31	0.54	0.42	0.16
AC171328.1	0.26	1.02	3.96	0.003248437	0.14	0.16	0.09	0.00	0.00	0.11
Mia1	0.19	0.77	3.96	0.008032388	0.41	0.72	1.71	2.43	15.91	15.30
Gm6378	0.17	0.65	3.96	0.011155537	0.17	0.00	0.00	0.05	0.00	0.00
Hist1h2bf	1.75	6.83	3.91	6.03655521e-12	0.00	0.00	0.03	0.02	0.53	0.21
Gm7589	5.93	22.83	3.85	2.948091116e-17	11.64	11.19	1.05	6.69	7.57	4.73
RP23-74L9.6	0.19	0.72	3.85	0.009878943	0.79	1.45	0.26	0.23	0.92	0.62
AC113270.1	1.28	4.90	3.82	1.310948486e-09	1.35	3.72	0.02	0.00	0.54	0.95
Rpl13-ps3	2.00	7.57	3.79	3.875275395e-12	0.68	1.90	0.10	0.09	1.24	1.17
Cd74	0.21	0.76	3.68	0.00993222	0.25	1.13	0.29	0.93	1.70	1.89
3830403N18Rik	0.14	0.53	3.68	0.018310837	27.97	25.65	0.00	0.00	0.56	0.37
Ppapdc2	0.15	0.55	3.58	0.01859464	0.15	0.47	0.04	0.03	0.07	0.02
Hist1h2ac	*3.39*	*11.58*	*3.42*	*9.685193275e-14*	*0.97*	*2.10*	*0.16*	*0.05*	*4.27*	*3.47*
Gm10328	0.26	0.88	3.39	0.008941166	0.27	0.00	0.18	0.08	0.57	0.00
B930041F14Rik	0.22	0.75	3.39	0.012748907	0.29	0.21	0.09	0.04	0.32	0.21
Gm10034	0.21	0.70	3.39	0.014599289	0.56	0.29	0.84	1.01	1.94	1.49
AC113069.6	0.16	0.55	3.39	0.020943236	0.77	0.76	0.53	0.32	1.13	1.77
AC144852.2	0.64	2.16	3.39	2.879101849e-05	4.14	3.43	9.63	9.40	1.53	1.84
RP24-179M18.3	0.41	1.41	3.39	0.000815894	1.05	1.03	0.31	0.20	0.55	0.79
RP23-226O20.7	0.23	0.78	3.39	0.011997501	0.88	0.38	0.03	0.05	1.02	0.40
Hist1h4a	4.49	15.06	3.36	2.724622925e-14	0.00	0.51	0.23	0.08	1.36	0.77
Gm6635	0.53	1.69	3.20	0.000137306	0.09	0.00	0.03	0.00	0.00	0.00
Rnf151	0.19	0.58	3.11	0.02502276	0.20	0.29	0.00	0.00	0.00	0.16
Hist1h2bg	2.11	6.44	3.06	9.452230246e-10	0.84	0.78	0.10	0.09	3.29	3.06
Gm6139	0.54	1.64	3.04	0.000266306	3.76	0.77	0.01	0.01	0.14	0.00
9430002A10Rik	0.23	0.71	3.02	0.020527172	0.00	0.00	0.35	0.14	0.61	0.41
Ost4	0.96	2.91	3.02	8.131415262e-06	2.03	1.20	0.11	0.59	1.07	1.95
Mmp9	0.60	1.79	2.98	0.000186955	0.03	0.27	0.25	0.31	8.44	8.66
AC147612.2	1.39	4.13	2.98	1.943470088e-07	0.44	1.15	0.05	0.01	0.98	0.64
Hist1h2aa	0.75	2.23	2.97	7.060070487e-05	0.40	0.23	0.03	0.00	0.10	0.00
Hspb2	0.47	1.39	2.94	0.00186081	82.53	77.29	0.02	0.01	2.05	1.40
Fgf21	0.39	1.13	2.94	0.00620203	9.04	7.89	0.01	0.00	0.04	0.00
Atp5l-ps1	0.81	2.37	2.94	5.313412191e-05	1.27	0.90	0.28	0.44	2.24	0.63
Zfp580	0.66	1.94	2.92	0.000163321	0.85	1.56	36.30	29.02	1.74	1.75
Frat2	0.84	2.44	2.90	5.047717823e-05	1.00	0.98	1.27	1.72	2.16	2.92
AC121903.1	0.31	0.88	2.83	0.016474607	2.07	1.80	0.07	0.02	0.06	0.00
Ndnl2	0.25	0.71	2.81	0.025259532	0.07	0.08	0.00	0.00	0.07	0.05
Hist1h4k	3.02	8.42	2.78	6.343457984e-10	0.12	0.29	0.04	0.04	1.16	0.91
AC127252.2	2.15	5.97	2.78	1.792469146e-08	8.64	9.59	0.67	0.39	8.67	9.48
Gm16382	1.07	2.96	2.78	1.647050791e-05	5.82	4.82	0.59	0.21	3.44	2.01
Timm23	0.29	0.81	2.76	0.02106745	0.76	0.57	0.10	0.08	0.40	0.31
Cst6	0.56	1.56	2.76	0.00096995	19.62	22.44	0.47	0.71	1.88	1.90
Hist1h4h	5.61	15.42	2.75	4.635318965e-12	0.33	0.77	0.93	0.76	14.79	22.68
Gm5481	1.37	3.69	2.69	1.66190806e-06	0.27	0.75	0.12	0.05	0.00	0.45
AC134581.2	0.76	2.04	2.69	0.00025337	0.50	0.35	1.59	1.54	3.17	1.24
0610038B21Rik	0.38	1.03	2.69	0.012791329	0.51	0.63	0.08	0.21	0.19	0.23
Gm6030	*0.23*	*0.62*	*2.64*	*0.037391707*	*0.25*	*0.10*	*1.89*	*1.19*	*0.69*	*0.54*
1700019G06Rik	0.33	0.87	2.64	0.021563042	0.23	0.27	0.11	0.17	0.73	0.38
Hist2h4 (13 21903612..21904018)	0.27	0.72	2.64	0.0304111	0.48	0.23	0.09	0.06	0.40	0.16
Gm5614	1.04	2.74	2.64	5.594248087e-05	0.12	0.57	0.04	0.11	0.00	0.30
Hist1h3e	0.96	2.53	2.62	0.000104065	0.05	0.05	0.09	0.09	0.34	0.23
Hist1h3h	3.45	9.00	2.61	1.527130218e-09	0.00	0.28	0.28	0.18	0.08	0.13
Ng23	0.53	1.34	2.54	0.005378088	0.14	0.00	0.22	0.03	0.19	0.27
CR974451.2	0.40	1.02	2.54	0.016769764	0.32	0.25	0.03	0.06	0.33	0.61
RP23-325M14.7	0.24	0.60	2.54	0.043214299	0.06	0.00	0.04	0.13	0.66	0.31
Hist1h2bb	2.03	5.12	2.52	4.953750879e-07	0.08	0.10	0.00	0.00	0.26	0.34
Hist1h2ag	6.65	16.66	2.51	4.029307446e-11	0.48	1.78	0.75	0.32	1.59	0.72
Hist1h1b	1.58	3.90	2.47	4.120485306e-06	0.00	0.06	0.00	0.02	0.93	0.47
Il17d	0.35	0.85	2.45	0.030155995	0.15	0.18	10.62	11.03	3.13	2.79
Hist1h3g	1.49	3.54	2.37	1.546036989e-05	0.16	0.09	0.28	0.18	0.50	0.58
Hist2h2aa2	2.83	6.70	2.37	1.005989647e-07	1.21	2.34	2.75	2.16	1.94	3.27
Gm9790	0.85	2.01	2.36	0.000822699	1.30	0.38	0.55	0.21	1.20	1.67
Hist1h2bj	3.46	8.16	2.36	3.292109484e-08	1.37	0.19	0.13	0.14	2.30	1.60
Hist1h2bl	1.08	2.55	2.36	0.000289277	0.76	0.56	0.00	0.00	0.20	0.23
Hist1h4c	4.82	11.33	2.35	1.7486981e-09	0.29	0.35	0.03	0.23	0.72	0.56

Hist1h4m	6.22	14.55	2.34	6.510479547e-10	0.72	0.00	0.12	0.08	2.36	3.62
Hist2h4 (3 96066913..96067232)	3.60	8.29	2.30	5.632518864e-08	0.49	0.14	1.19	0.67	0.26	0.30
Hist2h2ac	4.02	9.16	2.28	3.168503991e-08	0.52	1.75	0.07	0.54	0.70	0.43
AC154425.1	0.52	1.18	2.26	0.018263903	13.68	19.69	0.00	0.24	2.31	1.80
Rnf183	0.70	1.58	2.26	0.003609378	0.00	0.00	0.02	0.04	0.08	0.04
AC101743.1	0.39	0.88	2.26	0.038515354	0.71	0.48	0.30	0.27	0.97	0.50
Gm10495	0.33	0.76	2.26	0.048661198	1.41	1.45	0.23	0.00	0.56	0.29
RP23-263F7.1	0.49	1.11	2.26	0.021989866	0.36	0.55	0.05	0.07	0.44	0.00
Hist1h4j	1.32	2.94	2.22	0.000225783	0.00	0.06	0.02	0.10	0.52	0.86
Hist1h4d	10.74	23.01	2.14	1.134219155e-09	0.00	0.32	0.38	0.29	1.25	1.35
AC127338.1	0.66	1.41	2.14	0.011596317	1.62	1.63	0.25	0.22	1.22	0.95
Gp1bb	0.97	2.06	2.13	0.001962925	1.85	1.76	5.07	4.94	0.76	0.64
Rps2 ps6	*0.73*	*1.55*	*2.13*	*0.006597866*	*0.81*	*2.07*	*0.03*	*0.01*	*0.05*	*0.04*
Hist4h4	3.55	7.55	2.13	5.815446824e-07	0.22	0.00	0.04	0.00	0.00	0.09
Hist1h2ao	6.83	14.52	2.13	1.11275933e-08	1.99	2.25	1.60	1.33	2.74	2.71
CT025556.3	0.87	1.83	2.10	0.003064685	1.18	0.62	0.04	0.08	1.24	0.54
S100a5	0.59	1.25	2.10	0.021820231	0.09	0.00	0.00	0.00	0.00	0.15
Gm9798	3.41	7.13	2.09	1.146848134e-06	5.20	5.50	0.89	0.90	3.53	2.95
AC125070.1	0.47	0.99	2.09	0.042941444	0.50	0.63	0.12	0.01	1.05	0.28
RP23-428I4.3	0.47	0.98	2.07	0.045077727	0.33	0.00	0.08	0.00	0.26	0.14
AC160526.1	11.21	23.17	2.07	3.754153899e-09	10.30	13.40	1.14	0.74	8.35	4.64
Hist1h1d	0.56	1.15	2.06	0.031693418	0.25	0.21	0.00	0.06	0.75	0.64
Hist1h2bm	0.99	2.03	2.06	0.00282636	0.47	0.11	0.00	0.03	0.30	0.23
Hist1h4i	2.87	5.87	2.04	7.97525376e-06	2.18	2.00	0.58	0.85	13.81	13.31
AC165946.1	0.50	1.02	2.04	0.045005205	2.42	4.35	7.30	5.62	0.33	0.52
AC125535.1	1.27	2.59	2.04	0.001266065	2.68	2.53	1.30	2.02	1.98	3.75
AC164158.1	1.30	2.62	2.02	0.001310045	4.29	1.14	0.89	1.52	2.35	2.32
Gemin4	1.03	2.07	2.01	0.003197896	0.30	0.24	0.33	0.34	0.45	0.48
Gm8618	1.97	3.95	2.01	9.751507196e-05	0.92	1.54	0.20	0.24	0.65	0.63
Hist1h3f	0.52	1.03	2.00	0.047501007	0.04	0.00	0.03	0.02	0.13	0.14
RP23-185A18.17	0.00	0.54	n.d.	0.001111287	0.10	0.24	0.03	0.00	0.21	0.08
Gm10324	0.00	0.53	n.d.	0.001170636	0.08	0.00	0.00	0.00	0.00	0.00
Hist2h2ab	0.00	0.53	n.d.	0.001190314	0.10	0.00	0.00	0.03	0.10	0.24
Gm10507	0.00	0.45	n.d.	0.005676154	0.00	0.33	0.00	0.00	0.15	0.00
2310034G01Rik	0.00	0.38	n.d.	0.018811787	0.00	0.00	0.00	0.00	0.09	0.07
RP23-239F1.2	0.00	0.37	n.d.	0.020877996	0.04	0.05	0.02	0.04	0.04	0.16
Gm10431	0.00	0.32	n.d.	0.03585272	0.00	0.00	0.00	0.00	0.00	0.00
AC139320.1	0.00	0.32	n.d.	0.03585272	0.05	0.18	0.00	0.75	0.00	0.20
Gm10703	0.00	0.31	n.d.	0.037467961	0.00	0.00	0.09	0.00	0.92	2.28
1700012B09Rik	0.00	0.31	n.d.	0.040488759	1.93	1.54	0.06	0.63	1.20	1.87
RP23-459L15.1	0.00	0.30	n.d.	0.041428613	0.00	0.00	0.00	0.00	0.00	0.00
Gm10635	0.00	0.30	n.d.	0.042206411	0.00	0.00	0.00	0.00	0.00	0.00
2310001K24Rik	0.00	0.28	n.d.	0.048485785	0.35	0.93	0.06	0.25	0.00	0.00
AC184160.2	0.00	2.07	n.d.	4.853014716e-09	0.00	0.00	0.00	0.00	0.00	0.53

B: Genes down-regulated by T. gondii

	Fibroblasts				SkMCs		Neurons		Astrocytes	
**Gene ID**	**n.i.**	***T. gondii***	**fold decrease**	***p* value**	**n.i.**	***T. gondii***	**n.i.**	***T. gondii***	**n.i.**	***T. gondii***
Zfa	0.56	0.06	10.02	0.003162278	0.14	0.27	0.02	0.05	0.09	0.06
Wfikkn1	0.55	0.06	8.84	0.003779008	0.12	0.00	0.04	0.03	0.18	0.19
Rpl32-ps	0.81	0.10	7.96	0.001612515	0.19	0.45	0.09	0.00	0.50	0.16
AC164155.8	5.21	0.73	7.17	5.617221974e-13	0.90	1.51	0.22	0.17	1.90	0.37
RP23-103L13.9	0.46	0.08	6.19	0.017291366	0.42	0.08	0.00	0.00	0.96	1.49
AC183095.1	1.35	0.22	6.19	0.000111348	18.61	14.93	6.92	7.91	6.49	5.01
RP23-149D5.5	0.60	0.10	6.19	0.0052697	0.36	0.53	0.06	0.00	0.38	0.30
AC083948.1	0.58	0.11	5.31	0.007397547	0.71	0.48	0.07	0.06	0.11	0.25
RP23-386N10.4	0.67	0.13	5.31	0.005584387	0.00	0.00	0.00	0.00	0.00	0.00
RP23-14F5.5	2.54	0.54	4.73	6.973245356e-07	3.06	1.22	1.02	1.26	0.90	1.04
Gm10709	1.21	0.26	4.64	0.000758083	0.06	0.07	0.16	0.02	0.06	0.05
AC125099.7	0.94	0.21	4.42	0.003153725	0.00	0.23	0.48	0.12	0.63	0.25
RP23-135L5.5	0.85	0.19	4.42	0.00446387	1.35	1.06	0.32	0.29	1.14	1.41
Gm5519	0.45	0.10	4.42	0.033109945	0.05	0.39	0.00	0.03	0.45	0.08
Gm10120	*0.45*	*0.10*	*4.42*	*0.033339572*	*0.67*	*0.90*	*0.03*	*0.03*	*0.40*	*0.63*
Gm9294	1.39	0.31	4.42	0.000275351	0.78	1.38	0.03	0.03	1.23	0.44
1110020C17Rik	0.49	0.11	4.42	0.019713942	0.00	0.00	0.00	0.00	0.11	0.00
RP23-195K8.8	0.64	0.15	4.20	0.00995039	0.25	0.25	0.00	0.01	0.30	0.47
Gm9847	0.64	0.16	4.13	0.010389405	0.19	0.11	0.11	0.07	0.05	0.16
AL611931.1	0.52	0.13	4.13	0.014265542	0.31	0.28	0.03	0.16	0.37	0.35
Gm8394	1.94	0.47	4.10	1.437844619e-05	0.44	0.94	0.01	0.01	0.17	0.69
Spa17	1.51	0.37	4.07	0.000152184	0.62	0.82	7.21	7.38	15.08	15.52
RP24-114E18.3	1.12	0.28	3.98	0.002008118	0.52	2.79	0.08	0.04	0.55	0.32
Gm9777	0.89	0.22	3.98	0.005169309	0.31	0.37	0.00	0.00	0.33	0.17
RP23-129H12.1	4.61	1.17	3.94	1.743227623e-09	2.68	2.22	4.11	3.11	12.96	13.21
RP24-93B5.3	1.02	0.27	3.76	0.003824236	0.00	0.07	0.02	0.02	0.07	0.00
Gm14139	0.45	0.12	3.71	0.047496229	0.49	0.45	0.00	0.01	0.05	0.02
AC116763.1	1.29	0.36	3.54	0.001372313	0.34	0.00	0.00	0.00	0.71	0.00
AC124479.2	1.12	0.32	3.54	0.003033745	0.81	0.35	0.00	0.00	0.08	0.12
RP23-269L6.1	0.99	0.28	3.54	0.005335898	0.49	0.50	0.46	0.34	1.95	0.51
Serinc4	2.04	0.58	3.54	2.970271637e-05	0.34	0.83	1.66	3.04	4.13	2.27
Rpl17-ps3	11.51	3.27	3.52	5.137866473e-14	17.57	6.81	1.05	0.73	9.02	1.55
Gm5045	2.09	0.60	3.46	2.959186167e-05	0.33	0.39	0.08	0.14	0.49	0.42
RP23-233B9.5	1.51	0.44	3.43	0.000369705	1.34	1.39	3.06	2.71	1.03	0.59
Gm5528	0.83	0.26	3.24	0.011705081	0.00	0.38	0.44	0.05	0.00	0.85
RP23-27I6.2	0.84	0.27	3.09	0.013573232	0.17	0.25	1.84	1.91	0.73	0.69
AC132380.1	0.77	0.25	3.09	0.016185756	0.46	0.55	2.40	3.29	0.85	1.05
AL596181.1	0.69	0.22	3.09	0.019790405	0.62	0.37	0.10	0.06	0.44	0.09
Ccdc106	0.49	0.16	3.09	0.038580927	0.30	0.21	9.04	9.33	2.63	2.52
AC187103.2	0.74	0.24	3.09	0.017407123	0.45	0.36	0.25	0.42	0.74	0.62
Rpl21-ps3	0.53	0.17	3.09	0.028026318	0.08	0.28	0.08	0.02	1.09	0.46
Rpl7a-ps3	1.10	0.36	3.03	0.006236303	0.00	0.17	0.00	0.07	0.10	0.04
Ogn	1.13	0.38	2.96	0.006043617	44.47	41.56	0.01	0.00	14.90	13.00
RP23-360A2.3	0.83	0.28	2.95	0.016336599	0.12	0.07	0.00	0.05	0.15	0.02
Gm10193	2.69	0.91	2.95	2.094786842e-05	0.74	1.67	0.07	0.11	1.31	0.98
RP24-132L16.6	0.78	0.27	2.87	0.020134572	0.06	0.07	0.04	0.04	0.53	0.57
AC167249.3	89.71	31.29	2.87	1.058207486e-13	58.36	21.83	33.13	16.31	70.50	62.02
Grpr	0.61	0.21	2.86	0.029898538	0.00	0.00	0.35	0.23	0.02	0.09
Trmt112-ps	4.65	1.64	2.83	1.703912836e-07	4.18	3.01	0.30	0.30	3.23	3.61
AC138025.1	15.13	5.50	2.75	5.036834271e-12	4.49	6.09	0.09	0.17	7.74	4.33

AC159281.2	1.25	0.47	2.65	0.006227462	0.08	0.19	0.00	0.02	0.21	0.46
Iqcf5	0.99	0.37	2.65	0.015336949	0.00	0.10	0.76	0.83	0.09	0.00
Ly6c2	0.56	0.21	2.65	0.04135901	63.70	56.99	0.00	0.00	0.00	0.00
AC122248.2 (7 128090547..128090944)	1.40	0.53	2.65	0.003230019	0.20	0.58	3.59	2.85	0.72	1.78
Wdr5b	1.10	0.41	2.65	0.010901072	2.25	1.88	3.51	2.73	2.59	2.04
AL627237.1	1.09	0.41	2.65	0.011175794	0.66	0.13	0.09	0.03	0.35	0.59
CT010583.2	1.09	0.41	2.65	0.011346846	0.29	0.11	0.53	0.28	0.00	0.16
AL807745.1	0.76	0.29	2.65	0.027287906	0.00	0.32	0.00	0.00	0.00	0.22
AL929249.1	0.66	0.25	2.65	0.034047533	0.38	0.54	0.00	0.00	0.32	0.13
RP23-369H9.2	0.65	0.24	2.65	0.034708244	0.46	0.40	0.11	0.05	0.18	0.00
AC087891.2	0.58	0.22	2.65	0.039421907	0.20	0.00	0.00	0.00	0.65	0.84
A830005F24Rik	0.56	0.21	2.65	0.041045714	0.59	0.41	0.56	0.24	0.72	0.77
T2	0.51	0.19	2.65	0.04647141	0.00	0.00	0.16	0.10	0.04	0.09
Gm5077	0.69	0.27	2.56	0.035377824	0.14	0.06	0.04	0.01	0.03	0.00
Ctla2a	0.74	0.30	2.48	0.03620336	18.59	15.01	0.07	0.03	18.12	17.17
Gjb5	0.77	0.31	2.45	0.035848524	0.40	0.29	0.00	0.03	0.09	0.20
Gm6472	18.53	7.65	2.42	5.364332866e-11	18.59	20.15	0.11	0.29	0.42	0.43
AC154435.1	1.52	0.64	2.36	0.003659473	0.20	0.24	0.15	0.15	1.05	0.11
RP23-353E13.2	1.03	0.44	2.36	0.022757893	0.23	0.11	1.02	1.27	0.71	0.59
Aif1	0.69	0.29	2.36	0.047316082	0.05	0.00	0.78	0.49	64.37	64.68
Gm9938	1.75	0.75	2.32	0.001466954	1.25	1.24	1.34	1.75	3.43	3.41
AC126045.1	1.46	0.65	2.26	0.006377106	0.60	0.87	0.63	0.39	0.00	0.06
Hist1h2ad	3.27	1.45	2.25	7.706576057e-05	0.88	0.64	0.11	0.18	0.64	1.94
Palm2	2.46	1.10	2.24	0.000581824	14.55	13.87	12.94	12.75	7.05	8.70
Efcab2	1.04	0.47	2.21	0.029246043	1.10	1.17	1.26	1.03	6.13	6.59
RP23-56A7.3	7.49	3.43	2.19	3.307566615e-07	3.81	3.35	0.11	0.10	0.93	2.41
RP23-159D11.3	1.41	0.65	2.18	0.010314137	0.28	0.44	0.03	0.01	0.05	0.00
RP23-138L21.8	1.51	0.70	2.16	0.00680709	0.36	2.39	0.00	0.00	3.06	1.31
Ubp1	5.78	2.72	2.12	4.210903606e-06	6.45	6.46	18.70	20.05	8.88	7.67
Rcsp18	0.93	0.44	2.10	0.04691919	0.00	0.00	1.35	2.08	0.00	0.00
Zfp97	2.32	1.11	2.08	0.001628441	2.66	2.99	0.61	0.82	3.59	3.41
Gm8355	2.11	1.02	2.06	0.002408172	2.07	2.95	0.41	0.21	2.26	2.12
RP23-29H22.3	1.95	0.94	2.06	0.003056432	1.58	1.45	0.23	0.54	0.80	0.96
Myl6	1.69	0.82	2.06	0.004318806	1.52	0.90	43.18	41.55	2.59	0.42
D630037F22Rik (10 55767972..55881999)	1.61	0.78	2.06	0.00607596	1.79	2.17	0.60	0.66	2.18	2.63
Gm10661	1.08	0.52	2.06	0.036766436	0.16	0.38	0.26	0.52	0.34	0.40
RP23-382C18.5	5.91	2.90	2.04	7.785371353e-06	0.62	2.45	0.00	0.02	0.15	0.23
Gm5595	1.42	0.70	2.04	0.015013779	0.56	0.77	0.41	0.48	0.83	1.18
RP23-328F3.3	1.05	0.52	2.03	0.042365215	0.24	0.28	2.19	2.01	0.51	0.43
Lrrc17	3.02	1.50	2.01	0.000644258	20.69	21.20	0.00	0.01	0.13	0.06
RP23-82I5.15	0.65	0.00	n.d.	0.000688934	0.07	0.08	0.09	0.12	1.01	0.85
Gm10135	0.59	0.00	n.d.	0.000917707	0.15	0.00	0.15	0.09	0.00	0.00
Gm10925	0.51	0.00	n.d.	0.001475454	0.54	0.32	0.00	0.00	0.85	0.22
RP23-162P18.4	0.45	0.00	n.d.	0.00583035	0.12	0.14	0.27	0.07	0.00	0.10
AC161053.2	0.44	0.00	n.d.	0.007898737	0.92	1.63	2.82	2.00	0.48	1.13
RP23-97P13.1	0.42	0.00	n.d.	0.0095673	0.00	0.00	0.00	0.03	0.09	0.15
RP23-349N21.2	0.41	0.00	n.d.	0.012123468	0.00	0.00	0.00	0.00	0.00	0.00
Rpl21-ps14	0.38	0.00	n.d.	0.018604911	0.24	0.00	0.00	0.02	0.17	0.00
Pgap1 (1 54532541..54532743)	0.37	0.00	n.d.	0.02084646	1.17	0.92	0.00	0.00	1.64	0.80
RP23-455B19.2	0.36	0.00	n.d.	0.02431284	0.00	0.00	1.74	1.92	0.00	0.10
AC153591.2	0.34	0.00	n.d.	0.029370112	0.18	0.42	0.12	0.10	0.56	0.15
AC149085.6	0.33	0.00	n.d.	0.033744537	0.11	0.13	0.00	0.07	0.00	0.09
AC104882.1	0.32	0.00	n.d.	0.034053844	0.00	0.13	1.62	1.58	0.36	0.56
Gm9900	0.31	0.00	n.d.	0.038628161	0.00	0.00	0.14	0.13	0.34	0.18
AC162922.1	0.31	0.00	n.d.	0.039526675	0.00	0.00	0.00	0.03	0.23	0.18
Gm9808	0.31	0.00	n.d.	0.039750384	0.08	0.10	0.05	0.16	0.09	0.00
Hnrnpa1l2	0.30	0.00	n.d.	0.041084647	0.08	0.00	0.18	0.09	0.04	0.00
4930528A17Rik	0.30	0.00	n.d.	0.043710553	0.00	0.00	0.00	0.00	0.16	0.13
AL645845.1	1.22	0.00	n.d.	1.53280569e-05	1.29	0.00	0.42	0.75	2.03	0.00
AC166332.1	1.18	0.00	n.d.	2.200955391e-05	0.00	0.00	1.75	1.32	1.31	1.71
AC122193.1	1.53	0.00	n.d.	4.004976766e-07	0.00	0.95	0.00	0.00	0.00	0.00

[a]Data represent RPKM values if not specified otherwise
[b]not determined

Supplementary Table S6
Differential expression of cluster 3 genes in SkMCs and neurons versus astrocytes and fibroblasts

Gene ID	SkMCs n.i.[a]	Neurons n.i.	Astrocytes n.i.	Fibroblasts n.i.	*p* value
AC154586.1	7.08	0.81	63.09	75.90	1.52E-038
G6pdx	3.67	6.79	35.46	37.33	4.53E-035
Itpripl2	4.79	0.99	17.11	18.42	5.93E-032
Cnn2	94.85	0.49	182.23	194.16	7.85E-032
Ckap2	2.21	3.99	19.48	22.86	1.55E-031
Hdac1	11.76	4.51	38.21	39.02	6.36E-031
Casp8	10.88	0.08	19.58	18.70	9.96E-031
Osmr	10.60	0.98	28.61	33.37	1.31E-030
Suclg2	9.94	2.77	35.14	44.45	2.01E-029
Prdx1	89.64	17.77	229.96	252.73	3.90E-029
Rbpj	12.43	2.53	28.01	29.14	2.20E-028
Cald1	44.52	3.90	93.90	113.00	9.94E-028
Csf1	31.32	2.09	66.55	84.09	1.39E-027
Uaca	10.05	0.95	24.31	34.19	4.09E-027
Mmp19	6.44	0.27	13.02	13.79	5.17E-027
C1rb	3.15	0.01	9.17	9.22	9.29E-027
Sqrdl	5.41	0.11	12.11	13.82	9.80E-027
Col4a1	20.69	5.40	54.29	65.93	1.09E-026
Arpc1b	56.18	0.59	75.80	95.56	1.40E-026
Pcna	19.99	14.20	66.92	66.18	1.42E-026
Clic1	226.86	10.98	278.89	327.34	2.68E-026
Kctd10	32.31	10.47	76.00	81.20	1.02E-025
Ifitm2	174.77	11.26	297.81	382.21	1.18E-025
Igfbp4	24.01	27.63	106.69	126.46	2.49E-025
Mr1	9.80	0.35	14.09	15.62	2.63E-025
Sdf2l1	19.08	4.27	44.73	39.54	3.42E-025
Actn1	110.85	19.33	176.92	174.56	3.52E-025
Mobkl1b	25.21	3.88	42.11	39.45	8.11E-025
Serp1	39.76	8.79	71.32	73.61	9.17E-025
Slc43a3	1.98	0.20	8.00	10.14	1.02E-024
Gstm2	9.67	0.15	13.22	18.41	7.78E-024
Ccs	24.89	4.47	34.81	35.31	8.19E-024
Lass2	34.49	5.70	56.51	52.23	8.30E-024
Cbr3	8.18	2.03	17.03	20.38	9.88E-024
Gm5239	39.51	0.61	49.03	87.85	1.01E-023
Cpt1a	15.53	5.82	39.84	58.90	1.20E-023
Parva	19.13	4.39	36.25	33.71	1.60E-023
Zc3hav1	8.23	0.11	10.20	12.09	3.80E-023
Plekho2 (9 65402191..65427879)	12.94	2.33	24.11	34.69	4.66E-023
Adi1	9.65	3.12	20.29	23.17	5.52E-023
Socs3	10.43	0.73	14.39	18.99	6.26E-023
Sfrs9	22.35	5.93	42.48	48.21	6.85E-023
Syde1	11.28	2.04	17.11	17.17	7.29E-023
Tagln2	79.20	6.93	113.49	169.17	8.04E-023
Swap70	9.19	1.67	16.43	26.65	1.32E-022
Cyp39a1	5.65	1.05	11.23	11.79	2.31E-022
Pttg1	2.51	1.99	10.27	10.02	4.12E-022
Nde1	15.60	4.99	29.43	28.57	4.57E-022
Zfp36l2	28.72	5.04	47.41	64.79	4.64E-022
Tmem59	82.08	19.30	128.15	135.09	5.24E-022
Slc48a1	19.21	20.32	68.08	90.96	9.94E-022
AI462493	5.66	8.19	22.95	29.59	2.40E-021
Snx5	27.03	8.09	45.29	46.58	3.16E-021
Atl3	11.21	1.67	15.43	24.76	5.79E-021
Nxt1	10.27	1.97	15.52	24.78	8.44E-021
Plekhg3	3.77	0.14	7.65	7.48	9.51E-021
Cflar	6.77	0.74	10.87	10.14	1.11E-020
Ncapg2	1.53	1.29	7.48	7.82	1.64E-020
Col4a2	20.31	5.78	35.80	53.97	1.74E-020
Layn	10.28	1.80	13.93	24.14	1.76E-020
Mcfd2	21.03	4.90	31.48	36.79	2.70E-020
Elk3	13.55	3.44	22.13	33.09	3.63E-020
Cyp4f16	4.39	1.79	10.74	11.71	3.77E-020
Mov10	2.42	0.92	7.79	8.21	5.08E-020
Galk2	3.47	1.43	9.56	9.40	7.80E-020
Hsd17b12	37.31	13.44	63.62	64.31	8.05E-020
Fam125a	15.50	5.68	26.70	27.37	1.23E-019
AC158983.1	4.78	0.47	8.46	9.16	1.52E-019
Pole4	11.87	3.07	17.78	17.40	1.88E-019
Chpf2	12.28	1.97	14.12	20.72	2.76E-019
Pgd	19.22	10.86	43.79	41.67	2.83E-019
H6pd	7.85	1.08	10.27	11.26	3.33E-019
Glrx	19.04	14.91	54.32	49.24	3.38E-019
Tmem107	4.42	2.45	11.32	11.24	3.65E-019

Etfb	34.98	8.87	46.12	81.11	1.03E-018
Rbpms	1.59	0.65	6.00	8.18	1.61E-018
Cldn12	6.93	3.22	14.68	16.03	1.78E-018
Cchcr1	4.10	0.66	6.94	11.36	2.36E-018
Hn1l	9.32	11.96	29.94	36.61	2.76E-018
Pdcd6	62.83	16.57	78.79	83.83	3.15E-018
Tipin	6.50	5.64	16.65	23.84	3.41E-018
Ganab	28.93	18.88	64.69	88.15	5.01E-018
Ncapd2	3.27	13.85	17.88	24.11	5.69E-018
Ctsl	397.53	88.01	480.31	705.75	7.60E-018
Acer3	2.01	0.73	6.18	8.81	8.47E-018
Myo9b	14.87	6.63	26.37	27.50	8.96E-018
Lrrc42	15.18	6.76	27.51	26.51	1.65E-017
Sptlc2	22.42	10.67	41.00	40.30	1.78E-017
RP23-398D18.4	4.89	0.34	6.79	7.13	2.55E-017
1500011H22Rik	10.23	18.23	36.62	47.64	2.62E-017
Nsmce1	20.69	6.51	30.35	37.82	2.86E-017
Stt3a	25.09	6.14	30.45	39.71	3.57E-017
Capza1	41.47	13.05	56.85	60.66	3.64E-017
Amotl2	24.70	5.74	28.50	41.82	3.84E-017
Adc	5.56	3.22	12.46	16.30	3.88E-017
Gsr	6.81	5.30	15.96	20.74	4.74E-017
Mki67	2.65	11.52	14.51	17.44	4.97E-017
Hadh	11.45	4.39	16.06	28.33	5.05E-017
D10Ertd610e	20.28	7.65	30.98	46.94	5.85E-017
Dpm3	8.30	5.19	15.59	25.60	6.79E-017
Serhl	13.87	4.15	19.09	23.83	7.15E-017
Fhod1	2.61	0.06	5.28	6.91	7.24E-017
Cap1	72.88	59.67	195.91	173.33	9.41E-017
Prcp	10.62	3.99	17.13	19.23	9.87E-017
Mrpl27	12.18	5.43	18.08	31.28	1.54E-016
Adk	16.46	7.55	27.73	37.38	3.34E-016
Actn4	71.47	28.32	110.99	149.45	3.44E-016
Gbe1	6.58	1.93	10.44	10.58	5.33E-016
Rrm1	27.53	10.61	38.30	61.11	6.04E-016
Nucb1	19.49	10.14	36.65	34.79	6.08E-016
Hmgn1	24.56	15.90	46.04	68.27	1.25E-015
Imp3	24.67	7.68	28.67	45.88	1.87E-015
1700034H14Rik	6.16	1.22	7.79	11.53	1.96E-015
Mthfd1	15.37	9.41	29.01	37.79	2.39E-015
AI314976	6.69	4.36	14.29	16.27	2.83E-015
Sord	9.20	5.44	18.86	17.74	2.86E-015
Igf2r	13.72	5.06	19.06	19.52	2.90E-015
Retsat	5.00	1.55	8.31	11.11	3.34E-015
Pgm1	7.90	3.05	13.02	12.78	4.33E-015
Rag1ap1	9.24	2.90	12.95	13.67	4.70E-015
Tmx1	14.81	7.24	25.07	24.31	4.82E-015
Nrp1	10.75	15.67	29.95	42.12	5.33E-015
Gins2	2.04	1.85	6.76	8.82	6.35E-015
Polr2g	35.48	19.39	58.76	85.85	6.59E-015
Cd2ap	5.30	2.28	9.71	12.20	7.02E-015
Paox	4.17	1.22	7.46	9.78	7.26E-015
Tulp3	5.10	2.72	11.53	10.35	9.18E-015
Dennd4c	3.37	0.89	6.57	8.30	1.07E-014
Ube2l3	1.46	1.97	6.30	6.68	1.29E-014
Slc35b2	17.25	7.23	27.72	25.32	1.43E-014
Slc35a4	32.15	11.29	40.78	46.06	2.19E-014
Hmbs	10.97	6.03	17.29	26.09	2.40E-014
Rdm1	2.25	0.37	4.60	6.65	2.54E-014
Rpn2	59.19	19.81	71.86	83.70	2.58E-014
Nusap1	1.25	2.54	6.32	8.19	2.90E-014
Sephs2	9.91	12.27	24.23	34.69	3.12E-014
Sec61b	20.97	8.96	28.22	42.57	3.75E-014
Immp1l	11.91	6.18	24.57	19.37	4.15E-014
Ctps2	6.99	3.71	12.05	16.74	4.59E-014
Nfatc1	3.17	1.06	6.52	7.83	5.42E-014
Spata24	3.53	1.27	7.07	7.09	6.22E-014
Fam58b	13.55	4.69	17.01	18.38	7.72E-014
Actg1	621.20	437.62	1009.65	1678.86	7.98E-014
Smc2	2.95	3.93	8.74	11.92	1.07E-013
Ikbip	7.01	1.89	9.07	11.17	1.22E-013
Abcb1b	3.18	0.35	5.57	5.28	1.34E-013
Shfm1	185.18	69.58	229.22	315.86	1.55E-013
D10Wsu52e	59.90	25.51	70.03	118.58	1.67E-013
Mesdc2	14.00	8.41	23.82	30.55	1.81E-013
Kdelc2	6.03	1.97	8.01	12.29	1.83E-013
Ccdc61	2.25	1.84	6.71	7.35	2.03E-013
D15Ertd621e	17.05	7.42	20.19	33.74	2.49E-013

Klhl9	17.43	6.79	22.38	24.58	2.61E-013
Pdk3	7.86	3.45	9.94	17.16	2.71E-013
Pml	5.27	2.84	10.22	10.42	2.95E-013
Atp9b	12.83	5.87	18.65	21.14	3.33E-013
Coq2	9.25	4.57	13.74	18.90	6.67E-013
Evc	2.91	0.62	5.13	6.55	6.75E-013
Il1rap	1.14	0.79	4.42	5.24	8.63E-013
Psmd5	14.03	7.06	21.18	24.68	9.13E-013
Dzip1l	3.10	2.54	8.06	8.93	9.34E-013
Rpa2	4.59	2.18	8.68	9.52	9.85E-013
Gnai3	37.01	14.89	44.40	63.87	1.00E-012
Nab1	11.33	6.24	17.79	21.76	1.30E-012
Ripk3	2.15	0.00	3.90	4.89	1.34E-012
Ttc7	3.54	0.54	4.94	6.97	1.47E-012
Gart	13.36	5.86	15.87	24.62	1.93E-012
Efna4	4.68	1.16	6.76	7.33	2.87E-012
Txndc12	11.18	19.46	31.20	35.48	3.00E-012
Pvrl2	8.95	6.06	16.55	17.13	3.62E-012
Mgat2	26.71	10.52	32.08	38.72	4.09E-012
Cep192 (18 68001178..68044824)	1.90	2.48	6.30	8.21	4.33E-012
Arsa	11.72	9.94	21.31	30.23	4.40E-012
Tmem164	4.03	3.88	10.40	9.99	4.83E-012
Adprhl2	6.28	3.97	9.48	15.72	5.39E-012
Akr1a4	165.64	74.16	217.85	267.27	6.62E-012
Ttf2	2.47	0.47	3.94	6.35	6.78E-012
Taldo1	49.87	26.78	78.08	75.17	7.74E-012
Rbm7	15.02	10.74	26.98	28.40	8.35E-012
Wdr61	10.38	5.14	13.69	19.90	8.92E-012
Odc1	79.16	63.25	153.93	150.11	1.24E-011
Sod1	198.70	150.23	361.75	415.86	1.31E-011
Tctex1d2	5.40	1.24	6.12	9.02	1.38E-011
Mat2b	9.39	4.02	13.11	14.83	1.45E-011
2310061C15Rik	5.02	1.62	7.22	9.16	1.78E-011
Mta2	15.68	14.97	31.67	38.17	1.84E-011
Dock7	17.87	8.93	27.69	25.21	1.86E-011
0610011F06Rik	11.98	12.36	22.91	33.27	2.04E-011
Slc40a1	1.96	0.18	4.13	3.79	2.28E-011
Isoc1	7.87	7.48	13.36	21.36	2.48E-011
Nat2	3.41	0.25	4.22	4.60	2.53E-011
Rnaseh2c	7.32	5.43	14.11	14.33	2.88E-011
Phtf1	3.25	2.78	9.14	7.79	3.45E-011
Sumf2	5.49	4.11	9.16	14.42	3.61E-011
Impa1	11.88	5.14	15.58	17.02	4.07E-011
4930562F07Rik	2.70	0.24	3.63	5.69	4.46E-011
Wee1	4.62	1.63	7.65	7.23	4.57E-011
1110059E24Rik	9.81	4.69	11.45	18.37	4.69E-011
Cox4nb	27.14	19.86	54.76	47.11	5.52E-011
Slc25a39	33.91	17.90	47.46	57.50	7.11E-011
Cpne1	8.69	6.42	14.54	19.42	8.37E-011
Ppfia1	6.92	3.71	12.54	11.09	8.59E-011
Itpripl1	0.41	1.50	3.73	3.81	8.81E-011
Ppp2r1b	4.76	4.01	9.84	11.68	8.88E-011
Txnrd1	35.78	28.50	64.64	74.06	9.50E-011
Nubp2	20.99	11.52	29.68	36.92	9.66E-011
Ube2t	0.56	0.92	3.44	3.76	1.09E-010
Clns1a	18.03	17.99	34.68	45.55	1.12E-010
Adamts10	1.78	2.63	6.08	6.96	1.13E-010
Gins4	16.78	8.48	22.70	26.35	1.28E-010
Fgf2	1.80	0.24	3.52	3.82	2.28E-010
Hebp1	6.43	5.15	14.87	12.05	2.35E-010
RP23-50E4.1	5.00	6.07	14.40	11.55	2.49E-010
Psenen	39.48	21.57	60.10	55.75	3.57E-010
Psmc3ip	2.48	1.49	5.39	6.51	3.83E-010
Cc2d1b	8.28	3.65	9.88	14.41	3.95E-010
Ddost	48.03	33.48	94.04	76.56	4.20E-010
Arhgef1	7.81	4.08	11.80	12.45	4.99E-010
Ahcyl1	22.27	16.35	37.45	41.55	5.73E-010
Crebzf	2.92	2.49	7.57	6.92	7.66E-010
Zw10	5.17	2.61	8.61	8.56	1.06E-009
Stx5a	14.24	16.75	29.84	34.71	1.08E-009
Arl6ip6	3.92	1.08	5.08	6.79	1.11E-009
Dsn1	1.82	1.02	4.50	5.11	1.43E-009
Coro1b	51.45	25.28	61.88	79.28	1.48E-009
Nudt6	3.23	1.28	5.22	6.68	1.66E-009
Rbl1	1.83	0.93	4.42	4.69	1.91E-009
Metapl1	1.10	1.29	4.16	4.38	2.11E-009
Adss	9.13	9.87	18.84	19.44	2.27E-009
Trib1	2.98	3.75	6.46	10.10	2.72E-009

Acsf3	3.85	2.64	7.13	9.12	3.42E-009
Nme7	8.24	7.65	17.15	15.86	3.86E-009
E2f1	8.06	4.80	11.83	14.83	4.68E-009
Atox1	82.08	74.60	151.05	152.01	5.68E-009
Hmgb1	2.26	4.25	7.32	7.40	6.23E-009
Rhobtb3	5.56	3.40	9.93	9.43	6.31E-009
Dcp2	2.43	3.41	7.29	6.96	7.72E-009
Tmem106b	17.56	11.38	23.13	32.79	7.85E-009
Hist1h1e	2.27	0.18	2.94	4.31	8.72E-009
Acap2	7.71	6.35	14.28	14.14	9.63E-009
Tsen34	22.51	16.08	33.61	42.60	9.90E-009
Ubr7	10.06	7.36	18.53	16.30	1.03E-008
Xpo5	14.80	8.86	20.47	23.74	1.07E-008
Crlf2	4.60	9.75	12.12	14.47	1.07E-008
Dph1	5.97	2.55	7.42	10.08	1.10E-008
Rab9	17.03	10.73	24.39	27.79	1.34E-008
Rgs19	7.18	13.66	16.72	20.42	1.82E-008
Smox	6.94	8.33	13.33	17.99	1.97E-008
Ppp4c	34.43	21.74	52.11	49.78	1.99E-008
Coq6	4.78	1.79	5.32	8.41	2.09E-008
Svep1	0.20	0.03	1.99	2.33	2.22E-008
Rfwd3	3.18	1.95	5.38	7.67	2.27E-008
Rfc2	16.89	16.16	29.30	36.40	2.78E-008
Gemin7	12.70	9.58	20.02	22.72	3.19E-008
Gas2l3	0.70	0.68	2.72	3.59	3.57E-008
1300018J18Rik	6.82	3.03	8.00	10.77	4.06E-008
Ermp1	3.02	3.23	6.39	8.87	4.10E-008
Prpf31	6.77	6.04	11.29	15.36	4.46E-008
Mpnd	20.72	13.24	29.65	31.70	4.50E-008
Rpn1	72.90	42.41	89.03	118.92	4.80E-008
Ncbp2	18.94	15.26	30.50	35.55	4.83E-008
Pdia3	121.25	99.01	183.22	244.69	5.05E-008
Vrk3	5.36	6.64	12.89	11.67	5.86E-008
Rnf138	3.26	1.78	5.70	6.46	6.46E-008
RP23-402K12.2	1.39	0.62	3.55	3.33	6.59E-008
Txnrd2	3.34	2.00	5.99	6.78	6.68E-008
Klc3	2.14	0.11	2.83	3.30	6.70E-008
Vmac	3.88	3.68	9.49	7.87	6.85E-008
Clp1	3.63	1.71	5.55	6.84	8.33E-008
Tmem120a	13.83	9.44	17.38	25.33	8.55E-008
Txlna	11.80	7.50	15.55	20.01	1.11E-007
Cth	0.75	1.10	3.30	3.41	1.13E-007
Inpp5b	5.59	4.63	11.44	9.84	1.14E-007
AC167249.3	58.36	33.13	70.50	89.71	1.15E-007
Eno1	140.43	90.91	174.31	244.60	1.21E-007
C230013L11Rik	0.37	0.00	1.95	2.35	1.36E-007
2310045N01Rik	5.28	8.36	12.38	13.65	1.44E-007
Fam33a	3.88	3.85	7.13	10.09	1.50E-007
Hist1h2bj	1.37	0.13	2.30	3.46	1.50E-007
Dnmbp	4.31	2.03	5.11	8.25	1.60E-007
Sfrs13a	6.83	5.92	12.26	13.16	1.63E-007
Nampt	12.16	15.44	24.61	26.08	1.84E-007
Wdr67	2.89	0.97	3.53	5.70	3.15E-007
Mex3c	11.20	9.28	17.00	21.42	3.31E-007
Cttn	59.48	38.58	85.93	82.25	5.33E-007
A430107O13Rik	1.28	0.04	2.01	3.23	5.50E-007
Eif3k	81.30	85.20	130.43	178.66	6.11E-007
Lipe	0.93	0.82	3.21	3.13	6.89E-007
Mrpl35	2.04	3.12	4.73	7.19	7.24E-007
Bcl2l12	1.97	0.30	2.45	3.69	7.46E-007
Eftud2	26.37	15.74	33.17	37.98	8.45E-007
Ppp1cc	38.74	27.31	51.92	66.08	9.50E-007
Casd1	5.41	4.64	8.80	11.31	1.39E-006
A930004D18Rik	0.79	1.25	3.13	3.52	1.46E-006
Cdc42se1	27.62	21.76	41.66	46.81	1.61E-006
Rpusd4	3.82	1.50	5.24	5.63	1.71E-006
Slc30a7	6.53	4.97	11.13	10.68	1.72E-006
Aspm	0.49	1.55	3.03	3.30	1.81E-006
Sf3b5	39.29	27.39	54.12	62.65	1.86E-006
Tysnd1	7.57	8.27	14.40	15.14	1.88E-006
2310014H01Rik	19.08	11.80	24.49	26.71	2.30E-006
Rcbtb1	4.66	3.19	7.32	8.93	2.56E-006
4930503L19Rik	3.55	1.89	5.22	6.56	2.60E-006
Tor2a	5.94	3.78	8.95	9.65	3.11E-006
Tubgcp5	4.21	2.16	6.24	6.23	4.01E-006
Elavl1	17.12	11.06	22.66	22.92	5.01E-006
BC003267	3.19	1.73	4.20	6.41	5.46E-006
Zkscan17	5.38	4.62	7.96	11.25	5.48E-006

Ampd2	7.76	11.18	15.92	17.00	5.54E-006
Toe1	2.42	1.58	4.24	5.62	5.72E-006
Wrn	2.05	1.04	3.83	3.89	6.01E-006
Tinf2	4.21	2.69	6.41	7.88	6.14E-006
2410022L05Rik	15.13	10.25	19.25	23.75	6.83E-006
Siah1b	2.57	0.99	3.24	5.03	8.65E-006
Hps1	1.85	2.03	4.63	4.96	9.65E-006
Zfp93	0.80	2.08	3.25	4.09	1.12E-005
Nudt1	4.66	3.36	7.02	9.04	1.18E-005
Taf6l	2.92	4.64	7.18	7.44	1.34E-005
Lrwd1	3.87	1.95	5.72	5.69	1.64E-005
6720463M24Rik	1.13	1.07	2.63	3.97	1.88E-005
Ubxn8	5.07	4.82	9.27	9.58	2.23E-005
Coq7	2.63	2.66	4.70	6.76	2.45E-005
Mms19	11.03	7.24	13.35	17.19	2.81E-005
Snip1	8.32	5.08	9.81	12.82	3.18E-005
Glt8d3	4.51	2.74	6.12	7.95	3.34E-005
RP23-103L13.5	1.54	0.05	1.94	2.68	3.84E-005
Gm10941	0.42	0.38	1.97	2.17	4.31E-005
3200002M19Rik	1.72	2.21	4.88	4.41	4.94E-005
Fam195a	6.49	4.91	9.04	11.43	5.34E-005
Tlcd1	4.85	2.53	6.43	6.58	6.03E-005
Mier1	2.60	1.26	4.60	4.02	6.72E-005
Cenpp	1.43	1.11	2.69	4.12	8.34E-005
Prkcsh	52.58	46.87	75.91	92.08	9.17E-005
Rbm39	17.18	11.70	22.27	22.78	9.94E-005
Cebpg	6.69	5.53	10.96	10.60	0.00010555
Gabpb2	2.00	0.80	2.58	3.98	0.00012689
Casp2	1.82	1.27	2.92	4.61	0.00012813
Prkci	9.95	10.15	16.24	18.14	0.00013832
Gfer	8.39	8.77	15.02	14.40	0.00016506
Polr2l	9.79	7.82	15.60	14.27	0.00016955
Fastkd3	2.00	1.04	3.70	3.39	0.0001744
Mitd1	2.62	1.50	4.79	4.18	0.00022961
Eri1	5.49	3.56	7.23	8.98	0.00024001
Pigm	3.02	1.48	4.46	4.38	0.00031114
Ccm2	11.46	8.36	13.92	18.02	0.00031725
AI413582	10.51	10.40	18.53	16.56	0.00032858
Trmt2a	5.10	5.88	10.04	9.45	0.00039662
Zfp395	4.30	3.93	6.81	8.65	0.00043814
Hjurp	4.08	6.46	8.60	9.18	0.00048684
Commd10	12.91	9.47	15.99	19.59	0.0004952
Thumpd2	1.29	0.80	2.49	3.34	0.00052961
Mtmr12	5.04	3.27	7.17	7.40	0.00060994
Mpst	14.81	18.84	26.10	26.43	0.0007211
Rab23	2.89	3.52	5.21	6.98	0.00092061
Ttrap	4.12	3.74	7.08	7.41	0.00097063
RP23-191N15.1	0.50	0.08	1.30	1.78	0.0012068
A630089N07Rik	0.69	0.72	2.04	2.76	0.0013885
Flywch1	11.12	13.89	17.89	21.39	0.001569
Gli3	1.70	1.19	3.60	3.09	0.0019379
1110007A13Rik	7.99	8.12	13.81	12.85	0.0020563
Wrap53	3.22	2.02	5.38	4.67	0.0027326
Rpl13-ps3	0.68	0.10	1.24	2.00	0.0034894
Csrp2bp	5.60	4.88	9.37	8.64	0.0036307
Ung	0.66	2.01	2.44	3.18	0.0042949
Vrk2	0.97	0.13	1.75	1.53	0.0060928
Zfp472	1.38	0.49	2.22	2.64	0.0062025
Churc1	1.17	0.22	1.78	1.94	0.0062298
Ino80b	10.96	8.34	14.82	14.70	0.0071428
Pgls	9.98	7.75	11.99	15.32	0.0081312
AC162689.2	3.01	1.50	3.60	4.58	0.0093139
AY074887	1.06	0.22	1.43	2.30	0.0093721
Dclre1a	2.95	1.57	3.77	4.60	0.0097616

[a]Data represent RPKM values if not specified otherwise

Supplementary Table S7
Differential expression of cluster 15 genes in SkMCs and neurons versus astrocytes and fibroblasts

Gene ID	SkMCs n.i.[a]	Neurons n.i.	Astrocytes n.i.	Fibroblasts n.i.	*p* value
Cd109	8.16	0.26	50.92	49.71	2.12E-042
Dock8	0.53	0.09	15.82	14.21	2.91E-040
Cav2	3.85	0.19	20.88	17.72	1.49E-035
Mgmt	0.00	0.47	17.50	12.49	2.06E-035
S1pr3	2.38	1.87	106.85	68.75	2.40E-035
Gtse1	1.10	1.91	13.98	14.77	4.88E-034
Id3	3.40	1.07	74.62	41.42	1.22E-032
Tap2	3.32	0.50	21.79	15.94	3.69E-032
H2-L	28.03	8.55	288.56	181.19	2.49E-031
Rbp1	2.46	5.60	112.04	46.82	1.19E-030
Cmtm7	8.88	0.03	32.04	23.81	8.08E-030
Acot1	0.64	6.37	63.69	26.68	1.94E-029
F3	3.18	5.08	78.37	32.00	2.33E-029
Fam43a	1.28	1.23	17.51	11.53	4.53E-029
Cd300lb	0.04	0.01	13.06	6.22	6.45E-029
Krcc1	1.36	0.49	14.67	9.45	6.23E-028
Ptx3	0.14	0.01	9.88	6.31	3.36E-027
Psrc1	1.29	2.23	17.06	11.72	4.68E-027
Idh2	43.10	5.62	116.51	95.36	4.79E-027
Bicc1	2.88	0.61	11.52	10.26	6.09E-027
Ass1	11.64	4.20	68.58	41.20	7.71E-027
Id1	5.72	1.31	36.31	16.17	9.79E-027
Spp1	490.95	2.87	1827.30	1051.07	1.15E-026
Pnp1	9.37	1.05	51.31	21.08	1.18E-026
Rgs16	2.57	3.40	15.14	14.41	1.50E-026
Sdc4	37.09	3.53	109.54	78.84	4.44E-026
Slc16a1	13.53	2.33	33.59	27.86	1.74E-025
Ugt1a7c	0.06	0.20	8.07	5.82	2.71E-025
Cmtm3	12.16	3.56	43.79	31.82	3.89E-025
Anxa5	116.22	12.77	449.18	229.65	6.27E-025
S1pr2	8.22	0.33	22.57	14.12	6.98E-025
Frmd8	10.10	1.19	24.89	18.92	7.07E-025
Ftl1	136.99	347.97	1980.92	866.02	7.19E-025
Ormdl2	11.09	1.00	25.38	19.07	1.10E-024
Gm9853	3.09	1.33	16.81	11.23	1.30E-024
Gsta4	9.02	15.05	95.13	45.31	1.60E-024
Sipa1	6.18	0.93	20.92	13.00	1.60E-024
Mapkapk3	0.03	0.58	8.72	5.96	2.22E-024
Ucp2	16.41	0.48	40.97	24.86	2.53E-024
Fxyd5	3.51	1.30	19.26	8.27	7.26E-024
Adm	1.89	0.75	11.65	8.04	7.70E-024
1810037I17Rik	26.12	7.92	75.16	59.30	1.17E-023
Clcf1	4.57	0.17	12.35	9.70	1.24E-023
Plin2	39.71	0.74	76.82	45.49	1.53E-023
Tpcn2	2.28	0.12	7.62	7.34	2.13E-023
Lrig1	6.20	7.79	26.70	24.50	2.83E-023
Eml3	4.27	1.72	15.19	12.49	4.33E-023
Pdia4	45.83	17.12	148.85	110.48	4.71E-023
Plekhf1	7.54	0.11	18.41	10.36	7.61E-023
Lhfpl2	4.00	1.85	19.08	11.06	7.68E-023
Vim	1067.93	83.24	2736.81	1434.03	8.50E-023
Tmem173	7.70	0.12	16.15	11.07	1.53E-022
Lhfp	27.08	5.70	84.89	49.94	1.55E-022
Aldh7a1	8.60	14.71	73.88	29.99	1.67E-022
Golm1	3.95	4.43	26.90	11.26	2.02E-022
Ccnb2	4.56	2.04	19.55	11.72	2.38E-022
Cyb5	8.12	12.63	49.45	36.31	3.39E-022
Trim25	4.52	0.29	14.72	8.74	3.87E-022
Slc19a2	2.21	2.45	11.44	10.16	3.98E-022
Tnfrsf1a	34.17	5.82	72.56	54.98	4.33E-022
Tspan4	32.75	4.60	64.82	44.99	3.45E-021
Naglu	9.61	2.10	25.26	15.89	3.97E-021
M6pr	28.86	4.10	63.40	39.55	4.35E-021
Zfp36	7.40	0.45	14.61	10.49	6.44E-021
Il11ra1	1.48	1.18	11.00	6.43	1.04E-020
Gstt3	0.28	0.11	7.51	3.83	1.29E-020
Arhgap19	1.01	1.45	10.86	5.90	1.49E-020
Dhrs4	10.96	2.05	27.00	14.99	1.66E-020
AC157563.2	6.03	1.66	19.36	8.48	1.70E-020
Gpx7	0.03	1.43	8.26	5.45	1.84E-020
Fhdc1	0.76	1.46	7.97	6.75	1.98E-020
Gla	3.24	1.86	12.85	9.97	2.04E-020
Dusp6	7.78	6.16	32.59	21.56	2.22E-020
Cmtm4	4.78	6.99	21.61	18.20	2.41E-020

Ripk1	6.85	2.10	18.00	13.67	2.42E-020
H2-M3	2.65	0.10	8.56	6.76	3.19E-020
Tmem179b	10.26	2.79	22.52	17.32	3.58E-020
Soat1	10.00	1.72	22.74	11.63	4.39E-020
B9d1	4.96	2.63	19.73	8.60	5.05E-020
Apbb1ip	2.79	0.15	8.35	6.89	5.36E-020
Spc25	1.31	0.84	6.30	6.27	9.53E-020
Rnf149	10.98	3.63	32.45	17.17	9.63E-020
Apob48r	1.09	0.08	8.41	4.13	1.54E-019
Gpr137b	6.03	1.53	17.56	10.02	1.63E-019
AC113595.2	2.88	0.29	10.62	5.89	1.67E-019
Tsku	3.41	2.07	13.14	9.99	1.75E-019
Cyfip1	31.31	10.08	88.49	37.91	1.77E-019
Mpp1	11.35	4.20	29.31	20.92	2.36E-019
1810063B05Rik	10.33	2.82	20.47	16.38	3.17E-019
Myd88	5.81	1.56	15.37	10.89	4.46E-019
Wnt5a	0.03	1.37	8.51	3.92	4.74E-019
Tmem106c	5.10	11.68	36.63	19.19	5.88E-019
2610034B18Rik	8.01	2.51	20.93	8.99	7.51E-019
1110020G09Rik	7.41	5.00	21.04	17.73	7.61E-019
Slc25a10	5.57	3.00	16.81	13.24	7.87E-019
Acaa1a	20.28	8.20	58.89	23.72	8.45E-019
Chek2	1.15	0.60	6.08	5.62	1.07E-018
Mif4gd	4.86	2.03	16.24	9.90	1.31E-018
Cdkn3	0.73	0.53	6.47	4.81	2.15E-018
Gm5617	3.13	1.71	13.69	6.31	2.32E-018
Siae	1.02	4.32	13.25	6.02	3.87E-018
Sh3bp1	4.51	3.11	14.98	12.03	4.06E-018
Psmb10	7.42	5.42	27.66	12.21	4.67E-018
Prps2	5.36	3.93	20.11	8.21	5.94E-018
Ppm1m	3.21	0.58	8.67	7.14	8.66E-018
Tmem98	20.34	8.06	47.11	34.86	1.04E-017
Stard3nl	10.50	6.93	35.29	21.19	1.04E-017
Rab20	0.99	0.04	7.09	3.29	1.11E-017
Pard6b	0.19	0.71	4.70	4.42	1.22E-017
Sft2d1	8.24	2.33	18.86	11.92	1.34E-017
Foxc1	2.52	0.12	7.04	5.58	1.87E-017
Gng12	17.32	6.92	41.56	28.06	3.28E-017
Tjp2	4.52	7.50	20.80	15.37	4.15E-017
Cklf	4.07	0.71	9.17	7.62	4.47E-017
Orai3	6.25	2.78	17.79	6.95	5.61E-017
Epb4.1l2	25.22	13.06	72.76	37.87	7.00E-017
AC148011.4	2.61	0.00	7.91	4.83	7.72E-017
Pqlc3	3.50	0.22	9.42	5.27	8.13E-017
Eif2ak2	5.91	2.26	16.38	6.97	1.05E-016
Tmem128	6.99	2.57	18.13	9.13	1.21E-016
Tmem203	2.53	0.58	7.33	6.08	2.63E-016
RP23-153F8.11	1.49	0.21	6.74	4.19	2.94E-016
Ptplb	8.97	14.73	43.53	20.43	3.57E-016
Tm7sf3	6.23	7.05	25.06	12.87	5.00E-016
Nxt2	1.49	2.10	10.22	4.15	5.16E-016
Btg2	8.02	2.36	17.06	10.64	7.10E-016
Dapp1	0.08	1.90	7.35	3.05	7.84E-016
Plagl1	0.00	2.96	7.92	4.05	8.62E-016
Pkd2	5.06	1.92	13.79	8.17	8.67E-016
Gja1	16.68	18.97	65.06	35.28	1.42E-015
Blvra	3.29	0.87	9.86	5.60	2.06E-015
Rhoj	3.28	0.36	7.09	5.75	2.51E-015
Dram1	4.45	0.23	8.85	4.48	2.72E-015
Glb1	16.16	8.69	40.63	18.03	3.91E-015
Hscb	7.10	1.91	14.70	8.31	4.63E-015
Shisa5	24.40	14.99	66.73	31.89	4.71E-015
Slc39a11	3.23	4.00	14.67	7.87	5.94E-015
Npr3	0.27	1.97	6.36	4.57	7.00E-015
Sh3bgrl	27.54	18.00	74.85	31.85	7.06E-015
Slc35b4	3.59	0.94	9.30	6.50	7.26E-015
Bax	56.05	27.15	128.61	84.97	7.33E-015
Tmem216	3.71	2.17	10.82	8.37	8.08E-015
Pafah2	2.01	1.64	9.33	5.57	1.30E-014
Jak3	0.73	0.80	6.41	3.45	1.44E-014
Naip2	0.57	0.07	5.32	2.80	1.58E-014
Edem1	5.37	4.00	16.38	11.11	1.66E-014
Pkdcc	2.82	4.55	14.11	6.11	1.74E-014
Rilpl2	11.48	4.83	24.32	13.04	1.90E-014
Spred1	7.11	7.22	23.89	14.64	2.09E-014
Gm12942	0.04	1.09	5.83	2.88	2.24E-014
Irak4	3.50	0.14	7.37	4.29	2.79E-014
Gcsh	13.48	20.09	50.60	36.63	3.36E-014

Mtmr10	4.43	2.01	10.44	8.65	3.67E-014
Rcbtb2	7.73	8.54	24.71	18.23	4.16E-014
Dock1	4.81	2.83	12.71	9.59	5.07E-014
Trim56	4.36	0.72	8.84	5.90	7.85E-014
Tbc1d2b	2.81	2.21	10.59	4.43	1.15E-013
Plekha4	0.44	0.36	5.12	2.98	1.31E-013
Ctf1	2.96	0.22	7.13	3.41	1.32E-013
H2afv	26.30	17.49	60.77	47.54	1.50E-013
Ift80	2.76	1.13	8.98	3.72	1.52E-013
Prr5l	0.03	0.14	4.29	2.02	1.93E-013
Glt8d4	1.45	0.29	4.96	4.21	2.23E-013
Tubb2c	101.94	82.67	283.41	165.94	5.14E-013
Hist1h2ac	0.97	0.16	4.27	3.39	1.35E-012
Ogg1	3.64	3.36	11.27	8.61	1.45E-012
Cdsn	0.66	0.14	4.46	2.87	1.64E-012
Tpra1	5.56	3.78	14.87	8.76	4.27E-012
Hspb6	7.50	5.41	19.10	10.69	5.07E-012
Pex7	4.73	2.10	10.91	5.25	7.46E-012
Cdk5rap2	4.81	2.71	11.43	7.94	1.23E-011
AC118255.1	2.13	2.29	8.71	5.24	2.67E-011
Stt3b	24.83	17.02	53.14	39.64	3.70E-011
Lgr4	2.75	2.37	8.68	6.62	5.14E-011
Sec11c	4.40	2.83	11.21	6.02	1.07E-010
Nfxl1	2.96	1.19	7.29	5.05	1.17E-010
Abcc4	0.78	1.67	6.09	2.91	1.18E-010
Rnf114	10.16	8.88	25.43	15.04	1.40E-010
Mkx	0.13	1.55	4.28	3.22	1.45E-010
Mboat1	1.25	0.21	4.79	2.66	1.46E-010
Spcs2	21.12	14.38	45.29	29.95	1.65E-010
Prkd2	1.96	0.83	6.26	2.58	1.66E-010
Fam55d	0.22	1.38	5.00	2.21	1.72E-010
Tmem20	0.00	0.13	2.32	2.07	2.04E-009
Ctbs	2.35	0.74	5.91	2.79	3.44E-009
Fbxo33	2.46	1.48	7.06	3.91	3.61E-009
Chst7	1.01	0.49	3.65	2.84	2.04E-008
Mfsd9	0.53	0.70	3.78	2.24	3.05E-008
RP23-138L21.8	0.36	0.00	3.06	1.51	3.38E-008
Tapbpl	1.49	0.14	3.68	2.34	3.75E-008
Tmem171	0.19	0.00	2.93	1.23	4.66E-008
Cnpy3	1.89	1.18	5.40	4.06	5.33E-008
Hist1h2bg	0.84	0.10	3.29	2.11	5.82E-008
Srd5a3	2.05	1.56	6.27	3.81	5.84E-008
Zfp367	1.99	1.44	5.64	4.41	7.69E-008
E130306D19Rik	0.23	0.41	2.49	2.33	8.68E-008
Reck	1.08	0.65	4.14	2.71	8.81E-008
Cyp4f13	0.94	0.51	3.98	1.86	1.11E-007
RP23-344D2.4	0.46	0.18	2.34	2.28	1.26E-007
4632434I11Rik	0.41	0.76	3.36	2.40	1.89E-007
Hoxa2	0.13	0.00	1.58	1.67	1.93E-007
Sys1	4.99	4.08	11.15	7.71	2.31E-007
Eif4ebp3	1.40	0.25	3.66	1.48	2.73E-007
6720489N17Rik	0.30	0.18	2.86	1.77	2.80E-007
RP23-148P12.3	1.66	0.16	3.32	2.60	3.58E-007
Slc25a15	2.33	1.14	5.59	3.62	5.08E-007
Gyk	0.45	0.65	3.42	1.81	6.34E-007
Calhm2	0.03	0.13	2.49	1.34	6.85E-007
Meis1	1.43	1.28	4.68	3.68	6.91E-007
Ccbl2	0.57	0.73	3.31	2.48	8.72E-007
Mitf	1.62	0.15	3.41	1.80	1.95E-006
Nudt7	0.00	0.29	2.50	1.17	3.88E-006
Lrdd	0.43	1.32	3.59	1.84	4.29E-006
AC163446.1	0.58	0.33	3.04	1.73	5.02E-006
AC115896.3	0.42	0.68	2.90	2.28	6.44E-006
AC107236.1	0.71	0.14	2.90	1.52	9.60E-006
6720457D02Rik	0.76	0.21	2.79	2.00	1.02E-005
Nudt12	0.38	0.15	2.14	1.72	1.84E-005
Gm8815	0.26	0.09	1.81	1.48	2.49E-005
Gm10374	0.53	0.75	3.23	1.56	2.97E-005
Tgif2	0.56	0.23	2.53	1.69	6.62E-005
RP23-9F23.4	1.24	0.94	3.78	1.98	0.00011339
Mnda	1.21	0.01	2.49	1.65	0.00012041
Col22a1	0.04	0.35	1.89	1.32	0.00013576
Ifi205	0.19	0.02	1.94	0.84	0.00014324
Hus1	1.56	1.28	4.35	2.90	0.00015072
Rffl	1.33	0.72	3.27	2.65	0.00020288
1200009I06Rik	0.88	0.17	2.71	1.26	0.00020544
Stard6	0.36	0.69	2.73	1.36	0.00047577
Mpp7 (18 7348765..7348895)	0.31	0.50	1.94	1.74	0.00050573

4833427G06Rik	0.89	0.06	2.16	1.57	0.00051966
RP23-337P9.3	1.30	1.48	3.97	2.63	0.0014629
AC136146.4	0.70	0.56	2.70	1.77	0.0017007
Crem	1.07	0.92	3.32	2.08	0.0018744
Prx	0.59	0.29	2.01	1.65	0.0020394
Hist1h3d	0.34	0.18	1.82	1.17	0.0024701
RP23-203J4.1	0.68	0.00	1.83	1.00	0.0034398
Slc39a8	0.49	0.02	1.66	1.01	0.0040976
6030446N20Rik	1.21	0.73	3.13	1.36	0.00453
RP23-295E4.3	0.13	0.36	1.66	1.00	0.0087898

[a]Data represent RPKM values if not specified otherwise

Supplementary Table S8
Differential expression of cluster 24 genes in SkMCs and neurons versus astrocytes and fibroblasts

Gene ID	SkMCs n.i.[a]	Neurons n.i.	Astrocytes n.i.	Fibroblasts n.i.	*p* value
Ablim3	30.88	33.19	0.09	0.03	6.21E-048
Rtn2	60.33	53.21	5.38	0.22	3.30E-041
Fgf11	17.74	16.21	1.30	0.08	1.11E-040
Cdh13	54.77	41.50	3.77	0.00	1.71E-037
Dlgap4	57.76	60.25	11.55	3.79	5.86E-037
Enho	26.84	25.84	7.53	0.35	7.74E-037
Foxo6	19.10	29.88	0.48	0.04	2.96E-036
Xist	25.57	25.49	11.91	0.00	1.14E-034
Cbfa2t3	8.54	10.57	0.02	0.00	1.88E-033
Cacna2d1	41.23	32.38	2.06	5.25	1.94E-033
Lrrn1	16.87	24.69	3.10	0.00	5.33E-033
Tmem158	24.59	20.97	1.56	4.47	5.84E-033
Egr3	21.44	26.82	0.52	5.30	7.57E-033
Sox11	10.76	15.39	0.86	0.00	1.93E-032
Mmp15	28.81	28.27	18.12	0.05	2.50E-032
Akap6	19.01	25.94	4.62	0.11	2.84E-032
Pde4dip	38.33	30.83	4.73	3.77	6.29E-032
Cobl	14.89	12.37	2.56	0.01	8.31E-032
Ndrg4	77.69	117.53	9.51	6.31	1.23E-031
Ttc9	33.45	28.29	13.24	0.02	3.20E-030
Ppp1r14c	19.22	15.24	5.45	0.00	4.95E-030
Armcx4	14.63	20.18	2.47	1.60	6.30E-030
Tmem132a	59.49	65.47	9.56	13.85	8.15E-030
Gm15440	17.32	8.89	1.06	0.00	9.30E-030
Ndufb4	49.72	44.33	9.97	7.98	6.08E-029
Shc2	14.94	18.34	1.03	4.14	7.66E-029
Map1lc3a	202.17	148.70	30.50	21.74	2.48E-028
C630004H02Rik	13.85	19.87	3.94	0.79	3.31E-028
Odz3	14.70	16.16	8.10	0.00	5.74E-028
Pkia	46.50	60.53	21.13	2.88	3.69E-026
Peg3	24.30	13.56	3.28	2.49	1.08E-025
Ube2d1	25.80	38.85	4.95	6.80	5.79E-025
Sema6c	7.60	10.45	0.40	1.37	7.34E-025
Creld1	17.35	23.80	12.94	0.36	7.77E-025
Cd200	62.12	40.76	20.80	2.43	1.48E-024
Narf	22.44	18.74	3.84	5.85	2.47E-024
Trp53inp2	64.54	66.41	38.66	8.59	8.77E-024
6030419C18Rik	32.26	27.45	6.41	8.49	1.55E-023
Fyn	71.64	63.81	17.62	17.67	1.77E-023
Prkab2	15.90	12.46	2.70	2.83	2.31E-023
Ppfia4	13.33	8.21	3.50	0.09	3.17E-023
Slc9a3r2	15.18	15.48	6.90	2.09	3.67E-023
Ttll1	23.42	24.24	9.21	5.12	4.41E-023
Col23a1	5.36	5.44	0.78	0.01	2.14E-022
Raph1	10.79	9.97	1.70	2.74	2.82E-022
Trim13	11.41	6.30	1.47	0.94	6.20E-022
Gfod1	9.04	9.25	0.24	5.67	8.67E-022
Mef2d	63.05	54.88	13.97	19.20	9.51E-022
Trio	34.18	28.06	6.15	10.88	1.04E-021
Traf4	34.92	28.58	7.61	9.30	1.07E-021
Mapre3	40.15	57.96	18.52	6.92	1.48E-021
Ablim2	4.72	6.88	0.59	0.00	2.63E-021
Klc2	24.47	38.76	6.33	9.31	7.28E-021
Impdh1	47.68	25.87	4.21	16.19	8.13E-021
Slc4a3	17.24	12.34	6.84	1.38	1.17E-020
RP23-26D22.7	6.87	10.90	1.73	1.14	1.56E-020
Samd8	19.32	15.77	7.75	2.83	2.39E-020
Klc1	104.59	127.15	31.64	35.28	3.20E-020
Abr	33.34	48.88	15.34	7.26	3.28E-020
Fndc5	8.38	5.73	1.85	0.00	4.19E-020
BC029214	13.57	13.38	5.77	2.71	7.64E-020
Nav2 (7 56852520..56862516)	16.48	27.04	9.84	3.06	8.75E-020
Pacs2	67.20	48.67	25.48	10.30	3.03E-019
Otud7a	4.80	3.93	0.16	0.01	4.98E-019
AC079443.1	7.95	7.97	1.50	2.21	7.25E-019
Usp13	8.31	4.73	0.84	0.61	7.54E-019
Dennd5b	4.95	5.19	1.05	0.14	1.17E-018
BC024814	6.82	9.08	1.66	1.21	1.71E-018
Shisa2	3.60	3.95	0.23	0.00	4.28E-018
Lass6	15.19	12.56	4.17	3.78	4.40E-018
Clasp1	30.31	27.46	13.83	7.16	4.92E-018
Dip2c	12.82	13.90	4.69	3.55	5.21E-018
Pcdhga11	11.08	9.08	1.57	4.00	6.99E-018
Sfrp2	6.99	3.87	0.09	1.00	7.15E-018
Socs2	6.85	3.83	0.58	0.40	7.82E-018
Nckap5l	12.62	11.94	3.67	3.94	9.42E-018

Palm2	14.55	12.94	7.05	2.46	1.07E-017
Shf	18.17	9.11	2.08	7.12	1.14E-017
Vash2	7.53	9.93	0.46	5.43	2.69E-017
Smarcd3	14.27	21.81	9.13	3.49	3.15E-017
Tesk1	19.63	22.28	9.34	5.94	7.43E-017
Slc7a5	47.95	64.37	10.88	32.85	8.54E-017
Drp2	9.24	5.46	4.11	0.17	1.90E-016
Hip1r	20.71	11.24	4.49	6.27	1.92E-016
Optn	8.04	6.93	4.23	0.48	2.00E-016
Tshz3	5.40	4.76	1.46	0.15	2.45E-016
Rhobtb2	28.18	18.88	11.62	5.35	3.22E-016
6430548M08Rik	10.82	14.56	4.79	2.96	3.40E-016
Ezh1	13.78	8.03	5.16	1.75	4.48E-016
Ptprk	47.85	43.10	10.80	29.86	5.18E-016
Zim1	4.59	2.88	0.08	0.00	7.61E-016
Mef2a	39.95	26.18	18.70	7.07	8.08E-016
Socs7	13.29	20.68	8.92	3.87	1.17E-015
Ndufs5	72.21	82.16	33.76	24.92	1.48E-015
Prune	30.24	24.51	6.70	15.74	2.52E-015
Jund	147.99	117.02	68.54	34.48	2.57E-015
Entpd4	19.68	18.08	7.38	7.05	2.96E-015
Slc38a7	12.89	13.29	9.83	2.86	3.14E-015
Nav2 (7 56219733..56749372)	5.23	8.28	1.93	0.97	4.45E-015
Midn	80.40	68.21	23.61	33.43	4.68E-015
Slc25a29	7.82	5.29	1.67	1.10	6.71E-015
Trak1	24.47	33.57	15.54	7.37	6.88E-015
Wsb2	41.64	46.61	20.88	14.32	7.64E-015
Pcgf3	17.38	17.37	5.51	8.97	9.54E-015
Dcakd	55.37	81.52	26.41	23.39	9.76E-015
Dab2ip	14.39	16.32	9.34	3.64	1.45E-014
Ubr3	23.31	19.54	7.78	8.68	1.47E-014
Hecw2	3.78	3.58	1.03	0.03	2.65E-014
Arhgef3	4.89	4.93	3.46	0.12	3.56E-014
Rpp21	21.34	25.83	12.19	7.22	6.74E-014
Arhgap23	18.94	24.55	8.03	9.01	1.31E-013
Clip1	16.54	16.15	7.14	6.25	1.45E-013
Rcor2	6.17	5.24	0.34	4.02	1.62E-013
Rapgef1	47.48	34.20	21.39	12.17	1.77E-013
Ahdc1	12.03	10.77	6.86	2.72	1.90E-013
Dffa	8.23	9.52	3.71	2.22	3.90E-013
Ccpg1	12.64	13.86	8.48	3.49	7.61E-013
Ubap2	45.21	54.58	20.14	21.47	1.29E-012
Stim2	17.55	10.33	6.56	4.62	1.39E-012
Tmem85	58.81	55.87	18.40	40.04	1.57E-012
Mtap4	103.76	89.53	71.61	28.93	2.57E-012
Golga2	22.89	17.58	8.10	8.94	4.43E-012
Sec14l2	5.22	3.90	2.74	0.03	4.50E-012
Gan	5.91	4.27	1.18	1.10	6.43E-012
Nfu1	31.63	35.78	17.59	12.73	8.16E-012
C330016O10Rik	4.10	3.55	3.07	0.00	1.12E-011
Stk25	53.95	59.64	28.75	22.73	1.34E-011
Tmx4	14.55	10.09	6.98	3.48	1.46E-011
Acvr2a	4.76	7.66	2.50	1.36	4.90E-011
Btbd9	12.41	13.50	9.61	3.87	6.72E-011
Klhl8	7.57	9.05	4.08	2.19	7.03E-011
Fto	16.28	19.93	9.97	6.63	7.12E-011
Ppp2r3a	12.04	8.07	5.19	3.09	8.31E-011
Fbxl2	9.04	5.64	3.19	1.94	9.25E-011
Dcaf6	40.02	27.20	15.39	14.81	9.53E-011
Prickle1	4.73	4.51	0.78	1.79	1.01E-010
Cc2d1a	23.93	26.01	12.35	11.06	1.05E-010
Pcdhb17	6.89	7.57	2.29	2.87	1.38E-010
Larp1b (3 40754550..40781717)	14.91	8.11	4.67	5.54	1.70E-010
Csrnp2	7.33	8.05	1.92	4.32	2.04E-010
Dnajb6	36.66	38.74	16.79	19.54	2.32E-010
Ktn1	38.56	32.82	20.50	13.75	2.35E-010
Sh2b1	31.31	26.40	18.70	10.34	2.55E-010
Nhsl1	4.86	3.90	2.81	0.27	2.77E-010
Tbc1d8	8.08	6.50	5.07	1.47	3.03E-010
Iws1	15.90	20.99	7.36	9.86	3.04E-010
Zmym2	16.13	14.13	10.35	4.95	3.13E-010
B230208H17Rik	23.73	22.74	10.96	11.78	5.46E-010
Freq	26.37	33.74	12.05	16.49	5.53E-010
Prkaca	97.09	68.31	43.39	34.49	5.64E-010
Slco3a1	14.39	9.60	5.40	4.95	6.67E-010
Rps6kb2	12.45	15.05	5.85	6.26	7.36E-010
Hip1	33.82	18.17	15.05	11.94	8.69E-010
1700020C11Rik	2.77	4.43	1.68	0.11	8.95E-010
Zfp346	13.37	7.63	4.53	4.87	1.10E-009
Slain2	23.95	16.22	9.57	9.59	1.33E-009

Chd7	3.85	2.83	2.25	0.00	1.34E-009
Atp6v1d	110.13	102.78	63.59	44.49	1.46E-009
Usp20	13.78	8.47	5.57	4.40	1.48E-009
Eif4e2	24.42	15.66	10.18	9.46	2.39E-009
Ubac1	35.05	18.51	14.57	13.92	2.51E-009
Btbd11	2.66	3.45	0.46	0.58	4.37E-009
Cnot7	6.03	6.60	1.87	3.06	5.24E-009
Fam160b2	15.09	9.19	6.35	5.16	5.56E-009
Map4k4	110.75	123.21	66.83	51.67	6.30E-009
Tecr	310.43	407.93	162.57	190.97	7.42E-009
Atg9a	28.11	23.33	14.03	11.55	8.36E-009
Ncor2	35.42	23.44	19.01	12.31	1.35E-008
AU040320	21.92	16.90	12.12	8.00	1.41E-008
Zfp697	4.45	5.37	1.42	1.65	1.49E-008
Dll1	2.15	2.89	0.55	0.00	2.16E-008
Mex3d	17.44	17.90	7.08	13.45	2.23E-008
Tbc1d25	8.28	6.86	4.00	2.40	2.50E-008
Tmem57	11.10	14.05	5.55	6.23	2.59E-008
Kcmf1	78.18	91.09	38.81	48.25	3.34E-008
Adcy9	5.79	3.36	2.93	0.82	5.16E-008
Dusp4	11.88	7.41	4.63	4.63	7.23E-008
Pcbp4	65.71	86.12	43.02	35.93	9.73E-008
Sarnp (10 128258947..128314664)	67.53	62.44	39.76	31.26	3.11E-007
Gcap14	17.22	16.18	10.64	7.51	3.37E-007
Cd99l2	28.39	30.18	20.77	13.20	3.95E-007
Cdhr1	2.65	2.08	0.71	0.00	4.24E-007
Mtap7d1	127.80	117.69	72.54	61.34	4.91E-007
Ryr3	1.67	1.92	0.22	0.00	5.27E-007
Rtn4	128.68	162.89	88.99	70.19	7.92E-007
Dync1li1	35.01	30.76	17.41	18.41	1.18E-006
Zfp652	7.78	5.56	4.12	2.21	1.38E-006
Sfxn3	37.38	44.84	20.24	25.08	1.47E-006
Slco5a1	1.29	2.07	0.07	0.02	3.01E-006
Prkg1	2.52	3.44	1.30	0.44	3.02E-006
Dis3l2	8.85	10.13	5.25	4.24	3.24E-006
Rabgef1	11.98	8.37	5.38	5.16	3.41E-006
Trak2	18.82	15.18	9.03	9.44	3.46E-006
Ttc19	8.75	8.48	3.37	5.93	4.22E-006
Scamp3	51.91	46.86	29.75	25.90	4.51E-006
Fam13c	11.75	12.80	5.28	9.06	5.35E-006
Armc6	7.62	7.36	2.80	5.19	6.13E-006
Pdhx	10.54	12.67	6.59	5.71	8.70E-006
Mterfd3	6.45	4.98	2.93	2.17	1.00E-005
6030458C11Rik	10.12	7.65	4.13	4.86	1.06E-005
Ric8b	15.74	14.92	7.91	9.21	1.08E-005
Asb1	7.79	4.79	3.52	2.65	1.18E-005
Sgta	93.33	95.70	49.02	63.46	1.28E-005
AC102262.1	2.03	3.15	1.03	0.33	1.78E-005
Nufip2	11.11	11.12	6.80	5.57	1.83E-005
Dvl1	26.58	20.18	12.52	14.63	1.93E-005
Rps6ka2	15.05	16.02	7.36	11.34	2.48E-005
Hectd3	15.24	15.79	10.10	7.86	2.56E-005
Zfp319	7.91	8.69	6.13	3.29	2.87E-005
Ccdc130	9.57	7.92	4.18	4.76	3.09E-005
Rnf20	15.79	14.38	10.59	7.34	5.55E-005
Xpa	9.29	8.35	5.52	4.00	8.14E-005
Acsl1	5.07	5.92	3.07	2.12	9.88E-005
Plcb4	5.05	3.43	2.29	1.39	0.00010479
Rspo1	1.89	1.11	0.27	0.00	0.00011761
Herc1	4.69	5.81	3.38	1.87	0.00020029
Cox19	10.55	9.51	6.20	5.04	0.00020416
Sacs	2.63	3.23	0.82	1.15	0.00025188
Pdhb	84.30	67.36	45.00	46.98	0.00032041
Top1	25.63	19.45	14.26	13.51	0.00042517
6430527G18Rik	42.28	46.43	26.02	28.94	0.00083765
Tmem183a	24.93	25.58	15.79	15.82	0.0010013
Pbx3	4.70	5.10	2.40	2.28	0.0011726
Kcnn1	2.40	2.32	0.71	0.74	0.0012381
Ppp3cc	9.48	9.69	5.71	5.42	0.0016572
Pgap3	3.54	3.83	1.48	1.83	0.0019402
Snapc3	4.50	4.02	1.50	2.86	0.0022168
4932438A13Rik	3.55	4.77	2.30	1.67	0.0031388
Pldn	12.04	11.74	8.07	6.69	0.003697
Col25a1	1.06	1.12	0.00	0.00	0.0051176
RP23-26D22.9	1.46	0.84	0.08	0.00	0.0059188

[a]Data represent RPKM values if not specified otherwise

Supplementary Table S9: Differential expression of *T. gondii* genes of cluster 20 and 21 in SkMCs and neurons versus astrocytes and fibroblasts

		T. gondii in					
**Gene ID**	**Description**	**SkMCs[a]**	**Neurons**	**Astrocytes**	**Fibroblasts**	**Cluster**	**FWER**
TGME49_002170	hypothetical protein	10.91	16.40	0.00	0.00	20	5.93E-35
TGME49_002800	cytochrome c oxidase assembly protein COX11 protein, putative	10.54	11.65	4.58	0.00	20	2.33E-27
TGME49_003850		17.73	27.44	0.00	0.00	20	1.05E-37
TGME49_005370	hypothetical protein	22.15	20.90	9.62	0.00	20	1.69E-33
TGME49_007030	hypothetical protein	11.20	9.90	0.00	0.00	20	3.50E-38
TGME49_007140	SAG-related sequence SRS49B (SAG2X)	13.63	25.71	0.00	0.00	20	3.28E-35
TGME49_009410	*hypothetical protein*	*6.40*	*10.19*	*0.00*	*0.00*	*20*	*1.44E-28*
TGME49_012720	hypothetical protein	10.13	17.02	0.00	0.00	20	9.67E-34
TGME49_013440	hypothetical protein	48.17	50.41	17.44	6.06	20	8.89E-29
TGME49_014200	WD domain, G-beta repeat-containing protein	22.07	18.59	7.06	3.51	20	1.07E-23
TGME49_014760	erythronate-4-phosphate dehydrogenase domain-containing protein	13.69	12.51	3.96	0.00	20	5.11E-31
TGME49_015930	mediator complex subunit MED21 (MED21)	27.34	23.58	5.94	0.00	20	5.37E-36
TGME49_016920	mediator complex subunit MED8 (MED8)	40.20	64.36	6.99	0.00	20	1.06E-32
TGME49_021290	tRNA-Leu	6.61	6.43	0.00	0.00	20	1.39E-31
TGME49_026700	nuclease, putative	14.30	18.66	3.11	0.00	20	7.62E-33
TGME49_027780	hypothetical protein	6.70	5.93	2.91	0.00	20	3.92E-19
TGME49_028020	hypothetical protein	11.78	8.33	0.00	0.00	20	4.22E-33
TGME49_029700	hypothetical protein	22.96	21.66	6.65	0.00	20	5.48E-37
TGME49_031990	hypothetical protein	62.46	74.57	13.57	0.00	20	2.68E-36
TGME49_035000	phosphorylase family protein	9.04	8.79	0.00	0.00	20	1.50E-36
TGME49_038080	hypothetical protein	19.17	24.30	0.00	0.00	20	2.46E-42
TGME49_038090		17.37	23.81	7.54	0.00	20	4.91E-29
TGME49_039090	SAG-related sequence SRS23 (SRS23)	3.02	4.27	0.00	0.00	20	5.54E-17
TGME49_039860	Ras-associated protein Rap1 isoform 1 family protein	5.27	4.20	0.00	0.00	20	2.09E-21
TGME49_040410	protein kinase (incomplete catalytic triad)	10.44	15.70	4.54	0.00	20	2.14E-25
TGME49_040770	cytochrome b5 family heme/steroid binding domain-containing protein	23.16	22.08	6.71	3.88	20	4.83E-28
TGME49_049210	hypothetical protein	11.50	14.85	2.00	3.47	20	1.23E-22
TGME49_050890	3'-5' exonuclease domain-containing protein	9.90	8.76	4.30	0.00	20	1.76E-23
TGME49_050900	hypothetical protein	12.94	12.02	5.62	0.00	20	3.86E-28
TGME49_051660	*hypothetical protein*	*10.08*	*6.69*	*0.00*	*0.00*	*20*	*4.26E-29*
TGME49_055270	hypothetical protein	58.87	53.01	18.60	0.00	20	1.79E-34
TGME49_055710	ULK kinase	9.40	15.80	4.08	0.00	20	5.67E-25
TGME49_056860	WD repeat domain 35 family protein	1.63	1.15	0.00	0.00	20	3.38E-06
TGME49_057130	zinc finger (CCCH type) motif-containing protein	5.10	5.41	0.00	0.00	20	1.79E-25
TGME49_058350	hypothetical protein	12.16	19.17	3.52	3.06	20	1.74E-22
TGME49_058750	hypothetical protein	4.14	2.93	0.00	0.00	20	1.13E-16
TGME49_062440	hypothetical protein	2.95	3.66	0.00	0.00	20	3.95E-17
TGME49_062940	aspartyl proteinase (eimepsin), putative	7.01	6.20	3.04	0.00	20	1.67E-19
TGME49_064180	flagellar associated protein; hypothetical protein	3.48	3.85	0.00	0.00	20	1.58E-19
TGME49_065800		17.02	21.07	0.00	0.00	20	4.49E-43
TGME49_065990	hypothetical protein	22.01	16.22	6.37	0.00	20	7.46E-30
TGME49_067290	hypothetical protein	53.12	68.39	15.38	8.91	20	3.54E-26
TGME49_067640		3.45	6.20	0.00	0.00	20	1.82E-20
TGME49_068250	WD domain, G-beta repeat-containing protein	24.06	24.47	13.06	0.00	20	1.21E-33
TGME49_068360	hypothetical protein	3.74	2.98	0.00	0.00	20	4.01E-17
TGME49_071070	hypothetical protein	28.94	26.62	15.09	0.00	20	2.06E-31
TGME49_071950	hypothetical protein	12.58	21.70	2.73	0.00	20	3.20E-31
TGME49_073990	CobW/P47K family protein	15.69	18.96	2.27	7.89	20	6.01E-22
TGME49_074090	hypothetical protein	20.18	18.74	0.00	0.00	20	8.54E-48
TGME49_077730	hypothetical protein	10.56	8.41	4.59	0.00	20	2.26E-22
TGME49_077970	dolichol-phosphate-mannose synthase family protein	21.45	22.14	12.43	0.00	20	7.65E-33
TGME49_078010		10.40	9.81	3.01	0.00	20	9.17E-29
TGME49_084160	*histone lysine methyltransferase, SET, putative*	*15.82*	*15.74*	*6.87*	*0.00*	*20*	*3.32E-32*
TGME49_085460	hypothetical protein	10.31	10.03	0.00	0.00	20	1.35E-39
TGME49_085740	hypothetical protein	9.40	17.46	0.00	0.00	20	5.53E-33
TGME49_097250	transporter, major facilitator family protein	15.61	20.71	0.00	0.00	20	7.97E-41
TGME49_101220	endonuclease/exonuclease/phosphatase family protein; hypothetical protein; DNA repair protein Rad4 domain-containing protein	21.73	28.18	9.44	5.47	20	6.93E-20
TGME49_104640	hypothetical protein	9.10	16.23	0.00	2.29	20	1.14E-27
TGME49_109860	hypothetical protein	41.38	34.77	0.00	7.81	20	3.75E-36
TGME49_111170	hypothetical protein	5.87	6.23	0.00	0.00	20	7.74E-29
TGME49_112370	RNA pseudouridine synthase superfamily protein	6.67	7.67	0.00	0.00	20	3.82E-30
TGME49_112670	hypothetical protein	7.79	7.92	0.00	0.00	20	1.53E-34
TGME49_114380		29.14	24.74	10.13	0.00	20	7.95E-32
TGME49_114940		11.27	6.98	0.00	0.00	20	1.67E-30
TGME49_115710	hypothetical protein	36.81	36.27	13.71	7.94	20	2.80E-24
TGME49_115960	radical SAM domain-containing protein; hypothetical protein	8.74	10.82	2.53	2.20	20	1.16E-18
TGME49_119700	NEK kinase	11.50	7.12	0.00	0.00	20	7.62E-31
TGME49_119890	hypothetical protein	12.62	17.86	0.00	0.00	20	4.02E-37
TGME49_120030	hypothetical protein	20.34	27.79	7.85	3.41	20	1.31E-23
TGME49_120170	SAG-related sequence SRS16E (SRS6)	4.32	4.20	0.00	0.00	20	1.59E-22
TGME49_120510	hypothetical protein	18.62	16.80	3.24	0.00	20	1.98E-37
TGME49_000240	microneme protein MIC17B (MIC17B)	65.06	56.03	44.63	31.02	21	2.89E-05
TGME49_001790	FHA domain-containing protein	45.47	52.28	37.31	26.70	21	0.00032
TGME49_002020	DnAK-TPR	97.03	73.33	30.65	39.95	21	4.49E-13
TGME49_002460	diacylglycerol kinase accessory domain (presumed) domain-containing protein	22.56	24.61	17.42	7.57	21	2.04E-12
TGME49_002590	hypothetical protein	29.12	33.29	21.68	18.84	21	0.00158
TGME49_003170	OB-fold nucleic acid binding domain-containing protein	57.22	54.32	43.86	33.02	21	0.00428
TGME49_003520	hypothetical protein	10.64	10.71	9.24	3.71	21	1.57E-09

TGME49_004070	YT521-B family protein	67.02	60.71	54.70	33.72	21	0.00016
TGME49_005440	CCT chaperonin gamma subunit	93.55	97.63	70.44	50.21	21	3.14E-05
TGME49_006540	hypothetical protein	92.79	92.70	71.46	57.30	21	0.00834
TGME49_007350	hypothetical protein	33.03	22.20	17.22	9.97	21	7.53E-11
TGME49_008830	hypothetical protein	173.19	224.41	110.72	110.99	21	1.22E-06
TGME49_009690	small nuclear ribonucleoprotein	41.01	47.93	30.54	17.69	21	3.74E-09
TGME49_010410	HMG (high mobility group) box domain-containing protein; hypothetical protein	159.08	138.01	95.43	97.20	21	0.00222
TGME49_013540		25.69	27.26	11.16	9.69	21	7.03E-16
TGME49_014120	hypothetical protein	10.96	11.09	6.35	3.68	21	1.99E-11
TGME49_014890		23.96	28.72	17.73	13.39	21	6.45E-06
TGME49_015920	hypothetical protein	35.61	23.93	12.37	10.75	21	4.83E-14
TGME49_016000	alveolin domain containing intermediate filament IMC3 (ALV3)	151.92	160.14	130.21	92.97	21	0.00806
TGME49_016600	exonuclease III APE (APE)	27.57	34.14	15.97	17.34	21	2.62E-07
TGME49_016660	hypothetical protein	24.07	26.26	11.62	16.15	21	7.10E-08
TGME49_017010	hypothetical protein	20.71	23.36	18.00	13.03	21	0.00669
TGME49_017750	hypothetical protein	49.65	32.20	21.57	12.49	21	1.06E-13
TGME49_018570	Nin one binding (NOB1) Zn-ribbon family protein	48.77	56.07	33.89	22.08	21	8.13E-09
TGME49_019770	30S ribosomal protein S12, putative	32.04	37.65	19.88	17.27	21	9.99E-08
TGME49_020200	hypothetical protein	17.49	20.78	13.03	7.55	21	3.26E-09
TGME49_020220		13.76	10.95	3.99	6.92	21	5.63E-11
TGME49_020260	hypothetical protein	15.42	12.27	4.46	7.76	21	2.22E-11
TGME49_021850	prohibitin family protein, putative	27.60	26.58	2.66	13.89	21	6.28E-27
TGME49_021900	hypothetical protein	48.81	42.02	22.62	24.56	21	2.83E-08
TGME49_022020	phosphoglycerate kinase PGKII (PGKII)	18.50	19.36	10.71	9.31	21	3.10E-08
TGME49_022030	hypothetical protein	7.97	8.81	6.93	3.01	21	5.84E-07
TGME49_022060	hypothetical protein	12.93	14.86	5.62	5.85	21	5.73E-12
TGME49_022300	hypothetical protein	10.37	8.87	4.00	5.22	21	1.05E-07
TGME49_023090		54.03	65.70	33.25	23.79	21	1.28E-10
TGME49_023480	sushi domain (scr repeat) domain-containing protein	5.30	6.43	3.29	1.72	21	7.76E-08
TGME49_024200	tRNA pseudouridine synthase	11.28	12.97	9.80	2.84	21	1.55E-13
TGME49_024490	polyprenyl synthetase superfamily protein	17.18	16.21	9.95	11.53	21	0.00195
TGME49_024890	hypothetical protein	38.54	34.63	25.45	18.62	21	8.72E-06
TGME49_025020	hypothetical protein	19.25	18.11	13.69	10.57	21	0.00068
TGME49_025440	hypothetical protein	7.49	7.95	4.65	1.61	21	2.96E-12
TGME49_026720	translin family protein	48.34	44.73	22.62	28.07	21	8.62E-08
TGME49_026890	hypothetical protein	8.23	5.02	3.58	3.73	21	6.92E-05
TGME49_027320	hypothetical protein	14.84	14.66	2.15	7.47	21	4.79E-22
TGME49_027430	transmembrane amino acid transporter protein	21.27	17.56	15.40	10.70	21	0.00052
TGME49_027640	hypothetical protein	73.91	67.00	53.51	27.89	21	3.85E-09
TGME49_027650	microtubule-associated protein RP/EB family, putative	49.78	54.21	43.25	14.09	21	5.68E-15
TGME49_027870	Tim10/DDP family zinc finger superfamily protein	65.46	86.84	28.44	24.70	21	2.69E-16
TGME49_028250	elongation factor Tu GTP binding domain-containing protein	35.20	43.72	22.61	20.79	21	6.46E-07
TGME49_028650		20.38	15.02	11.31	12.82	21	0.00355
TGME49_030080	DEAD/DEAH box helicase domain-containing protein	12.28	10.26	7.11	6.18	21	0.00023
TGME49_030230	hypothetical protein	39.79	39.49	26.88	20.02	21	1.11E-06
TGME49_030430	vesicle-associated membrane protein, putative	31.78	21.08	18.41	8.00	21	3.02E-12
TGME49_030610	transmembrane protein TMEM222 (TMEM222)	52.21	52.94	33.26	21.02	21	7.84E-11
TGME49_030970	hypothetical protein	52.37	58.66	22.75	26.35	21	2.16E-12
TGME49_031150	hypothetical protein	20.54	15.44	13.38	7.75	21	1.39E-07
TGME49_031370	phospholipase, patatin family protein	11.72	13.47	7.64	7.96	21	0.00169
TGME49_033170	hypothetical protein	53.32	60.72	28.96	20.12	21	2.24E-13
TGME49_033300	RhoGAP domain-containing protein	16.79	19.55	12.15	8.45	21	6.29E-07
TGME49_033350	nuclear transport factor 2, putative	247.25	256.94	145.00	167.96	21	9.96E-05
TGME49_034390	hypothetical protein	35.96	31.80	24.30	12.06	21	1.07E-10
TGME49_034420	ATPase, AAA family protein	31.78	35.27	15.06	8.72	21	1.61E-18
TGME49_035480	pantothenate kinase	21.96	22.82	14.31	8.29	21	1.34E-11
TGME49_035860	subtilisin SUB11 (SUB11)	5.57	6.73	3.22	2.80	21	3.19E-05
TGME49_036800	hypothetical protein	24.33	18.83	13.21	9.18	21	7.27E-09
TGME49_037030		36.80	25.22	15.99	6.94	21	1.50E-16
TGME49_037110	replication factor C subunit 2, putative	25.04	28.79	16.32	4.73	21	1.94E-19
TGME49_037290	hypothetical protein	21.72	22.24	14.52	15.13	21	0.00924
TGME49_038070	glutaredoxin domain-containing protein	44.36	54.53	26.98	26.79	21	4.09E-07
TGME49_038890	hypothetical protein	16.51	20.22	9.93	9.59	21	1.77E-07
TGME49_038940	GDP mannose 4,6-dehydratase, putative	27.67	24.47	12.02	6.96	21	1.08E-17
TGME49_039050	hypothetical protein	25.54	26.16	21.02	12.18	21	8.59E-07
TGME49_040510	hypothetical protein	19.81	15.04	12.91	9.97	21	0.00055
TGME49_041870	tRNA ligase class I (E and Q), catalytic domain-containing protein	10.78	12.92	4.16	5.42	21	8.46E-12
TGME49_042050	DEAD/DEAH box helicase domain-containing protein	7.33	9.22	2.45	3.40	21	1.48E-11
TGME49_043490	BCS1 family isoform 9, putative	22.88	15.52	8.84	7.68	21	3.78E-12
TGME49_044080	hypothetical protein	48.60	48.72	22.12	27.95	21	5.35E-10
TGME49_044140	hypothetical protein	7.52	7.32	4.36	3.78	21	0.00020
TGME49_045520	hypothetical protein	12.10	12.13	7.01	6.09	21	8.01E-07
TGME49_046040	MIF4G domain-containing protein	19.63	22.88	11.04	12.20	21	4.03E-07
TGME49_046100	phosducin, putative	62.33	56.96	13.54	31.36	21	1.04E-18
TGME49_046450	hypothetical protein	26.51	31.94	23.03	10.00	21	9.78E-11
TGME49_046710	hypothetical protein	25.24	26.79	10.96	15.24	21	3.09E-10
TGME49_047460	proliferating cell nuclear antigen PCNA1 (PCNA1)	85.20	95.80	54.63	67.37	21	0.00487
TGME49_047700	AP2 domain transcription factor AP2XII-4 (AP2XII4)	40.26	41.38	25.70	29.15	21	0.00554
TGME49_048530	FATC domain-containing protein	10.17	12.14	7.36	5.12	21	5.94E-06
TGME49_048830	phosphoinositide phospholipase PIPLC (PIPLC)	18.18	19.16	12.50	9.15	21	1.43E-06
TGME49_049620	histone deacetylase HDAC2 (HDAC2)	23.16	26.62	12.07	6.99	21	2.01E-16
TGME49_051550	acyl-coa-binding protein	73.83	49.73	44.41	34.29	21	2.42E-05
TGME49_051810	translation initiation factor eIF-5A, putative	685.09	754.92	374.02	468.18	21	4.54E-06

TGME49_051880	tRNA synthetases class I family protein	81.21	57.46	33.75	21.32	21	3.24E-14
TGME49_052320	Sas10/Utp3/C1D family protein	37.73	45.32	24.58	18.98	21	2.51E-08
TGME49_052480	hypothetical protein	13.30	11.37	7.70	6.69	21	0.00011
TGME49_053680	hypothetical protein	25.84	27.99	8.42	14.63	21	8.54E-15
TGME49_053810	hypothetical protein	19.53	20.61	6.79	9.83	21	4.08E-15
TGME49_054000	hypothetical protein	35.62	30.20	25.48	18.98	21	0.00186
TGME49_054830	hypothetical protein	9.75	8.13	4.84	2.10	21	3.26E-12
TGME49_055920	GINS complex subunit Psf3, putative	26.94	20.25	7.80	6.78	21	1.49E-18
TGME49_056910	hypothetical protein	14.60	10.19	9.87	7.35	21	0.00289
TGME49_056970	vacuolar ATP synthase subunit A, putative	79.88	100.28	61.69	46.89	21	1.50E-05
TGME49_057390	ribosome biogenesis GTP-binding protein YsxC protein	13.92	16.01	10.08	5.25	21	7.33E-10
TGME49_058060	myosin heavy chain, putative	88.43	88.07	50.11	34.82	21	3.25E-12
TGME49_058920	hypothetical protein	35.19	37.09	22.93	8.85	21	4.43E-17
TGME49_058980	hypothetical protein	37.40	50.95	26.00	16.94	21	2.11E-10
TGME49_059140	hypothetical protein	12.69	8.98	3.15	5.47	21	4.57E-12
TGME49_059250	ATP-dependent DNA helicase, RecQ family protein	18.74	22.04	14.65	11.31	21	0.00024
TGME49_060140	phosphomannose isomerase type I protein	38.33	32.09	17.76	15.43	21	1.82E-10
TGME49_060270	HEAT repeat-containing protein	8.44	10.25	3.33	4.64	21	1.27E-09
TGME49_062680	hypothetical protein	13.85	13.32	7.52	7.84	21	1.12E-05
TGME49_062900	hypothetical protein	6.98	5.01	1.35	2.34	21	2.50E-11
TGME49_063110	hypothetical protein	13.30	13.33	7.70	6.69	21	5.09E-07
TGME49_063210	ubiquitin interaction motif domain-containing protein	23.12	30.92	15.06	8.72	21	1.03E-12
TGME49_063340	RNA methyltransferase, TrmH family protein	11.58	9.22	3.35	5.83	21	1.82E-10
TGME49_063470	ubiquitin carboxyl-terminal hydrolase UCHL3 (UCHL3)	46.76	49.89	30.47	17.65	21	1.37E-11
TGME49_063720	HMG (high mobility group) box domain-containing protein	75.52	50.82	39.94	34.70	21	2.72E-06
TGME49_063760	hypothetical protein	9.50	10.22	8.25	2.39	21	1.76E-12
TGME49_064000	aldehyde dehydrogenase	51.14	52.27	19.75	34.31	21	4.60E-11
TGME49_064050	ubiquitin-conjugating enzyme subfamily protein	25.90	22.40	7.50	13.03	21	3.32E-14
TGME49_064080	acyl carrier protein ACP (ACP)	192.73	239.19	112.55	100.16	21	2.44E-09
TGME49_065310	heat shock protein 40, putative	157.91	160.82	124.72	72.23	21	1.51E-07
TGME49_065770	hypothetical protein	19.76	17.32	11.94	3.89	21	4.32E-17
TGME49_066280	HEAT repeat-containing protein	39.43	50.78	27.41	16.67	21	1.29E-10
TGME49_066360	hypothetical protein	18.90	13.37	6.57	5.70	21	8.44E-14
TGME49_066670	hypothetical protein	19.90	13.33	12.35	8.58	21	7.52E-06
TGME49_067560	folate-binding protein YgfZ protein	22.37	20.55	13.46	15.59	21	0.00938
TGME49_068040	hypothetical protein	8.32	8.92	7.23	3.14	21	4.53E-07
TGME49_068810	ck2 beta subunit	91.56	91.46	46.79	65.04	21	3.51E-06
TGME49_069670	hypothetical protein	14.33	16.54	11.80	6.83	21	1.77E-06
TGME49_069760	'chromo' (CHRromatin Organization MOdifier) domain-containing protein	7.99	7.78	4.63	2.68	21	1.35E-08
TGME49_070570	hypothetical protein	12.38	13.14	7.69	5.34	21	2.15E-08
TGME49_071240	hypothetical protein	32.01	32.56	18.54	24.16	21	0.00061
TGME49_071730	serine--tRNA ligase	17.67	18.75	5.12	8.89	21	1.09E-16
TGME49_072270	radical SAM domain-containing protein	31.09	32.04	21.14	16.32	21	3.04E-06
TGME49_072410	phosphogluconate dehydrogenase (decarboxylating), NAD binding domain-containing protein	19.84	15.44	13.79	5.99	21	2.23E-10
TGME49_072520	hypothetical protein	70.17	94.95	46.74	42.37	21	1.80E-07
TGME49_073390	mitochondrial carrier superfamily protein	10.34	7.31	5.99	5.20	21	0.00299
TGME49_073450	sufB/sufD domain-containing protein	13.42	12.11	8.16	4.05	21	5.16E-11
TGME49_073910	hypothetical protein	65.88	56.81	21.46	24.86	21	1.02E-15
TGME49_077520		39.89	42.33	14.85	17.20	21	3.90E-16
TGME49_077850	trypsin domain-containing protein	15.99	13.33	9.92	6.90	21	4.41E-06
TGME49_078060	Mre11 DNA-binding domain-containing protein	11.32	12.01	5.90	6.83	21	3.69E-06
TGME49_078130	hypothetical protein	21.86	20.01	15.64	11.65	21	0.00039
TGME49_078660	P-type ATPase4, putative	36.02	44.70	26.08	18.12	21	8.42E-08
TGME49_080650		11.37	12.79	9.33	6.67	21	0.00368
TGME49_080690	DNA polymerase epsilon subunit B protein	11.81	9.57	6.84	2.97	21	2.79E-11
TGME49_081630	hydroxyacylglutathione hydrolase	49.72	34.16	26.58	28.87	21	0.00038
TGME49_082150	hypothetical protein	15.44	15.30	10.73	9.33	21	0.00364
TGME49_083560		25.14	24.04	18.72	11.75	21	1.51E-06
TGME49_084810		34.30	37.91	18.62	19.41	21	3.87E-08
TGME49_085220	CAP-Gly domain-containing protein	17.41	10.97	3.78	6.57	21	1.11E-14
TGME49_085650	hypothetical protein	8.26	5.60	4.19	4.16	21	0.00246
TGME49_085970	30S ribosomal protein S5, putative	14.54	17.19	9.47	8.23	21	4.30E-06
TGME49_086260	tetratricopeptide repeat-containing protein	36.56	28.97	20.33	13.25	21	2.12E-09
TGME49_087200	hypothetical protein	69.56	45.94	36.26	28.00	21	9.75E-08
TGME49_087260	hypothetical protein	9.74	8.04	5.64	1.63	21	3.61E-13
TGME49_088290	hypothetical protein	24.46	18.12	13.28	9.23	21	1.50E-08
TGME49_088400	LETM1 family protein	25.43	20.06	7.37	9.60	21	2.19E-15
TGME49_088530	NOL1/NOP2/sun family protein	37.08	37.22	24.16	16.79	21	8.34E-09
TGME49_088750	ubiquinol-cytochrome c reductase	216.17	206.21	136.39	147.62	21	0.00856
TGME49_089090	hypothetical protein	42.02	28.80	27.38	15.86	21	1.92E-07
TGME49_089730	Pep3/Vps18/deep orange family protein	34.29	30.20	21.10	17.25	21	1.56E-05
TGME49_089960	hypothetical protein	16.91	20.43	9.79	8.51	21	2.02E-09
TGME49_090000	hypothetical protein	44.91	30.58	27.31	20.34	21	1.48E-05
TGME49_090020	cyclin dependent kinase binding protein	25.67	27.85	16.36	15.50	21	2.46E-05
TGME49_090260	thioredoxin family Trp26 protein	66.45	74.77	44.90	33.44	21	1.93E-07
TGME49_090690		31.66	25.90	20.63	11.95	21	3.05E-08
TGME49_090980	glycine C-acetyltransferase, putative	58.81	53.50	43.79	31.70	21	0.00105
TGME49_092140	NIMA-related protein kinase NIMA1 (NEK)	22.39	18.96	8.34	9.66	21	2.41E-12
TGME49_092710	Toxoplasma gondii family C protein; Toxoplasma gondii family C protein; Toxoplasma gondii family C protein	61.76	77.99	23.85	25.90	21	3.58E-16
TGME49_094280	E3 ubiquitin-protein ligase, putative	39.58	33.84	27.84	19.92	21	0.00024
TGME49_094420	programmed cell death protein 2, c-terminal domain-containing protein	24.26	17.47	12.05	10.46	21	1.26E-07
TGME49_094960	hypothetical protein	15.77	16.97	9.13	11.90	21	0.00079

TGME49_095760	calcium-dependent protein kinase CDPK4A (CDPK4A)	44.54	55.71	24.87	28.81	21	1.43E-07
TGME49_097160	hypothetical protein	19.98	23.64	13.02	7.54	21	7.18E-12
TGME49_097170	50S ribosomal protein L17, putative	36.88	35.42	13.73	23.86	21	5.97E-11
TGME49_097490	hypothetical protein; hypothetical protein	8.37	6.11	3.64	3.16	21	1.63E-06
TGME49_100290	SNARE domain-containing protein	20.38	20.73	11.07	15.38	21	6.93E-05
TGME49_101230		17.77	19.64	8.82	11.50	21	1.24E-07
TGME49_104660	prostaglandin-E synthase	9.92	10.31	8.62	3.74	21	5.47E-08
TGME49_104870	hypothetical protein	98.19	112.38	75.27	43.59	21	1.79E-08
TGME49_105010	pre-mRNA branch site protein p14, putative	71.00	46.24	22.43	29.23	21	5.62E-12
TGME49_105070	hypothetical protein	110.51	80.15	57.61	50.05	21	1.02E-06
TGME49_105770	ABC transporter, ATP-binding domain-containing protein	11.70	11.38	7.62	4.41	21	6.72E-09
TGME49_105890	hypothetical protein	57.37	45.66	33.23	14.43	21	1.50E-13
TGME49_105980	pyruvate dehydrogenase complex subunit PDH-E3I (PDHE3I)	42.75	37.10	25.53	24.20	21	0.00032
TGME49_106560	hypothetical protein	18.86	20.21	9.11	9.49	21	1.03E-10
TGME49_109270		20.02	18.89	11.60	15.11	21	0.00599
TGME49_109790	hypothetical protein	32.94	33.99	9.54	16.58	21	1.09E-17
TGME49_109850	insulin-degrading enzyme	30.10	29.29	10.46	9.09	21	7.42E-21
TGME49_109950	NLE (NUC135) domain-containing protein	25.40	22.72	14.71	12.78	21	2.66E-06
TGME49_110210	hypothetical protein	23.73	17.17	13.12	9.77	21	2.65E-07
TGME49_110320	calreticulin family protein	26.45	21.06	15.32	4.44	21	1.37E-17
TGME49_110450	myosin heavy chain, putative	29.91	23.49	19.75	16.25	21	0.00286
TGME49_110910	WD domain, G-beta repeat-containing protein	10.28	10.46	5.58	5.82	21	1.32E-05
TGME49_112150	hypothetical protein	98.03	101.21	59.10	69.46	21	0.00062
TGME49_112350	IMPACT, putative	21.14	14.65	6.12	5.32	21	1.58E-17
TGME49_112660	hypothetical protein	21.13	23.05	16.32	10.63	21	2.56E-06
TGME49_113340	hypothetical protein	58.54	76.92	39.96	37.87	21	4.33E-06
TGME49_113540	hypothetical protein	44.67	44.86	26.29	30.46	21	0.00019
TGME49_113660	hypothetical protein	25.84	23.13	8.42	14.63	21	2.20E-12
TGME49_113780	hypothetical protein	46.79	36.51	23.91	20.77	21	1.53E-07
TGME49_113820	dynactin p25, putative	49.52	44.25	31.16	12.89	21	1.45E-14
TGME49_113860	regulator of chromosome condensation (RCC1) repeat-containing protein	26.07	25.42	15.29	16.73	21	0.00012
TGME49_114020	hypothetical protein	37.72	25.71	16.38	7.12	21	1.66E-16
TGME49_114230		24.45	22.28	17.00	11.07	21	1.34E-06
TGME49_115190	CAM kinase, SNF1 family	8.78	7.57	3.82	3.32	21	3.29E-08
TGME49_115420	hypothetical protein	28.46	27.86	8.24	14.32	21	2.91E-17
TGME49_115530	hypothetical protein	28.12	29.22	15.88	16.98	21	1.70E-06
TGME49_115590	macro domain-containing protein	22.26	18.60	10.75	3.73	21	1.86E-19
TGME49_115820	hypothetical protein	25.64	34.01	14.85	12.90	21	1.63E-10
TGME49_116720		34.62	27.31	19.25	10.45	21	1.43E-11
TGME49_118210	hypothetical protein	17.02	16.31	12.32	6.42	21	3.24E-09
TGME49_118330	histone lysine acetyltransferase MYST-A (MYSTA)	14.93	17.69	6.49	6.76	21	7.66E-13
TGME49_118430	malate dehydrogenase MDH (MDH)	126.92	171.53	71.58	63.86	21	5.42E-11
TGME49_118710	ATP-binding cassette sub-family F member 1	25.02	28.03	18.11	12.59	21	7.13E-07
TGME49_120720	hypothetical protein	70.98	63.56	53.96	33.48	21	1.86E-05
TGME49_121410	hypothetical protein	28.44	29.54	19.13	19.39	21	0.00519

[a]Data represent RPKM values if not specified otherwise

Supplementary Table S10: Differential expression of *T. gondii* genes of cluster 9 and 10 in SkMCs and neurons versus astrocytes and fibroblasts

		T. gondii in					
Gene ID	Description	SkMCs[a]	Neurons	Astrocytes	Fibroblasts	Cluster	FWER
TGME49_000470	hypothetical protein	39.77	29.22	50.50	55.42	9	0.00033
TGME49_001220	zinc finger protein	17.05	9.91	23.28	22.06	9	6.00E-08
TGME49_001520	protein phosphatase 2C domain-containing protein	43.15	19.80	70.30	89.57	9	6.86E-16
TGME49_002150		18.15	11.95	26.28	21.31	9	2.02E-05
TGME49_003060	hypothetical protein	207.71	81.18	378.00	360.16	9	3.05E-19
TGME49_003530		103.15	56.04	134.42	122.34	9	1.43E-07
TGME49_003740	*hypothetical protein*	*99.57*	*39.80*	*117.65*	*144.29*	*9*	*3.17E-12*
TGME49_005220	U5 snRNP-associated subunit, putative	12.08	10.53	25.49	28.66	9	1.03E-13
TGME49_005260	transporter, cation channel family protein	7.54	5.78	15.29	11.38	9	3.80E-08
TGME49_005510	nucleolar protein 5, putative	42.24	42.19	88.50	90.01	9	1.10E-11
TGME49_005520	hypothetical protein	9.70	1.86	18.26	12.20	9	2.02E-18
TGME49_006520	hypothetical protein	7.88	4.18	13.69	11.89	9	1.16E-10
TGME49_008530	nicotinate phosphoribosyltransferase	40.38	31.89	57.64	47.90	9	0.00571
TGME49_009550	hypothetical protein	7.07	6.56	16.88	16.00	9	2.24E-13
TGME49_010220	hypothetical protein	25.44	21.75	44.21	38.41	9	2.88E-06
TGME49_010230	hypothetical protein	36.49	21.71	63.41	55.09	9	4.66E-11
TGME49_011250	hypothetical protein	46.28	19.10	93.81	93.14	9	4.07E-22
TGME49_012200	hypothetical protein	64.22	14.20	104.62	72.71	9	8.53E-18
TGME49_012900	hypothetical protein	48.91	43.59	84.99	79.52	9	7.68E-07
TGME49_013030	hypothetical protein	95.17	55.13	124.03	111.34	9	2.17E-06
TGME49_013050	hypothetical protein	131.87	85.60	185.91	214.10	9	1.88E-08
TGME49_013370	formin FRM3 (FRM3)	10.15	5.18	11.41	14.42	9	3.13E-07
TGME49_014810	hypothetical protein	15.81	17.24	27.47	31.82	9	1.12E-06
TGME49_015390	TIM10 family protein, putative	50.62	56.29	81.68	87.34	9	0.00044
TGME49_015740	notchless, putative	12.89	14.82	22.40	24.33	9	1.49E-05
TGME49_015970	hypothetical protein	31.32	2.77	63.50	47.28	9	3.75E-24
TGME49_016020	peptidase family c78 protein	29.89	20.27	48.48	45.13	9	6.06E-09
TGME49_016310	hypothetical protein	3.38	2.39	8.80	10.20	9	1.55E-15
TGME49_016670	FUSE-binding protein 2 / KH-type splicing regulatory protein (FUBP2)	84.45	61.41	109.47	105.22	9	0.00123
TGME49_016810	5'-nucleotidase, C-terminal domain-containing protein	59.16	39.10	118.50	86.82	9	1.22E-10
TGME49_017440	HCNGP family protein	34.22	22.19	41.62	48.21	9	1.23E-05
TGME49_017530	hypothetical protein	32.98	8.88	45.58	47.52	9	8.70E-20
TGME49_017540	RNA recognition motif-containing protein	86.14	59.74	108.11	127.98	9	1.77E-05
TGME49_017560	DNA-directed RNA polymerase II RPB10 (POLR2L)	122.09	64.30	135.01	150.80	9	4.65E-07
TGME49_017850	Sucrose-6F-phosphate phosphohydrolase	13.86	10.54	28.90	29.29	9	2.65E-15
TGME49_018420	WD domain, G-beta repeat-containing protein	56.24	56.88	82.86	103.36	9	8.27E-05
TGME49_018600	RNA recognition motif-containing protein	60.34	29.35	90.29	91.09	9	9.21E-14
TGME49_018740	membrane protein, putative	126.07	57.78	179.23	190.31	9	2.26E-13
TGME49_019810	hypothetical protein	110.52	54.50	128.03	136.50	9	9.78E-09
TGME49_020390	hypothetical protein	105.14	74.39	142.73	119.03	9	0.00151
TGME49_020860	DEAD/DEAH box helicase	19.22	20.40	37.58	43.52	9	1.07E-09
TGME49_020920	hypothetical protein	5.08	4.40	7.94	7.66	9	0.00706
TGME49_020950	hypothetical protein	126.59	61.37	171.08	205.25	9	8.99E-12
TGME49_021460	phosphoglycerate mutase family protein	39.79	21.70	48.97	50.05	9	4.78E-08
TGME49_021690	hypothetical protein	8.44	6.40	15.72	16.39	9	3.65E-11
TGME49_022900	phosphoserine phosphatase	45.02	26.28	54.77	67.97	9	1.02E-07
TGME49_023790	hypothetical protein	38.04	19.28	47.03	53.00	9	2.11E-09
TGME49_023910	acyltransferase domain-containing protein	47.14	16.89	61.44	56.93	9	3.20E-14
TGME49_024140	hypothetical protein	7.59	6.71	13.18	11.45	9	9.35E-05
TGME49_024230	AP2 domain transcription factor AP2X-3 (AP2X3)	3.26	2.52	7.78	8.60	9	4.66E-12
TGME49_024700	hypothetical protein	16.35	13.73	28.41	24.68	9	1.73E-06
TGME49_025430	Ras-related GTP binding A family protein	8.96	5.02	18.16	22.54	9	7.20E-18
TGME49_025550	*phosphatidylserine decarboxylase*	*59.98*	*36.51*	*95.03*	*90.55*	*9*	*1.74E-10*
TGME49_025940	hypothetical protein	61.60	61.44	92.17	98.15	9	0.00162
TGME49_025960	STE kinase	10.75	6.09	13.08	14.28	9	3.35E-06
TGME49_026010	pterin-4-alpha-carbinolamine dehydratase	27.90	15.63	48.48	42.12	9	7.55E-12
TGME49_026060	transmembrane amino acid transporter protein	31.83	28.96	48.40	51.49	9	5.93E-05
TGME49_026320	hypothetical protein	17.04	11.84	23.27	22.05	9	3.88E-05
TGME49_026540	protein kinase	79.18	39.41	113.99	99.03	9	6.31E-10
TGME49_026670	hypothetical protein	27.51	11.25	50.78	36.33	9	3.68E-14
TGME49_027100	hypothetical protein	8.31	8.82	21.66	25.09	9	2.44E-16
TGME49_027580	transmembrane amino acid transporter protein	178.32	39.06	196.14	244.48	9	3.99E-18
TGME49_029220	hypothetical protein	97.16	56.64	118.87	131.70	9	4.98E-07
TGME49_029280	hypothetical protein	33.56	24.38	41.65	47.04	9	0.00035
TGME49_029430	hypothetical protein	30.45	14.81	39.68	41.36	9	6.30E-11
TGME49_029470		32.83	29.61	51.34	46.25	9	0.00094
TGME49_029720	hypothetical protein	7.28	6.09	13.22	12.99	9	3.06E-08
TGME49_029750	hypothetical protein	17.46	18.97	31.41	26.35	9	0.00050
TGME49_030000	hypothetical protein	4.09	1.71	5.59	5.30	9	4.24E-06
TGME49_031010	general transcription factor IIE polypeptide 1 GTF2E1 (GTF2E1)	19.83	19.07	25.84	33.67	9	0.00134
TGME49_031030	hypothetical protein	9.67	9.69	21.00	21.89	9	1.40E-12
TGME49_031180	hypothetical protein	29.51	14.86	59.17	58.26	9	2.48E-19
TGME49_031220	hypothetical protein	33.89	17.32	55.61	45.47	9	4.47E-11
TGME49_032080	hypothetical protein	6.82	4.63	9.45	8.83	9	0.00135
TGME49_032400	PAN domain-containing protein	16.77	5.56	21.85	25.31	9	8.62E-16
TGME49_032560	*hypothetical protein*	*9.65*	*8.71*	*16.77*	*20.40*	*9*	*3.58E-09*
TGME49_032810	Erv1 / Alr family protein	75.79	35.28	79.71	102.36	9	5.89E-09
TGME49_032960	oxidoreductase, 2OG-Fe(II) oxygenase family protein	110.01	33.21	131.83	137.43	9	7.52E-17
TGME49_034180	hypothetical protein	96.34	64.12	162.99	149.25	9	7.27E-10
TGME49_034360	DNA topoisomerase I, putative	23.79	23.98	34.10	43.09	9	0.00018

TGME49_034580	ImpB/MucB/SamB family protein	1.56	1.65	4.06	4.71	9	1.05E-06
TGME49_035140	hypothetical protein	155.99	66.29	233.04	249.83	9	2.81E-15
TGME49_035560	hypothetical protein	14.34	13.11	33.23	28.87	9	4.14E-12
TGME49_036290	hypothetical protein	9.96	3.96	12.98	15.04	9	6.16E-12
TGME49_036930	hypothetical protein	5.60	4.62	8.97	9.35	9	7.23E-05
TGME49_037130	cytochrome b, putative	237.48	26.73	412.65	407.37	9	6.04E-29
TGME49_037260	Coiled-coil domain containing 124 family protein	35.65	36.08	51.62	65.77	9	9.41E-05
TGME49_037410	protein phosphatase 2C domain-containing protein	14.24	9.45	18.56	21.50	9	1.29E-06
TGME49_037450	hypothetical protein	9.96	6.87	22.50	21.05	9	1.13E-16
TGME49_037510	hypothetical protein	6.31	4.62	13.31	12.25	9	1.03E-11
TGME49_039260	histone H4	160.11	33.04	332.31	322.26	9	2.38E-27
TGME49_039540	LEM3 (ligand-effect modulator 3) family / CDC50 family protein	28.57	23.82	56.73	55.45	9	3.97E-12
TGME49_039720	50S ribosomal protein l24, putative	24.50	21.36	51.69	47.54	9	1.35E-11
TGME49_039780	hypothetical protein	27.06	27.76	54.07	61.27	9	1.83E-10
TGME49_039900	cyclin-dependent kinase	21.77	15.63	35.60	30.93	9	3.11E-07
TGME49_040780	hypothetical protein	27.41	28.74	44.22	47.28	9	0.00011
TGME49_042080	hypothetical protein	43.22	38.22	69.73	55.92	9	0.00110
TGME49_043330	hypothetical protein	2.92	2.00	5.70	4.40	9	0.00013
TGME49_043970	hypothetical protein	8.62	9.44	17.80	14.65	9	3.93E-06
TGME49_044060	ankyrin repeat-containing protein	33.18	24.17	54.26	44.19	9	2.29E-06
TGME49_044350	DnaJ domain-containing protein	27.27	14.47	33.17	41.17	9	5.30E-09
TGME49_044410	cysteine dioxygenase type i protein; hypothetical protein; hypothetical protein	39.62	26.59	50.63	54.54	9	1.56E-05
TGME49_044430	pseudouridylate synthase, putative	11.86	6.82	20.61	17.90	9	2.06E-11
TGME49_044550	hypothetical protein	24.24	18.82	35.09	28.46	9	0.00264
TGME49_044640	hypothetical protein	31.68	17.46	63.52	55.18	9	5.43E-15
TGME49_045770	hypothetical protein	296.24	228.61	383.54	371.20	9	0.00759
TGME49_046030	mediator complex subunit MED17 (MED17)	13.27	9.39	19.76	22.89	9	1.72E-08
TGME49_046460	hypothetical protein	13.40	8.30	27.94	28.32	9	4.80E-18
TGME49_046960	hypothetical protein	24.13	17.46	28.58	36.42	9	9.85E-05
TGME49_047450	hypothetical protein	25.37	26.70	35.26	43.08	9	0.00476
TGME49_047930	SNARE domain-containing protein	15.76	9.76	36.51	31.72	9	1.09E-16
TGME49_048650		35.94	37.08	52.04	68.72	9	4.27E-05
TGME49_049020	kinesin motor domain-containing protein	5.92	6.18	10.29	12.51	9	3.21E-06
TGME49_049190	AP2 domain transcription factor AP2XII-6 (AP2XII6)	41.02	21.53	45.14	57.80	9	7.06E-08
TGME49_049350	esterase/lipase/thioesterase domain-containing protein	41.67	33.47	64.86	60.28	9	1.48E-05
TGME49_049480	tetratricopeptide repeat-containing protein	20.51	7.51	22.91	30.95	9	6.21E-13
TGME49_049670	cathepsin B	108.97	45.67	145.44	133.50	9	4.36E-12
TGME49_049710	Parkinson disease 7 domain containing 1 family protein	3.42	2.87	8.91	7.74	9	9.94E-11
TGME49_050030	hypothetical protein	12.42	3.30	16.19	18.75	9	2.69E-17
TGME49_050100	hypothetical protein	35.03	17.30	58.33	61.69	9	1.47E-15
TGME49_050750	tRNA (uracil-5-)-methyltransferase	58.86	21.79	72.55	72.32	9	1.33E-14
TGME49_051170	KRUF family protein	158.36	104.96	191.36	195.09	9	0.00020
TGME49_051440	troponin c, isotype gamma, putative	32.96	25.27	54.10	60.82	9	2.12E-09
TGME49_051950	ribosomal protein l7/l12 c-terminal domain-containing protein	26.59	28.69	50.83	48.17	9	4.91E-07
TGME49_053080	hypothetical protein	27.04	21.52	36.91	40.81	9	0.00012
TGME49_053400	hypothetical protein	14.10	17.32	31.31	37.84	9	2.42E-11
TGME49_054460	hypothetical protein	54.41	28.47	74.85	68.45	9	3.82E-09
TGME49_056030	hypothetical protein	48.12	24.47	58.53	65.38	9	4.67E-09
TGME49_056330		75.21	28.08	101.64	88.30	9	5.03E-13
TGME49_058040	hypothetical protein	13.78	3.66	17.96	20.80	9	4.51E-18
TGME49_058560	hypothetical protein	15.12	9.69	32.83	28.52	9	6.55E-15
TGME49_058670	hypothetical protein	15.88	11.85	21.55	22.47	9	5.30E-05
TGME49_059580	hypothetical protein	28.21	31.19	49.02	53.23	9	5.88E-06
TGME49_059660	orotate phosphoribosyltransferase	61.15	37.86	72.10	72.52	9	4.14E-05
TGME49_059840	hypothetical protein	10.58	10.11	16.55	17.57	9	0.00016
TGME49_059990	SAC3/GANP family protein	29.64	34.85	53.03	55.28	9	1.00E-05
TGME49_061230	ankyrin repeat-containing protein	115.90	78.04	158.67	128.57	9	0.00034
TGME49_061250	histone H2A1	90.67	23.05	167.39	188.19	9	2.51E-23
TGME49_061440	ARM repeats containing protein	139.79	102.93	197.72	171.77	9	0.00055
TGME49_061580	histone H2AX	62.57	5.53	130.47	113.34	9	1.83E-27
TGME49_061750	rhoptry neck protein RON10 (RON10)	38.48	23.14	50.14	49.37	9	3.59E-07
TGME49_062420	AP2 domain transcription factor APVIIb-1/ADA2-B (APVIIB1)	15.14	14.78	26.85	23.81	9	2.24E-05
TGME49_062490	alpha/beta hydrolase, putative	125.60	118.31	167.49	198.42	9	0.00645
TGME49_062600	hypothetical protein	7.65	3.16	13.29	15.39	9	4.73E-16
TGME49_063100	hypothetical protein	23.76	17.60	58.06	69.50	9	8.77E-19
TGME49_063220	rhoptry kinase family protein ROP21 (ROP21)	50.50	34.39	61.43	80.05	9	3.02E-06
TGME49_063360	WD domain, G-beta repeat-containing protein	8.93	8.84	18.62	16.18	9	3.55E-08
TGME49_063400	hypothetical protein	31.63	30.22	93.45	95.51	9	4.53E-21
TGME49_063840	hypothetical protein	41.66	29.65	53.42	53.90	9	0.00022
TGME49_064740	hypothetical protein	49.08	28.27	61.08	58.07	9	1.83E-06
TGME49_066920	3'5'-cyclic nucleotide phosphodiesterase domain-containing protein	281.20	147.85	332.38	340.58	9	5.38E-08
TGME49_067650	hypothetical protein	8.28	7.68	14.38	16.66	9	1.15E-07
TGME49_067670	hypothetical protein	29.90	7.78	39.73	37.17	9	1.04E-18
TGME49_067770	hypothetical protein	6.34	5.05	16.52	14.35	9	6.27E-15
TGME49_067820	electron transfer flavoprotein subunit beta, putative	12.33	8.73	16.07	18.62	9	2.28E-05
TGME49_068170	hypothetical protein	113.11	45.85	150.14	161.26	9	7.56E-14
TGME49_068910	signal peptidase I protein	18.81	13.98	22.88	28.40	9	0.00016
TGME49_069900	WD domain, G-beta repeat-containing protein	63.41	49.01	76.65	87.40	9	0.00573
TGME49_070210	kinesin motor domain-containing protein	7.83	7.85	11.34	13.79	9	0.00201
TGME49_070330	cell-cycle-associated protein kinase, putative	33.26	22.06	50.57	46.03	9	1.86E-07
TGME49_070940	hypothetical protein	10.44	9.70	18.14	15.76	9	7.22E-05
TGME49_071440	NPL4 family protein	25.07	28.55	43.57	47.31	9	1.19E-05
TGME49_071450	hypothetical protein	28.96	21.34	58.70	58.28	9	7.40E-15

TGME49_072460	hypothetical protein	47.64	32.30	60.37	80.91	9	2.16E-07
TGME49_072710	AP2 domain transcription factor AP2VIII-4 (AP2VIII4)	23.41	14.76	25.96	31.58	9	5.95E-05
TGME49_072880		13.84	13.46	24.04	20.89	9	9.95E-05
TGME49_073030	phosphoglycerate mutase family protein	44.21	49.85	96.02	83.41	9	3.80E-08
TGME49_075990	hypothetical protein	18.83	16.53	32.72	26.24	9	3.38E-05
TGME49_076120	histone lysine methyltransferase, SET, putative	5.55	3.76	6.43	8.38	9	0.00373
TGME49_076890	hypothetical protein	24.00	27.10	41.71	44.76	9	1.20E-05
TGME49_077090	carrier superfamily protein	56.89	29.07	87.87	83.49	9	1.85E-12
TGME49_077570	GPI transamidase subunit PIG-U protein; Toxoplasma gondii family D protein	6.25	8.71	13.58	16.51	9	9.95E-09
TGME49_077760	adenylosuccinate lyase, putative	31.41	36.54	50.38	65.66	9	7.96E-06
TGME49_078070	Toxoplasma gondii family A protein	6.26	4.43	8.16	9.45	9	0.00111
TGME49_078600	hypothetical protein	36.36	36.17	61.20	51.45	9	0.00148
TGME49_078790	Tctex2-related light chain	11.01	11.29	15.30	19.94	9	0.00047
TGME49_078850	DHHC zinc finger domain-containing protein	42.31	25.78	61.27	49.68	9	1.76E-06
TGME49_081390	phosphofructokinase domain-containing protein	8.38	6.57	11.45	12.66	9	0.00075
TGME49_081780	protein kinase, putative	10.01	7.08	26.09	30.23	9	1.27E-20
TGME49_082170	hypothetical protein	48.23	27.12	59.86	67.61	9	6.28E-08
TGME49_083730	endomembrane protein 70 subfamily protein	18.33	20.67	31.86	38.05	9	1.16E-06
TGME49_085150	hypothetical protein	34.88	20.67	60.60	47.38	9	8.63E-10
TGME49_085670	hypothetical protein	19.56	4.04	22.66	29.53	9	1.20E-18
TGME49_086000	hypothetical protein	16.48	11.34	22.28	19.35	9	0.00040
TGME49_086010	hypothetical protein	29.59	34.50	79.46	69.03	9	9.68E-13
TGME49_087040	hypothetical protein	31.69	4.08	60.08	56.54	9	1.32E-27
TGME49_087240	hypothetical protein	20.85	6.85	41.41	31.48	9	9.30E-18
TGME49_087520	hypothetical protein	20.26	15.23	26.40	30.59	9	6.80E-05
TGME49_088370	hypothetical protein	34.83	29.16	60.52	49.07	9	7.94E-06
TGME49_089510	hypothetical protein	16.22	11.48	18.12	24.49	9	7.77E-05
TGME49_089630	microneme protein MIC16 (MIC16)	136.34	75.75	164.01	176.79	9	2.16E-07
TGME49_089980	HAD hydrolase, family IIA protein	17.19	6.34	22.40	25.95	9	1.46E-14
TGME49_090190	hypothetical protein	32.73	22.61	42.65	52.49	9	1.19E-06
TGME49_090650	hypothetical protein	14.86	10.95	21.52	18.70	9	0.00020
TGME49_090870	patched family protein	46.88	26.60	57.70	66.83	9	8.74E-08
TGME49_091120	trafficking protein mon1 subfamily protein	12.96	8.02	18.76	16.30	9	1.70E-06
TGME49_092050	calcium dependent protein kinase CDPK8 (CDPK8)	16.67	9.66	33.79	33.55	9	4.26E-18
TGME49_092340	zinc finger, C3HC4 type (RING finger) domain-containing protein	19.35	11.73	30.01	27.12	9	4.52E-09
TGME49_092810	Toxoplasma gondii family C protein; Toxoplasma gondii family C protein; Toxoplasma gondii family C protein	35.41	22.49	67.13	82.61	9	2.81E-15
TGME49_094310	hypothetical protein	16.69	6.64	28.99	25.19	9	5.36E-16
TGME49_094690	rhomboid protease ROM5 (ROM5)	58.79	24.49	71.24	70.07	9	3.76E-12
TGME49_095020	Sterol-sensing domain of SREBP cleavage-activation domain-containing protein	12.96	13.55	18.43	21.35	9	0.00730
TGME49_095050	tRNA ligase class II core domain (G, H, P, S and T) domain-containing protein	11.43	12.43	22.84	18.98	9	4.06E-06
TGME49_095410	transcription initiation factor TFIID complex subunit TAF6 (TAF6)	19.00	13.75	27.01	26.07	9	1.14E-05
TGME49_095440	hypothetical protein	29.83	7.51	63.80	51.96	9	8.52E-22
TGME49_097850	Branched-chain-amino-acid aminotransferase	31.35	26.88	48.19	43.69	9	0.00044
TGME49_097930		12.80	3.96	16.68	19.32	9	5.72E-16
TGME49_097970	aspartyl aminopeptidase	16.78	21.08	46.66	50.67	9	5.84E-16
TGME49_100300	hypothetical protein	17.52	7.75	26.63	26.44	9	2.40E-15
TGME49_104900	hypothetical protein	31.30	26.86	51.68	51.98	9	1.83E-07
TGME49_105620	hypothetical protein	12.11	9.82	14.03	18.29	9	0.00467
TGME49_106270	hypothetical protein	35.07	7.09	39.18	45.38	9	1.19E-19
TGME49_106360		41.76	13.85	54.43	57.79	9	2.38E-16
TGME49_106440	hypothetical protein	36.29	18.24	48.13	49.02	9	8.72E-11
TGME49_106910	hypothetical protein	25.47	18.58	41.48	33.64	9	3.12E-06
TGME49_108960	ATPase, AAA family protein	2.05	1.09	3.56	3.09	9	0.00088
TGME49_109140	transducin beta-like protein TBL1 (TBL1)	48.15	37.62	64.49	60.57	9	0.00598
TGME49_109370	adaptin n terminal region domain-containing protein	7.53	5.33	16.36	13.26	9	2.49E-11
TGME49_109410	AP2 domain transcription factor AP2XI-1 (AP2XI1)	15.41	13.04	23.91	23.26	9	1.59E-05
TGME49_109930	melibiase subfamily protein	24.85	20.74	29.69	37.52	9	0.00439
TGME49_110200	hypothetical protein	8.66	3.06	17.55	17.43	9	4.61E-22
TGME49_110540		7.05	3.43	15.31	10.64	9	4.56E-13
TGME49_110840	hypothetical protein	5.88	5.72	10.22	8.88	9	0.00330
TGME49_111490	hypothetical protein	27.64	18.53	45.50	39.53	9	2.62E-08
TGME49_111760	hypothetical protein	16.61	14.02	24.93	20.52	9	0.00515
TGME49_111840	hypothetical protein	118.05	45.24	188.04	193.06	9	4.29E-18
TGME49_112040		27.82	20.30	33.24	36.75	9	0.00301
TGME49_112200	serine/threonine protein phosphatase	51.16	19.66	64.96	62.37	9	1.47E-13
TGME49_112570	CMGC kinase, MAPK family (ERK) MAPK-1	35.40	13.15	51.26	44.53	9	5.56E-14
TGME49_112710		6.73	6.70	11.70	12.71	9	7.94E-06
TGME49_113160	hypothetical protein	32.65	23.10	39.71	49.28	9	3.72E-05
TGME49_113360	hypothetical protein	10.31	7.39	15.92	15.56	9	5.08E-07
TGME49_113750	hypothetical protein	10.51	5.57	18.26	15.86	9	8.48E-12
TGME49_114000	peptide methionine sulfoxide reductase msrB, putative	58.16	37.84	68.58	68.98	9	0.00029
TGME49_114070	hypothetical protein	51.20	32.52	56.61	63.23	9	0.00043
TGME49_114450	hypothetical protein	225.56	165.93	289.50	278.58	9	0.00270
TGME49_114490	hypothetical protein	120.49	21.31	222.45	181.89	9	3.01E-22
TGME49_114850	hypothetical protein	15.44	15.02	26.83	23.31	9	9.95E-05
TGME49_115170	O-phosphoseryl-tRNA(Sec) selenium transferase	33.68	9.39	38.26	46.92	9	4.99E-16
TGME49_115780	myosin regulatory light chain, putative	60.52	35.03	95.60	74.75	9	2.23E-08
TGME49_116510	hypothetical protein	22.63	18.95	51.12	41.00	9	2.57E-11
TGME49_118400	hypothetical protein	34.63	18.99	53.16	43.56	9	5.30E-09
TGME49_118440	helicase associated domain (ha2) protein	70.06	52.93	105.19	109.20	9	4.35E-07

TGME49_118510	N-ethylmaleimide-sensitive fusion protein, putative	26.90	25.12	40.26	40.61	9	0.00066
TGME49_118550	hypothetical protein	40.36	16.06	74.52	60.93	9	5.97E-16
TGME49_118700	eukaryotic initiation factor-6, putative	38.53	29.54	50.22	58.17	9	0.00012
TGME49_119500	hypothetical protein	20.81	13.80	34.87	26.93	9	1.29E-07
TGME49_119570	WD domain, G-beta repeat-containing protein	46.96	18.14	60.12	70.88	9	1.03E-13
TGME49_120190	SAG-related sequence SRS16B (SRS9)	6.68	3.35	9.67	10.08	9	4.14E-09
TGME49_003800	hypothetical protein	0.00	1.14	6.69	3.88	10	3.84E-18
TGME49_004110	eIF2 kinase IF2K-C	0.00	33.61	49.84	43.30	10	1.99E-29
TGME49_006570	kinesin, putative	0.00	1.39	2.27	1.97	10	7.43E-06
TGME49_007190		4.65	9.87	20.20	14.04	10	6.69E-14
TGME49_010590	KRUF family protein	2.97	3.68	23.22	17.93	10	1.24E-28
TGME49_011000	hypothetical protein	3.17	6.45	19.27	9.57	10	1.83E-17
TGME49_011610	hypothetical protein	12.75	15.78	38.77	33.68	10	6.22E-15
TGME49_013620	ABC1 family protein	2.66	4.55	9.25	5.36	10	8.61E-08
TGME49_015340	AP2 domain transcription factor AP2X-10 (AP2X10)	2.88	2.30	22.56	17.42	10	5.04E-31
TGME49_016330	hypothetical protein	0.00	2.05	4.47	3.88	10	3.87E-13
TGME49_016340	hypothetical protein	0.00	4.97	10.27	4.46	10	1.36E-18
TGME49_016840	hypothetical protein	2.02	2.51	7.03	6.11	10	9.78E-11
TGME49_017630	hypothetical protein	2.41	1.28	8.37	7.27	10	2.48E-17
TGME49_018290	WD domain, G-beta repeat-containing protein	1.20	0.74	5.20	3.62	10	4.41E-11
TGME49_018320		3.50	5.27	21.30	15.86	10	3.85E-23
TGME49_019450	WD domain, G-beta repeat-containing protein	0.00	0.51	3.35	1.94	10	5.05E-10
TGME49_023550	hypothetical protein	7.74	11.63	60.49	46.71	10	2.51E-27
TGME49_023870	hypothetical protein	0.00	4.56	8.53	7.41	10	2.82E-20
TGME49_025400	hypothetical protein	0.00	1.98	4.33	3.76	10	7.26E-13
TGME49_025410	histone H3 centromeric CENH3	15.69	13.18	47.70	35.52	10	2.26E-16
TGME49_028700		16.52	0.73	114.85	87.30	10	1.12E-34
TGME49_031060	subtilisin SUB9 (SUB9)	0.00	1.00	5.89	3.41	10	4.74E-16
TGME49_031380	DNA-directed RNA polymerase II RPB4 (POLR2D)	27.45	10.52	79.50	69.06	10	4.36E-25
TGME49_031400	tubulin/FtsZ family, GTPase domain-containing protein	3.47	3.69	18.11	10.49	10	1.28E-19
TGME49_033920	zinc finger, C3HC4 type (RING finger) domain-containing protein	0.00	3.51	20.68	5.99	10	1.46E-28
TGME49_035890	dual-specificity protein phosphatase	0.00	7.53	44.36	25.69	10	8.11E-30
TGME49_042270	hypothetical protein	6.69	21.60	37.79	25.25	10	1.39E-15
TGME49_043380	hypothetical protein; hypothetical protein	7.88	4.18	25.11	15.87	10	3.16E-19
TGME49_046590	hypothetical protein	0.00	16.81	90.07	52.17	10	2.54E-29
TGME49_052510	hypothetical protein	25.54	40.86	80.69	59.58	10	4.68E-11
TGME49_054140	hypothetical protein; DNA-directed RNA polymerase II RPABC4 (POLR2K)	0.00	10.03	42.25	24.47	10	5.61E-28
TGME49_055360	hypothetical protein	0.00	4.46	27.38	9.52	10	1.50E-28
TGME49_057100	hypothetical protein	3.01	9.32	23.55	27.28	10	3.85E-26
TGME49_058620	hypothetical protein; hypothetical protein	0.00	2.07	4.51	3.92	10	3.10E-13
TGME49_060350		0.00	9.82	40.18	13.96	10	7.18E-27
TGME49_061920	helicase, putative	4.83	7.90	20.97	18.22	10	1.23E-19
TGME49_065340	hypothetical protein	10.59	3.28	32.21	23.99	10	1.09E-23
TGME49_066680	hypothetical protein	25.97	17.46	85.74	47.04	10	1.83E-17
TGME49_066940	DHHC zinc finger domain-containing protein	7.50	9.12	26.06	14.15	10	9.13E-14
TGME49_067730	hypothetical protein	3.64	6.75	18.96	10.98	10	3.65E-16
TGME49_067870	NBP2b protein, putative	0.00	3.49	9.79	8.51	10	4.64E-24
TGME49_069310	hypothetical protein	0.00	0.89	8.74	6.07	10	3.47E-24
TGME49_070920	rhoptry kinase family protein ROP32 (ROP32)	17.39	10.93	46.99	29.16	10	2.26E-15
TGME49_076900	hypothetical protein	7.76	13.95	29.21	23.43	10	2.25E-14
TGME49_077230	hypothetical protein	10.55	10.27	32.09	19.92	10	1.36E-13
TGME49_078740	diaminopimelate decarboxylase	2.57	7.27	13.39	11.63	10	1.59E-15
TGME49_080470	AP2 domain transcription factor AP2VIIa-1 (AP2VIIA1)	1.60	3.26	12.54	9.69	10	4.19E-21
TGME49_085280		0.00	4.69	28.77	19.99	10	1.86E-32
TGME49_089720	hypothetical protein	0.00	10.26	27.50	15.92	10	1.85E-25
TGME49_091130	hypothetical protein	0.00	0.92	5.99	2.60	10	9.23E-16
TGME49_091910	hypothetical protein	0.00	0.99	24.32	8.45	10	4.05E-32
TGME49_093270	hypothetical protein	79.16	48.13	240.71	179.24	10	2.98E-19
TGME49_098060	Toxoplasma gondii family C protein	0.00	13.14	23.47	13.59	10	1.59E-24
TGME49_109020	PP-loop family protein	2.25	9.35	15.64	10.19	10	3.29E-15
TGME49_109970		0.00	31.14	50.99	22.15	10	3.84E-25
TGME49_110220	hypothetical protein	3.37	5.66	12.43	11.44	10	1.67E-14
TGME49_110760	protein phosphatase 2C domain-containing protein	10.14	12.86	41.13	35.73	10	7.25E-21
TGME49_113320	hypothetical protein; hypothetical protein	0.00	5.30	14.89	12.93	10	1.20E-28
TGME49_113480	hypothetical protein	1.40	11.86	20.64	16.87	10	1.53E-23
TGME49_114770	tRNA (guanine-N1)-methyltransferase	5.71	7.58	24.81	17.24	10	7.39E-18
TGME49_116670	Toxoplasma gondii family D protein	0.00	4.67	9.17	3.98	10	2.78E-17
TGME49_119540	hypothetical protein	2.56	6.11	13.33	7.72	10	5.21E-13
TGME49_121480	SAG-related sequence SRS12B (SRS12B)	3.38	20.61	41.07	35.68	10	9.19E-25

[a]Data represent RPKM values if not specified otherwise

Supplementary Table S11: Primers sequences

mRNA	Accession No.	Forward primer	Reverse primer
Actb	NM_ 007393	5'- GTACCACCATGTACCCAGGC -3'	5'- AAGGGTGTAAAACGCAGCTC -3'
Cxcl10	NM_021274	5'- CCAAGTGCTGCCGTCATTTT -3'	5'- AGCTTCCCTATGGCCCTCAT -3'
Cyr61	NM_010516	5'- CCTCCTGCGCGCCACAATGA -3'	5'- TCCCGGACCAACCCGACTCC -3'
Dpm1	NM_010072	5'- ATGGAATCAAACACGCCACG -3'	5'- CCCATCCATAAACACCCCCA -3'
Egr2	NM_010118	5'- CCAGGTAGCGAGGGAGTTGGGT -3'	5'- ATGCCATCTCCCGCCACTCCGTT -3'
Fosb	NM_008036	5'- GCTCGTGCAACCCACCCTCA -3'	5'- GGGTAAGTGTCTCTTCTCGGGGT -3'
Nr4a1	NM_010444	5'- CTCCGCCTGGCATACCGATCT -3'	5'- AGGCAGGCAAAGGCGGGAAC -3'
Palm2	NM_172868	5'- TTTTTCCCCTGCCCGTGTGC -3'	5'- CTTCAGACTGCCTGCGCCTGG -3'
Ptgs2	NM_011198	5'- GACAGATTGCTGGCCGGGTTG -3'	5'- TCAGGGAGAAGCGTTTGCGGT -3'
S100a4	NM_011311	5'- CTTGGTCTGGTCTCAACGGT -3'	5'- GTCCCTGTTGCTGTCCAAGT -3'
Serinc4	NM_001025371	5'- AGTTCGCCTCCACTCCCCCA -3'	5'- GCTGCACCAGTCTGCCAATTC -3'
Slc44a4	NM_023557	5'- TGTGACCCGATGGCCCCGTT -3'	5'- CCCGGCAAGGACACACTGGC -3'
Smyd1	NM_009762, NM_001160127	5'- CGCAGATGAGGATTGAGCTCCGGG -3'	5'- TCGCTGCCAGGAAGAGGTCGT -3'

Ttpal	NM_029512, NM_181734	5’- CCGAGGAGACCCAGGTGAACG -3’	5’- CAGCACGGCGTTCCAGGAGG -3’
Ubp1	NM_001083319, NM_013699	5’- CTACAGCATGAGTGATGTGTTGGCA -3’	5’- TCGTGCAGCTTAACGGCTGGC -3’
